# The 2026 Oceania Report of the *MJA–Lancet* Countdown on Health and Climate Change: Children Already Impacted, With a Lifetime of Exposure to Come

**DOI:** 10.5694/mja2.70315

**Published:** 2026-10-28

**Authors:** Paul J. Beggs, Tanya Singh, Hilary Bambrick, Nicolas Borchers Arriagada, Kathryn Bowen, Anthony G. Capon, Philomena Colagiuri, Tara Crandon, Troy J. Cross, Lynette Dimer, Donna Green, Ivan C. Hanigan, Maddie Heenan, Thanh Ho, Ollie Jay, Harry R. Kennard, Fiona Langridge, Martina K. Linnenluecke, Zerina Lokmic‐Tomkins, Raymond Lovett, Calla MacGregor, Arunima Malik, Rebekkah Markey‐Towler, Veronica Matthews, Christopher D. McKay, Celia McMichael, Kerry Nice, Jacqueline Peel, Sachith Seneviratne, Katherine Sievert, James Smallcombe, Mark Stevenson, Stefan Trueck, Sotiris Vardoulakis, Aditya Vyas, Bronwyn Wilkes, Jake T. W. Williams, Alistair J. Woodward, Ying Zhang, Muhammad M. Hasan, Marina B. Romanello

**Affiliations:** ^1^ School of Natural Sciences, Faculty of Science and Engineering Macquarie University Sydney New South Wales Australia; ^2^ National Centre for Epidemiology and Population Health, ANU College of Law, Governance and Policy Australian National University Canberra Australian Capital Territory Australia; ^3^ Menzies Institute for Medical Research University of Tasmania Hobart Tasmania Australia; ^4^ Melbourne School of Population and Global Health, Faculty of Medicine, Dentistry and Health Sciences, Melbourne Climate Futures University of Melbourne Melbourne Victoria Australia; ^5^ School of Public Health, Faculty of Medicine and Health University of Sydney Sydney New South Wales Australia; ^6^ Heat and Health Research Centre, School of Public Health, Faculty of Medicine and Health University of Sydney Sydney New South Wales Australia; ^7^ School of Public Health, College of Health Adelaide University Adelaide South Australia Australia; ^8^ Sydney School of Health Sciences, Faculty of Medicine and Health University of Sydney Sydney New South Wales Australia; ^9^ The Kids Research Institute Australia Perth Children's Hospital Perth Western Australia Australia; ^10^ School of Biological, Earth and Environmental Sciences, Faculty of Science UNSW Sydney Sydney New South Wales Australia; ^11^ WHO Collaborating Centre for Climate Change and Health Impact Assessment, Curtin School of Population Health, Faculty of Health Sciences Curtin University Perth Western Australia Australia; ^12^ The George Institute for Global Health Sydney New South Wales Australia; ^13^ Melbourne School of Design, Faculty of Architecture, Building and Planning University of Melbourne Melbourne Victoria Australia; ^14^ Heat and Health Research Centre, Faculty of Medicine and Health University of Sydney Sydney New South Wales Australia; ^15^ Walker Department of Mechanical Engineering University of Texas at Austin Austin Texas USA; ^16^ Department of Paediatrics: Child and Youth Health, Faculty of Medical and Health Sciences University of Auckland Auckland New Zealand; ^17^ UTS Business School University of Technology Sydney Sydney New South Wales Australia; ^18^ School of Nursing and Midwifery, Faculty of Medicine, Nursing and Health Sciences Monash University Melbourne Victoria Australia; ^19^ Integrated Sustainability Analysis, School of Physics, Faculty of Science, Discipline of Accounting, Sydney Business School University of Sydney Sydney New South Wales Australia; ^20^ Melbourne Law School, Melbourne Climate Futures University of Melbourne Melbourne Victoria Australia; ^21^ University Centre for Rural Health University of Sydney Sydney New South Wales Australia; ^22^ School of Geography, Earth and Atmospheric Sciences, Faculty of Science University of Melbourne Melbourne Victoria Australia; ^23^ School of Health and Social Development Deakin University Melbourne Victoria Australia; ^24^ Department of Infrastructure Engineering, School of Electrical, Mechanical and Infrastructure Engineering, Faculty of Engineering and Information Technology, School of Population and Global Health, Faculty of Medicine, Dentistry and Health Sciences University of Melbourne Melbourne Victoria Australia; ^25^ Department of Actuarial Studies and Business Analytics, Macquarie Business School Macquarie University Sydney New South Wales Australia; ^26^ Health Research Institute, Faculty of Health University of Canberra Canberra Australian Capital Territory Australia; ^27^ Western Sydney Local Health District Sydney New South Wales Australia; ^28^ Yardhura Walani, National Centre for Aboriginal and Torres Strait Islander Wellbeing Research, National Centre for Epidemiology and Population Health, ANU College of Law, Governance and Policy Australian National University Canberra Australian Capital Territory Australia; ^29^ School of Population Health, Faculty of Medical and Health Sciences University of Auckland Auckland New Zealand; ^30^ Lancet Countdown, Institute for Global Health University College London London UK

**Keywords:** climate change, health communication, health financing, health policy, morbidity, mortality, population health, risk management

## Abstract

The *Medical Journal of Australia* (*MJA*)–*Lancet* Countdown on health and climate change in Oceania was established in 2017 and produced its first regional assessment in 2018 and annual updates from 2019 to 2025. It examines five broad domains: (1) health hazards, exposures and impacts; (2) adaptation, planning and resilience for health; (3) mitigation actions and health co‐benefits; (4) economics and finance; and (5) public and political engagement. In this, the eighth report of the *MJA*–*Lancet* Countdown, we track an extensive suite of indicators across these five domains, accessing and presenting the latest data and further refining and developing our analyses. Our analyses show that exposure of infants and older adults to heatwaves in Australia and Aotearoa New Zealand has increased substantially over the past decade due to climate change, and half of the 16,230 young Australians aged 14–19 years surveyed reported that they are worried or sad about the impacts of climate change, including its threat to people, and about 1 in 7 reported that such feelings are negatively affecting their daily life. Increasing heat‐related mortality alone is placing a large economic burden on Australians, with associated inflation‐adjusted costs over the period 2021–2025 being more than double what they were at the start of the century, now averaging an estimated US$1.49 billion per year nationally. In other areas, some progress is being made. New Zealand's Health National Adaptation Plan 2024–2027, published in October 2024, lays the foundation for building climate resilience within its health system, while acknowledging the environmental and social determinants of health that apply more broadly. The provision of climate services to the health sector in both Australia and New Zealand is categorised as advanced. In Australia, healthcare‐related greenhouse gas emissions generally declined from 2016 to 2023. In conclusion, the threat to physical and mental health from climate change continues to grow. Despite pockets of progress, Australia and New Zealand have a long way to go to adequately mitigate and adapt to climate change, and in so doing not only avoid health impacts but seize the opportunity for generational health gains. Important opportunities have been lost, such as the failed attempts to amend Australia's *Climate Change Act 2022* to ‘require decision‐makers to consider the health and well‐being of children in Australia when making significant decisions’. In 2026, Australia is in the spotlight as President of Negotiations for the United Nations Climate Change Conference (COP31)—an unprecedented opportunity for the Australian Government to push through the sizable step change in global climate action that is now critically required.

## 1 Introduction

Climate is intricately connected to human health [1]. Although the thin layer of gases that surrounds the Earth, and the planet's climate, has sustained the development of human civilisations for millennia, human activities are now throwing Earth's climate system increasingly out of balance [2]. Driven by the ongoing use of fossil fuels, the global atmospheric carbon dioxide concentration hit 425.65 ppm in 2025, its highest level in the past 2 million years [3, 4]. The relentless increase in this and other greenhouse gases has resulted in the past 11 years (2015–2025) being the warmest on record, with 2025 being among the top 3 [3].

Climate change is occurring everywhere, including Australia and Aotearoa New Zealand [5]. The year 2025 was Australia's fourth hottest year on record and January 2026 its fourth hottest January. Six consecutive days that month saw at least one Australian location reach 49°C or higher, while Port Augusta, South Australia, became the most southerly place on Earth to record 50°C [6]. The 2025–2026 summer was characterised by rapid shifts from one extreme to the other: climate whiplash [6]. For example, Victorian communities experienced their worst fire danger since the 2019–2020 Black Summer and, a week later, experienced record‐breaking rainfall and flash flooding. In New Zealand, intensifying storms due to climate change are increasing the frequency of landslides, its deadliest natural hazard. In January 2026, tropical storms brought torrential rain to the North Island, triggering two fatal landslides [7, 8].

Evidence and understanding of the impacts of climate change on health in Australia and New Zealand continues to mount. As it does, the gravity of this threat to all people, and especially a large number of particularly vulnerable groups, becomes disturbingly clear [9, 10, 11, 12, 13, 14]. At the same time, there is encouraging engagement with the climate‐health challenge in the health sector [15, 16, 17, 18, 19]. However, the progress that has been made and the future accelerated progress that is needed are now being undermined from multiple directions [20, 21]. The 31st United Nations Framework Convention on Climate Change (UNFCCC) Conference of the Parties (COP31) will be held in Antalya, Türkiye, on 9–20 November 2026. As President of Negotiations [22], Australia has an unprecedented opportunity to get the world on track to effectively limit climate change and adapt to the threats that cannot now be avoided.

With support from the Wellcome Trust, the *Lancet* Countdown is a flagship international academic collaboration that brings together over 300 expert researchers from around the world to track both the health impacts of climate change and the health opportunities of climate action, engaging policymakers and health professionals with the evidence to inform a thriving future. Over its decade of operation, the *Lancet* Countdown has established six regional centres: Africa, Asia, Europe, Latin America, Oceania and Small Island Developing States (SIDS). The Oceania Regional Centre focuses on Australia and New Zealand, with many of the neighbouring small island states covered by the SIDS Regional Centre [23]. The 2026 Oceania report of the *Medical Journal of Australia* (*MJA*)–*Lancet* Countdown presents the work of a collaboration of 41 experts from 18 institutions (15 from Australia, one from New Zealand, one from the United Kingdom and one from the United States), brought together by an established partnership between *The Lancet* and the *Medical Journal of Australia*. The collaboration tracks the links between public health and climate change across five broad domains: (1) health hazards, exposures and impacts; (2) adaptation, planning and resilience for health; (3) mitigation actions and health co‐benefits; (4) economics and finance; and (5) public and political engagement. In this, the eighth report of the *MJA*–*Lancet* Countdown [24, 25, 26, 27, 28, 29, 30], we track progress on an extensive suite of indicators across these five domains, accessing, assessing and presenting the latest data and further refining and developing our analyses.

## 2 Methods

We tracked progress on health and climate change in Australia and New Zealand across 28 indicators within the five broad domains (Table 1). Where possible, we adopted the methods used by the *Lancet* Countdown, including those used in its 2026 global report [31]. Where more appropriate for an Oceania‐focused assessment, or where it was possible to refine or improve on the original method as more precise and comprehensive data or methods were available at the national level, the method for the Oceania indicator was modified or an alternative method was used. As an annual report, it is necessary for the indicators to be based on data that are produced annually and that are available to the authorship team by May each year (preferably for the previous year), such that the required analysis can be completed and the annual manuscript can be written ready for submission to the *MJA* by June. Overall, the report is an indicator synthesis and the strength of inference varies by indicator.

**TABLE 1:** The five sections and 28 indicators of the 2026 *MJA*–*Lancet* Countdown Oceania report by country.

Section	Indicator	Australia	New Zealand
1: Health hazards, exposures and impacts	1.1 Exposure of vulnerable populations to heatwaves	Yes	Yes
	1.2 Heat and physical activity	Yes	
	1.3 Bushfires	Yes	Yes
	1.4 Drought	Yes	Yes
	1.5 Climate suitability for infectious disease transmission	Yes	Yes
	1.6 Mental health and climate change	Yes	
2: Adaptation, planning and resilience for health	2.1 Health adaptation plans and assessments	Yes	Yes
	2.2 Climate information for health	Yes	Yes
	2.3 Detection, preparedness and response to health emergencies	Yes	Yes
	2.4 Migration and displacement	Yes	Yes
	2.5 Aboriginal and Torres Strait Islander health and climate change	Yes	
3: Mitigation actions and health co‐benefits	3.1 Energy systems and health	Yes	Yes
	3.2 Household energy use	Yes	
	3.3 Sustainable and healthy transport	Yes	
	3.4 Emissions from agricultural production and consumption	Yes	Yes
	3.5 Diet and health co‐benefits	Yes	Yes
	3.6 Healthcare sector emissions	Yes	Yes
4: Economics and finance	4.1 Economic losses due to climate‐related extreme events	Yes	Yes
	4.2 Costs of heat‐related mortality	Yes	Yes
	4.3 Country preparedness for the transition to net zero	Yes	Yes
	4.4 Clean energy investment	Yes	
	4.5 Net value of fossil fuel subsidies and carbon prices	Yes	
5: Public and political engagement	5.1 Media coverage of health and climate change	Yes	
	5.2 Scientific articles on health and climate change	Yes	Yes
	5.3 Scientific engagement on the health impacts of climate change	Yes	Yes
	5.4 Government engagement in health and climate change	Yes	
	5.5 Health and climate change research funding	Yes	
	5.6 Health and climate change litigation	Yes	

Abbreviation: *MJA*, *Medical Journal of Australia*.

Following several years of development [27, 28], we introduce two unique indicators. These are an indicator on mental health and climate change (Indicator 1.6) and an indicator on Aboriginal and Torres Strait Islander health and climate change (Indicator 2.5). Another major development in this year's report is presented in the sustainable and healthy transport indicator (Indicator 3.3). Having focused on the growth of electric vehicles in Australia in previous reports, this report focuses on active transport (walking and cycling), quantified annually in Australia's capital cities through a novel method presented for the first time. Drawing on the latest data from the global *Lancet* Countdown, a further four new indicators are included in this report: climate suitability for infectious disease transmission (Indicator 1.5); costs of heat‐related mortality (Indicator 4.2); country preparedness for the transition to net zero (Indicator 4.3); and scientific engagement on the health impacts of climate change (Indicator 5.3). We also include three new text boxes this year. The first explores child health and climate change in Oceania, the second discusses First Nations strengths in the face of climate change and the third provides an overview of Australian healthcare quality and safety standards for climate resilience and environmental sustainability. Building on our profiles of New Zealand in our 2022, 2023 and 2024 reports [27, 28, 29], this report achieves full integration of our coverage of this country, with results for 17 indicators presented alongside those for Australia (Table 1).

Full details of our methods, data and caveats are provided for each indicator in Appendix S1 and are numbered accordingly. Data for 13 of the 28 indicators come from the 2026 *Lancet* Countdown global report analysis [31]. We also briefly summarise the method for each indicator within the text here.

## 3 Section 1: Health Hazards, Exposures and Impacts

Climate‐related health hazards include extreme ambient temperatures, rainfall and run‐off, changes in ocean conditions that are relevant to human health, and bushfires. Exposures denote the timing, duration and intensity of human contact with these hazards; impacts describe the consequences of exposures on human health. In this section, we focus on recent changes in Australia and New Zealand, using multi‐year comparisons to allow for short‐term variability and estimating where possible the influence of climate change on trends. The list of hazards and impacts is not comprehensive: we are limited in this section to subjects that have been studied repeatedly, in comparable fashion, in either or both countries.

### 3.1 Exposure of Vulnerable Populations to Heatwaves (Indicator 1.1)

Climate change is increasing exposure to heatwaves in Australia and New Zealand [32, 33], with adults aged ≥ 65 years and infants (aged < 1 year) particularly susceptible to heat‐related health risks [34, 35]. Heatwaves were defined as at least 3 consecutive days in which minimum and maximum temperatures exceeded the local 95th percentile based on a 1986–2005 baseline [31] (Appendix S1, 1.1). Between 2016 and 2025, infants experienced on average 4.6 heatwave days per infant per year in Australia and 7.4 in New Zealand, representing increases of 66% and 109%, respectively, relative to baseline, with similar increases experienced by older adults (Appendix S1, Table S1; Figure S1).

This indicator also uses ERA5 temperature data [36] to compare observed heatwave days with the number of heatwave days expected without human‐caused climate change. The difference represents the heatwave days attributable to climate change, with country estimates weighted using WorldPop (https://www.worldpop.org/) population data. Across 2021–2025, 65% of heatwave days in Australia and 73% in New Zealand were attributable to climate change (Figure 1; Appendix S1, Table S2). Climate change‐attributable heatwave exposure increased from 1.6 to 2.6 days per person per year in Australia and from 2.7 to 6.2 in New Zealand between 2006–2015 and 2016–2025. These trends highlight the accelerating contribution of anthropogenic climate change to heatwave exposure, reinforcing the need for targeted heat‐health adaptation in both countries.

**FIGURE 1:**
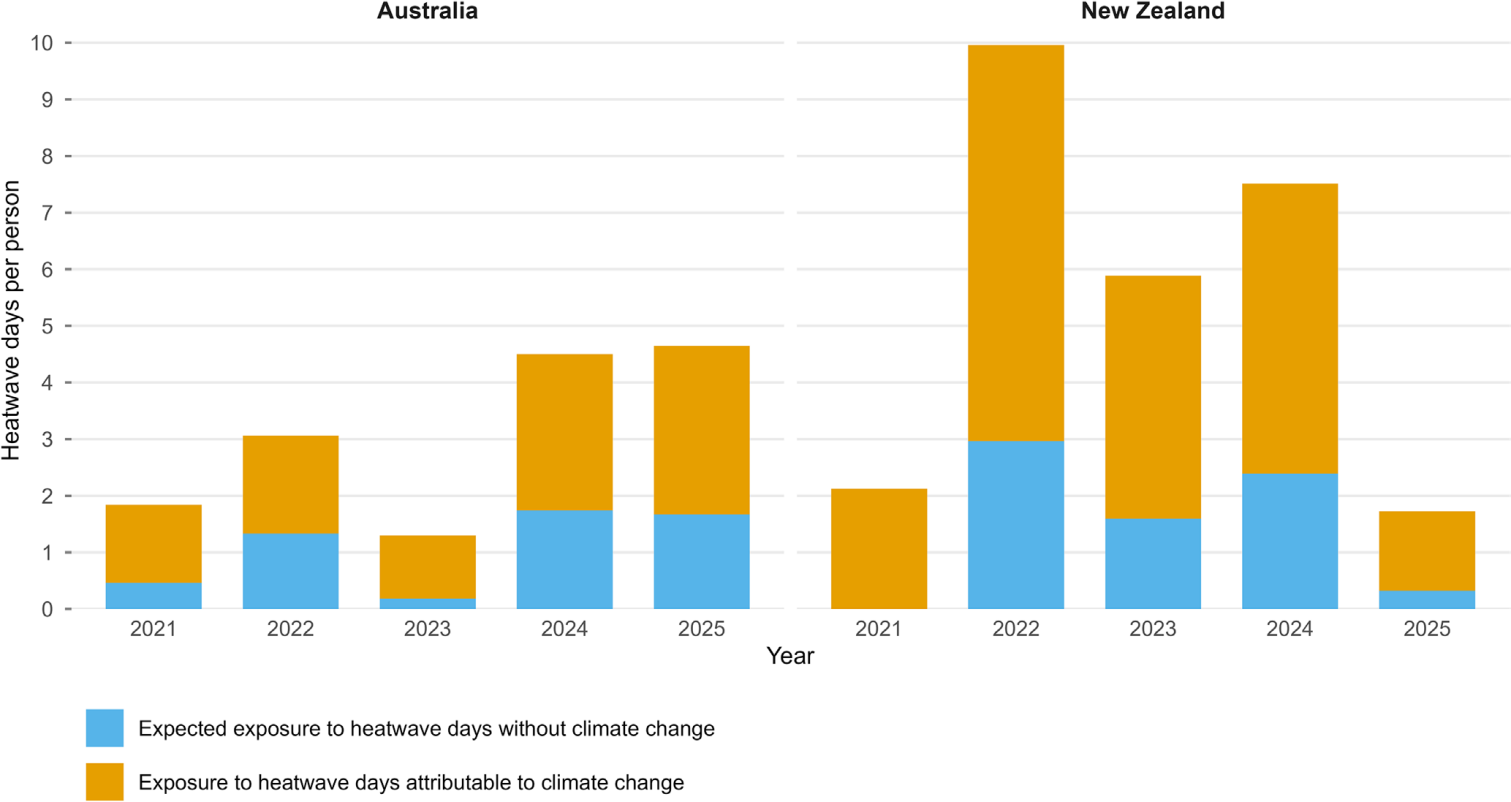
Heatwave days per person per year in Australia and New Zealand, 2021–2025, decomposed into expected exposure in the absence of climate change (blue, lower segment) and additional exposure attributable to anthropogenic climate change (orange, upper segment). The two components sum to the total observed average heatwave exposure. Estimates are population weighted and not stratified by age groups.

### 3.2 Heat and Physical Activity (Indicator 1.2)

Regular physical activity is associated with a wide range of health and environmental benefits, including lower rates of chronic disease [37], better mental well‐being [38] and reductions in greenhouse gas emissions and air pollution when active transport options such as walking and cycling are used [39]. Despite these benefits, rising exposure to heat stress is increasingly limiting opportunities for safe outdoor activity [40], contributing to lower participation and a greater likelihood of heat‐related illness during physical activity [41]. This indicator integrates population data with measures of ambient temperature, humidity and solar radiation to quantify annual hours of heat stress risk encountered during moderate‐intensity outdoor activities [42] (Appendix S1, 1.2).

In 2025, people in Australia were, on average, exposed to 480 h during which the environmental conditions posed at least a moderate level of heat stress risk during moderate‐intensity outdoor physical activity (e.g., brisk walking), representing an increase of 47.5% compared with the 1990–1999 baseline period. Within this, exposure to extreme heat stress risk conditions totalled 16 h, a 90.1% increase relative to 1990–1999, representing periods during which moderate‐intensity outdoor activity would be recommended to cease due to the elevated risk of heat‐related illness.

### 3.3 Bushfires (Indicator 1.3)

Fire activity is shaped by interacting drivers, including vegetation and fuel characteristics; climate‐related factors, such as temperature extremes and drought; and broader influences, such as land‐use practices and ignition sources. Climate change is likely to amplify these conditions, increasing the frequency and severity of extreme bushfires and exacerbating associated health impacts, including direct injuries, smoke‐related morbidity and longer term mental health effects [43, 44].

This indicator integrates three sub‐indicators to assess climate‐related human exposure to bushfires: bushfire exposure (Figure 2A), fire danger days (Figure 2B) and bushfire‐related fine particulate matter (PM_2.5_) exposure (Figure 2C) [29] (Appendix S1, 1.3). Table S3 in Appendix S1 compares Australia and New Zealand between 2006–2015 and 2016–2025. Although decadal averages indicate increases in Australia, these are driven by the unprecedented fire activity during the 2019–2020 season. Overall, as reflected by the trendlines in Figure 2, trends are near‐neutral and uncertain. New Zealand experienced reduced bushfire exposure but increased fire danger days, although still below 1 day per year. Despite recent variability, key climate‐driven risk factors suggest bushfire activity and health impacts may intensify in the future [43].

**FIGURE 2:**
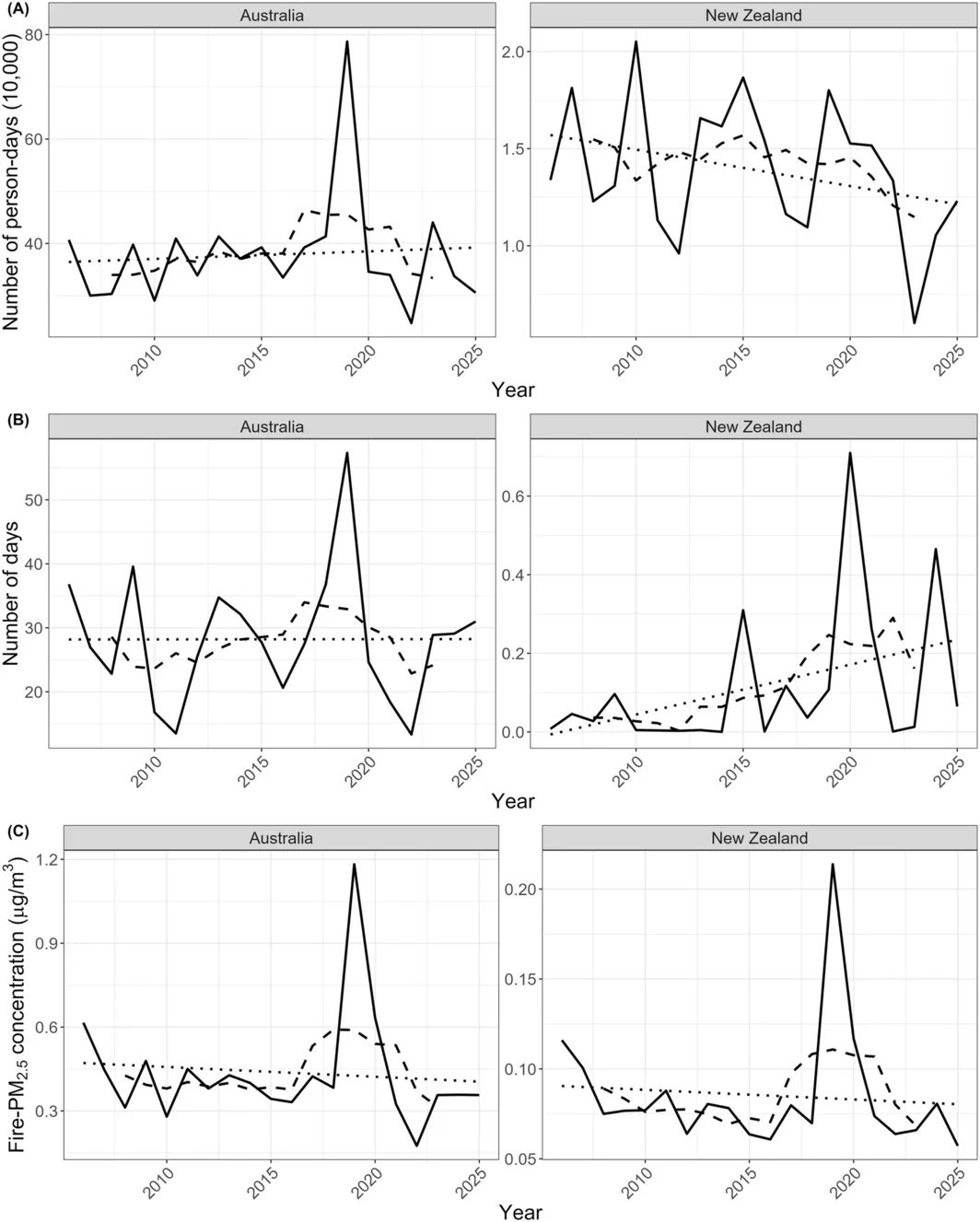
Annual estimated (A) bushfire exposure (person‐days), (B) bushfire risk (days) and (C) bushfire‐related fine particulate matter (PM_2.5_) concentration (μg/m^3^) for Australia and New Zealand during 2006–2025. Showing 5‐year running means with dashed lines (plotted on the middle year) and trends with dotted lines.

### 3.4 Drought (Indicator 1.4)

Extreme drought impacts health through multiple pathways. Health endpoints include worsening mental health [45] and suicide risk [46]; drought compounds the impact of concurrent fires and floods to increase risk of suicidal thoughts in young Australians [47]. Furthermore, severe drought increases the risk of sexual violence against adolescent girls and young women [48]. Drought exposure was measured as the percentage of land area experiencing at least 6 months of extreme or exceptional drought annually (Standardised Precipitation–Evapotranspiration Index [SPEI] < −1.6) [31] (Appendix S1, 1.4). Using this definition, 3% of the area of Australia experienced 6 or more months of drought in 2025, whereas New Zealand had 0% in that drought category (Figure 3). By contrast, the 2019 flash drought covered 44% of Australia's land area; conditions for which neither health systems nor communities were adapted.

**FIGURE 3:**
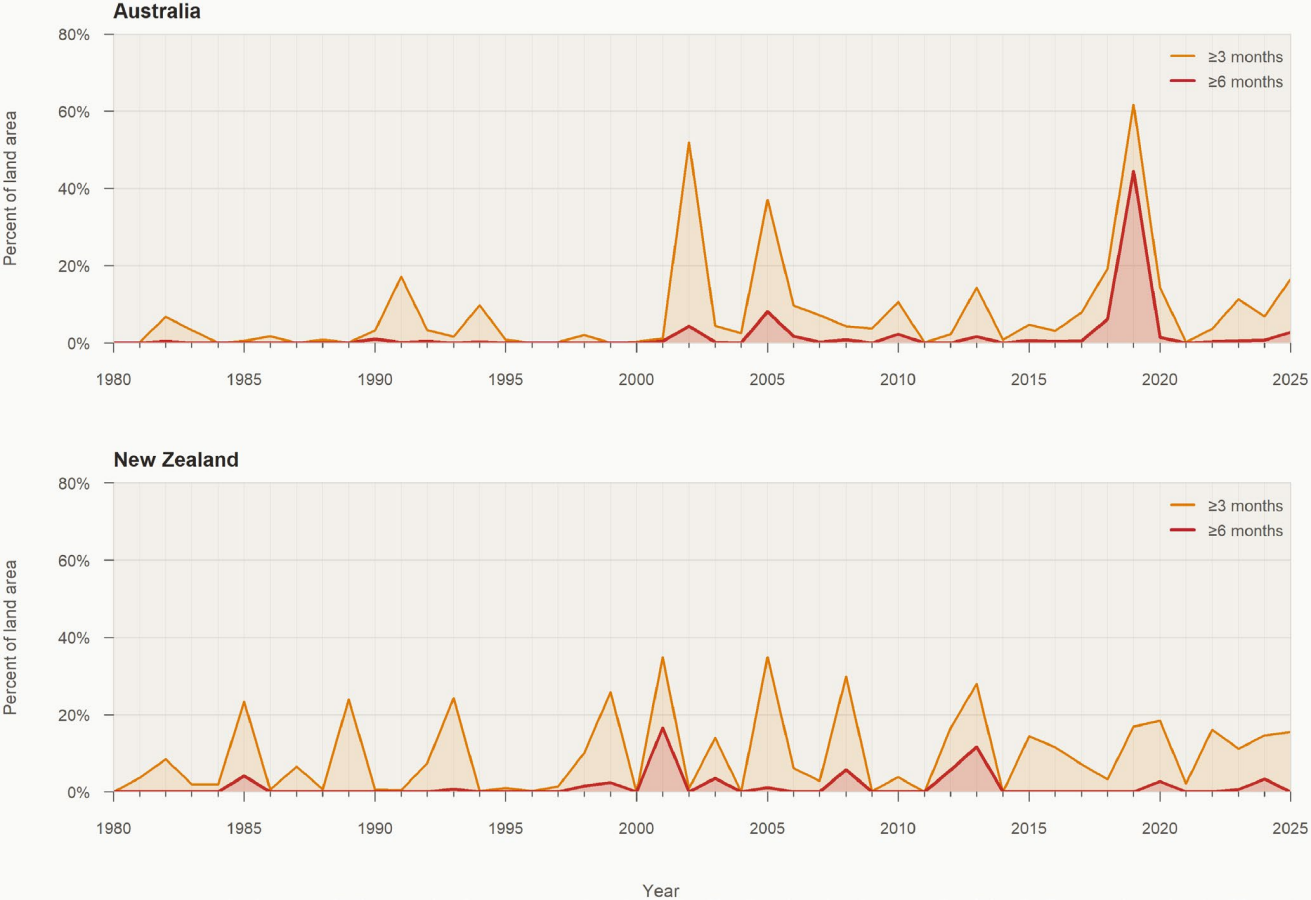
Percentage of land area experiencing extreme drought conditions in Australia and New Zealand, 1980–2025. Extreme drought is defined using a standardised drought index threshold, with the two lines representing the percentage of land area that experienced extreme drought conditions for at least 3 out of 12 months (orange) or at least 6 out of 12 months (red) within a given year.

### 3.5 Climate Suitability for Infectious Disease Transmission (Indicator 1.5)

Dengue is caused by a virus that is transmitted from one person to another by infected mosquitoes [49]. Although dengue is not currently endemic in Australia or New Zealand, both countries experience cases that were acquired overseas, and the spread or introduction of dengue mosquitoes due to climate change poses a serious risk [50, 51]. This indicator tracks the annual absolute basic reproduction number (*R*_0_) for *Aedes aegypti* and *Aedes albopictus* mosquitoes in Australia and New Zealand from 1951 to 2025 (Appendix S1, 1.5). Despite New Zealand continuing to have no known local transmission risk [52], the *R*_0_ for both mosquito species in Australia has increased, being on average 60% and 59% higher for *A. aegypti* and *A. albopictus*, respectively, in 2016–2025 compared with the baseline period 1951–1960 (Figure 4). This concerning trend calls for increased disease, vector and virus surveillance and reporting, and planning to respond to and control potentially larger outbreaks into the future [50, 53].

**FIGURE 4:**
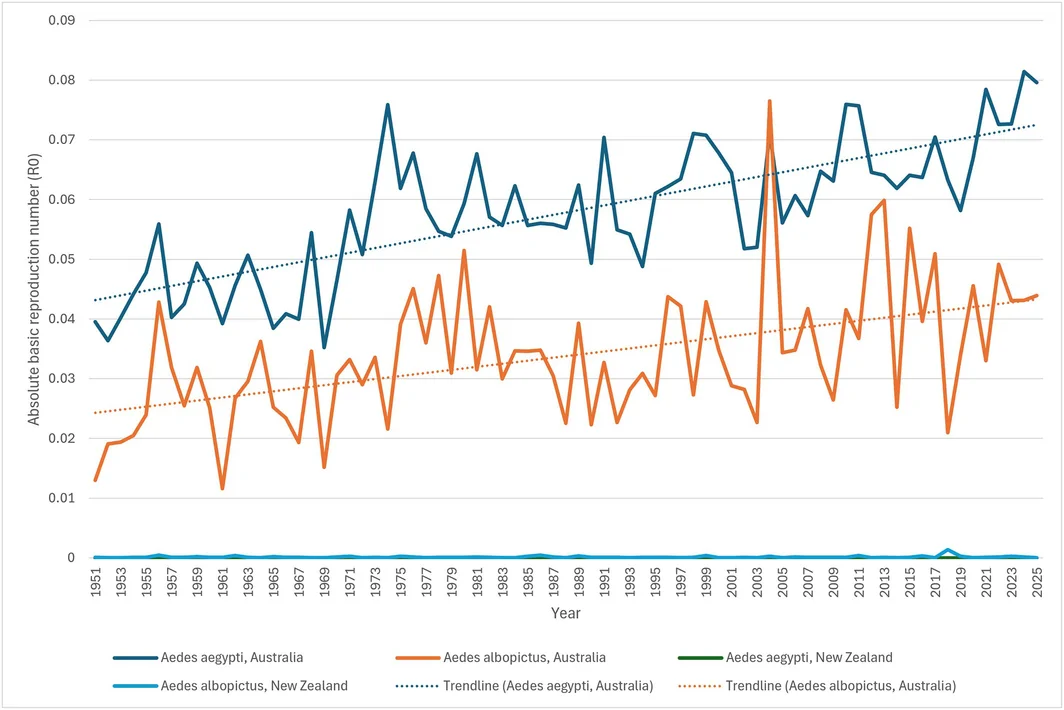
The annual absolute basic reproduction number (R_0_) for *Aedes aegypti* and *Aedes albopictus* mosquitoes in Australia and New Zealand from 1951 to 2025.

### 3.6 Mental Health and Climate Change (Indicator 1.6)

The psychological impacts of climate change on children and youth are receiving increasing attention [54, 55]. Young people (often defined as up to 25 years) face a longer lifetime exposure to climatic changes, which could predispose them to unique mental health impacts [56]. This year, we present data on how concerned youth feel about aspects of climate change.

This is a youth survey indicator of climate concern and perceived daily impact. Data were drawn from Mission Australia's annual Youth Survey, sourced with permission from the climate change report developed by Orygen and Mission Australia [56] (Appendix S1, 1.6). In a sample of 16,230 young people aged 14–19 years, 49% of young people reported feeling worried that climate change threatens people and the planet (Figure 5). About 55% of the young people surveyed reported that they felt sad about species going extinct and animals suffering because of climate change. In addition, 13.5% of the young people surveyed reported that their feelings about climate change negatively affect their daily life.

**FIGURE 5:**
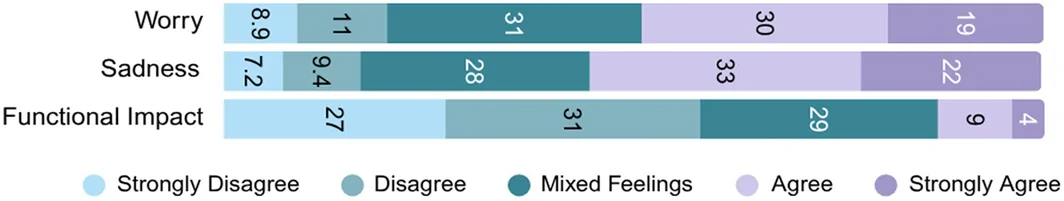
Australian youth climate change concern, sadness and functional impact in 2025. Likert responses of young people aged 14–19 years (*n* = 16,230) to the following questions: (i) ‘I feel worried that climate change threatens people and the planet’; (ii) ‘I feel sad about species going extinct and animals suffering because of climate change’; and (iii) ‘My feelings about climate change negatively affect my daily life’ (at least one of the following: concentrating, school/work, sleeping, having fun, relationships). Data source: Gao et al. [56].

Given that the Mission Australia Youth Survey does not predominantly focus on climate change, the data available on how youth are impacted remain limited. Feelings about climate change will oscillate in salience, valence (pleasant to unpleasant emotions), arousal, duration and intensity. Though this indicator presents data on how worried and sad young people feel about climate change, there could be additional emotional experiences and mental health impacts at play. Furthermore, a plethora of socio‐ecological factors shape how youth feel about and cope with climate change, and these influences could not be captured or analysed.

### 3.7 Summary

Trends vary from one hazard to another. However, in general, there have been increases in both the frequency of hazardous events and impacts on human health. For instance, over the past 10 years, exposure to heatwaves has increased in both countries (there was more than a doubling of heatwave days per year in New Zealand). Our analysis provides robust evidence that climate change is responsible for most of the heatwave exposure in recent years, and extreme heat now limits behaviours regarded as central to human well‐being (such as moderate physical activity outdoors). Bushfire frequency and severity are on the rise in Australia (though not New Zealand). Perhaps of greatest concern, half of all children surveyed reported that they were worried and sad about climate change and almost 1 in 7 children surveyed reported that their feelings about climate change negatively affected their daily lives. Box 1 expands on this topic, providing a more in‐depth exploration of child health and climate change in Oceania.

BOX 1 **Child Health and Climate Change in Oceania.**

**Table:** 

Climate change is shaping child health in Australia and New Zealand through rising temperatures, increasingly frequent and severe extreme weather events and worsening environmental conditions [14, 57]. Children are biologically, developmentally and socially more vulnerable than adults, meaning that climate change functions as a core child health issue rather than an environmental add‐on [58, 59, 60]. Risks begin in pregnancy and early life, accumulate across childhood and adolescence and intersect strongly with social disadvantage, shaping long term health and well‐being trajectories. Studies demonstrate associations between temperature extremes and increased healthcare utilisation, particularly among infants and young children. The perinatal period is a critical window of vulnerability, with evidence linking prenatal heat exposure to adverse perinatal outcomes [61]. Air pollution acts as a risk multiplier. Even low‐level exposure antenatally is associated with adverse birth outcomes [62], and across the life course contributes to respiratory morbidity [63]. Bushfires have exposed millions of Australian children to harm, including respiratory morbidity and psychological distress [64, 65]. In New Zealand, the 2023 extreme weather events resulted in widespread housing damage, school closures and displacement, with children and young people reporting negative effects on their mental well‐being [66, 67]. The consequences of such climate‐related disasters frequently extend beyond immediate recovery, affecting mental health, educational engagement and family stability, particularly with cumulative exposure [68]. Climate change both reflects and exacerbates existing inequities. Children experiencing disadvantage face higher exposure and fewer protective resources, while contributing least to climate change [14]. Despite this, children's needs are often insufficiently centred in climate policy and planning and evidence for child‐focused post‐disaster interventions remains limited [69]. The evidence points to opportunities for action. Parents, caregivers, health professionals, educators, schools and communities have a critical role in reducing children's exposure to climate‐related risks, supporting well‐being, improving safety and advocating for systems‐level change [70, 71]. Children and young people are not only vulnerable to climate change but are increasingly active in responding to it. When appropriately supported, engagement in climate education, advocacy and collective action can foster agency, coping and hope [72, 73]. Effective responses must therefore prioritise the voices and lived experiences of children and young people, particularly those most affected, and be grounded in Indigenous knowledge systems and self‐determination. Taken together, the evidence positions climate change as a life‐course challenge for child health in Australia and New Zealand. A coordinated, child‐centred response that supports children and young people as both those affected and those shaping solutions is essential to prevent climate‐related harms from defining the health of current and future generations.

## 4 Section 2: Adaptation, Planning and Resilience for Health

Protecting human health from climate change through risk assessment and planning requires the collection and availability of quality climate data and capacity to respond to climate‐related health emergencies and emerging threats. As extreme weather occurs over increasingly large areas and across multiple seasons, ever larger numbers of people are potentially at risk. Climate change also alters the ways in which people can use and relate to land. This is the first report to try to capture some of the impact that climate change is having on Aboriginal and Torres Strait Islander health and well‐being, through a measure of access to Country.

### 4.1 Health Adaptation Plans and Assessments (Indicator 2.1)

Climate change and health adaptation plans and assessments are essential to avoid the impacts of climate change on human health. This indicator tracks progress in this area in Australia and New Zealand (Appendix S1, 2.1). Australia's first National Adaptation Plan [74], published together with the National Climate Risk Assessment in September 2025 [32], identified national priorities for adaptation in seven systems including ‘health and social support’. In New Zealand, the Health National Adaptation Plan published in October 2024 laid the foundation for building climate resilience within the health system, while acknowledging the environmental and social determinants of health that apply more broadly [75]. This stemmed from the broader New Zealand National Adaptation Plan published in 2022 covering the period 2022–2028, which identified development of the Health National Adaptation Plan as one of three critical actions for communities [76].

The Australian National Health and Climate Strategy [77] committed the Australian Government to 23 actions related to adaptation, including the development of a Health National Adaptation Plan to support the effective implementation of these actions. It is expected this Plan will be published in 2026. A recent systematic mapping review has highlighted the need for more whole‐of‐system adaptation interventions in Australia, focused on health workforce and service delivery as well as community health and well‐being [78].

### 4.2 Climate Information for Health (Indicator 2.2)

Climate information and services are crucial to effectively anticipate and respond to climate‐related health risks and to assist public health planning and decision‐making [79, 80]. This indicator uses data from the World Meteorological Organization's (WMO) Climate Services Dashboard to track the provision of climate services to the health sector in Australia and New Zealand in 2019 and 2024 (Appendix S1, 2.2). Australia and New Zealand have overall ‘advanced’ climate services capacities, with Australia having progressed to this level from the mid‐range ‘essential’ in 2019 and New Zealand having maintained this highest level since 2019. This places both countries among just 23 of 193 WMO member countries to achieve this highest level. Both countries provide climate services to the health sector across all six services assessed: data services, climate monitoring, climate analysis and diagnostics, climate projections, climate change projections and tailored products.

### 4.3 Detection, Preparedness and Response to Health Emergencies (Indicator 2.3)

Weather and climate cause or contribute to many public health emergencies and the spread of many infectious diseases. This indicator tracks Australia's and New Zealand's reported implementation of the International Health Regulations health emergency management capacity and zoonotic diseases capacity from 2021 to 2024 [81, 82, 83] (Appendix S1, 2.3; Figure S2). Both countries reported having high (80%) implementation of the health emergency management capacity in 2024, just above the regional and global average implementation (76% and 71%, respectively), and the same as in 2023. Australia's zoonotic diseases capacity implementation jumped to high (80%) in 2024, with this being well above the regional and global average implementation of this capacity (57% and 65%, respectively). However, New Zealand reported only medium (60%) implementation of the zoonotic diseases capacity in 2024, slightly above the regional average but below the global average implementation of this capacity.

### 4.4 Migration and Displacement (Indicator 2.4)

Climate change impacts contribute to population displacement, migration and relocation, with consequences for health [10]. This indicator tracks population exposure to future sea level rise of 1 m and displacement of people due to weather‐related disaster (Appendix S1, 2.4). In 2025, 171,011 people in Australia and 48,037 people in New Zealand lived less than 1 m above sea level. Coastal adaptation responses to sea level rise include coastal protection (e.g., seawalls, nature‐based solutions), accommodation of risk (e.g., flood proofing, early warning systems), land reclamation and, typically as a last resort, retreat (e.g., planned relocation of people, assets and infrastructure away from sites of climate risk) [84, 85].

Weather‐related disasters (floods, storms, bushfires) displaced 32,177 people in Australia in 2024, including 25,000 due to flooding in Queensland. In New Zealand, 1672 people were displaced in 2024 by weather‐related disasters (bushfires, erosion, flooding) [86]. Between 2008 and 2024, there is an upward trajectory in disaster displacement, adjusting for population increase, but with substantial annual variation (Figure 6). Climate‐related displacement can lead to increased risks to health including for mental health, infectious disease, nutrition and healthcare access [89]. With increasing frequency and severity of extreme weather events [90, 91], disaster risk planning and management must prepare for forced displacement and adverse health outcomes [89].

**FIGURE 6:**
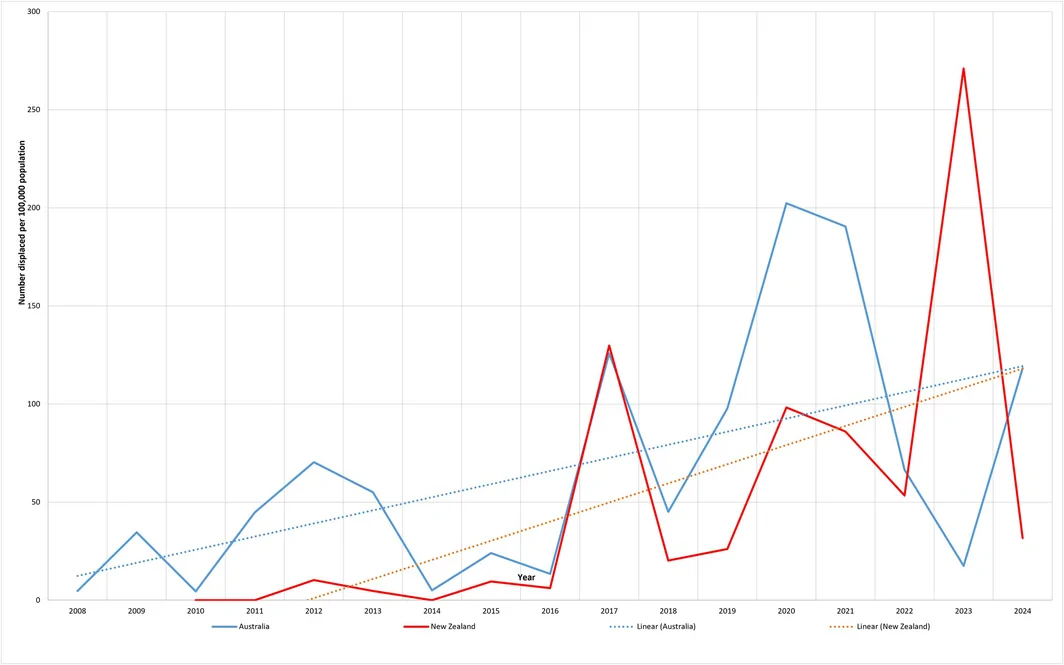
Population displacement rates due to weather‐related disaster in Australia and New Zealand, 2008–2024. Data sources: Internal Displacement Monitoring Centre [86] and World Bank Group [87, 88].

### 4.5 Aboriginal and Torres Strait Islander Health and Climate Change (Indicator 2.5)

Healthy Country and the ability to practice culture are central to Aboriginal and Torres Strait Islander well‐being [92]. Settler‐colonisation and climate change continue to negatively affect the health of Country, as well as Aboriginal and Torres Strait Islander peoples' access to Country and capacity to practise culture [93]. This indicator tracks Aboriginal and Torres Strait Islander peoples' self‐reported access to Country (Appendix S1, 2.5).

Between 2021 and 2025, an estimated 58.5% (95% confidence interval [CI], 56.7%–60.3%) of Aboriginal and Torres Strait Islander adults spent time on Country, whereas 14.3% (95% CI, 13.1%–15.6%) wanted to spend time on Country but were unable to. Spending time on Country was more prevalent among people living in remote areas, whereas a higher proportion of those living in urban areas compared with remote areas wanted to spend time on Country but could not (Figure 7).

**FIGURE 7:**
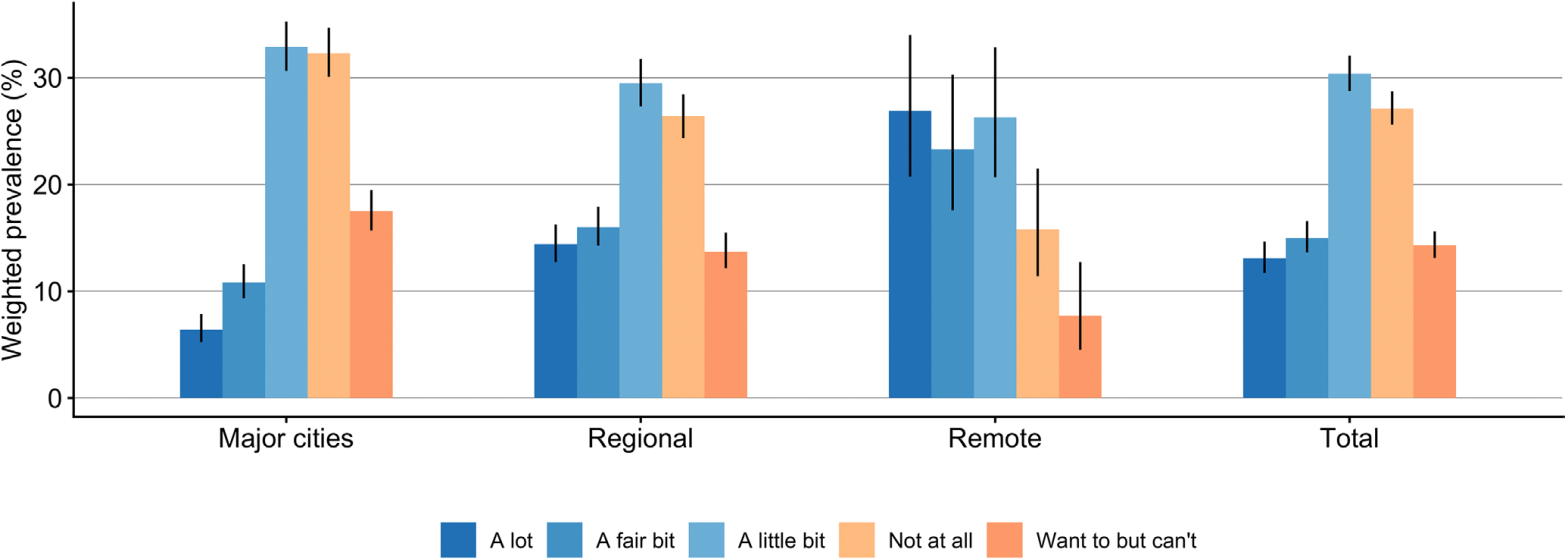
Weighted prevalence estimates and 95% confidence intervals of time spent on Country by Aboriginal and Torres Strait Islander adults (2021–2025), overall and by remoteness. Data source: Mayi Kuwayu—The National Study of Aboriginal and Torres Strait Islander Wellbeing, Wave 2 Release 7.1 data file.

These findings suggest that Aboriginal and Torres Strait Islander adults living in urban areas may face greater barriers to accessing Country. This may reflect that people in urban settings are less likely to live on or near their traditional Country and may experience greater disconnection from culture and Country due to settler‐colonial policies and practices.

Although this indicator does not measure climate‐attributable loss of access to Country, some communities have expressed increasing concern about the impacts of climate change on Country (Box 2). For example, the increasing intensity and duration of heatwaves in remote Central Australia are reducing opportunities to conduct ceremony and care for Country. Indigenous Knowledges are critical to informing place‐based climate adaptation strategies that strengthen Country and support Aboriginal and Torres Strait Islander well‐being.

BOX 2 **First Nations Strengths in the Face of Climate Change.**

**Table:** 

Stories of environmental change, the challenges they present and adaptive responses led by Aboriginal communities are being documented through the Healing Country project. Across three case studies—urban (Whadjuk Noongar Boodja), regional (Bundjalung Jagun) and remote (Warumungu Manu)—Elders, Knowledge Holders and community members are sharing lived experiences that continue ancestral traditions of observing Country. These stories have long informed environmental understanding and shaped relationships with land and waterways over millennia. Monumental shifts in Earth's history remain embedded in living stories and songlines passed through generations. One example is ‘Mammung: the Whale Story’, featuring intimate knowledge of cultural locations on the continental shelf, submerged following deglaciation and sea‐level rise some 17,000–20,000 years ago [94, 95]. Although Western science has only recently documented cultural artefacts off Western Australia using remote sensing [96], Aboriginal peoples have long carried this knowledge. Historical climate shifts were relatively slow moving in the context of anthropogenic climate change, allowing communities to adapt through mobility and negotiation with neighbouring groups, while maintaining cultural governance systems and values of caring for Country and each other. Across all case‐study sites, access to Country emerged as a central challenge: industrialisation, urbanisation, agricultural development and land degradation restrict access to traditional lands and increasingly intense bushfires, floods and prolonged heatwaves limit time spent on Country. Together, these pressures are reducing the availability of natural resources once carefully managed and shared across communities. On flood‐prone Bundjalung Jagun, Aboriginal ranger groups are utilising Traditional Knowledge systems to restore riparian habitats and reintroduce cultural burning. However, such initiatives depend on coordinated, large‐scale approaches that are often constrained by fragmented governance, funding and land tenure arrangements [97]. For Bundjalung mob, well‐being is grounded in keeping cultural stories and practice alive and educating all people about relational obligations to care for and heal Country [97, 98]. Across all sites, re‐telling cultural stories to young people was emphasised as critical to intergenerational health and well‐being. Whadjuk Noongar community emphasised teaching children on Country through seasonal observations and cultural practice [99]. Bringing Elders into classrooms is important where access is constrained [100]. On Warumungu Country, junior ranger programs are vital pathways for Elders to pass on knowledge [101]. On‐Country sharing of stories sustains cultural continuity and mobilises climate adaptation knowledge, underscoring the importance of climate justice, self‐determination and collaboration as foundations for well‐being and enduring responses to climate change.

### 4.6 Summary

Significant gains have been made in risk assessment and adaptation planning with the release of Australia's National Climate Risk Assessment and National Health and Climate Strategy, while New Zealand has had a Health National Adaptation Plan since 2024. The quality of climate information underpinning Australia's capacity for adaptation planning has also improved to the highest standard, now on par with New Zealand. Both countries are generally well placed to respond to health emergencies, with Australia's capacity in zoonotic diseases especially strong. Weather‐related displacement of people in Australia is highly variable year to year and is trending upwards. For Aboriginal and Torres Strait Islander peoples, there is increasing concern about impacts on Country, with reduced opportunities for ceremony and care for Country.

## 5 Section 3: Mitigation Actions and Health Co‐Benefits

In addition to reducing future hazardous exposures and health impacts, climate change mitigation can deliver substantial immediate health co‐benefits by improving air quality, supporting healthier diets and increasing physical activity. This section examines mitigation trends across energy systems, household energy use, transport, agriculture, food systems and healthcare in Australia and New Zealand. The indicators provide insight into how mitigation efforts are progressing across sectors and contributing to healthier and more equitable communities, particularly in relation to energy access, urban design, food systems and exposure to pollution. The section focuses on the close links between decarbonisation pathways and population health outcomes.

### 5.1 Energy Systems and Health (Indicator 3.1)

Energy systems remain the dominant source of global greenhouse gas emissions and drive climate change while posing major air pollution and public health risks. As in previous reports, we define the carbon intensity of the energy system as the tonnes of carbon dioxide (CO_2_) emitted for each unit of primary energy supplied [26] (Appendix S1, 3.1). The latest data now extend to 2023.

Global CO_2_ emissions from fuel combustion declined slightly in 2023 to around 35,559 megatonnes, with global carbon intensity steady at 54.8 tCO_2_/terajoule. Australia recorded some progress, with carbon intensity falling to 64.6 tCO_2_/terajoule in 2023 from 66.6 tCO_2_/terajoule in 2022, though it remains among the highest analysed alongside China (66.1 tCO_2_/terajoule). New Zealand's carbon intensity is substantially lower than most of the countries analysed and the global value—in part due to the high contribution of hydropower to its total energy supply compared with many other countries—and has been relatively stable over the past decade or so (Figure 8).

**FIGURE 8:**
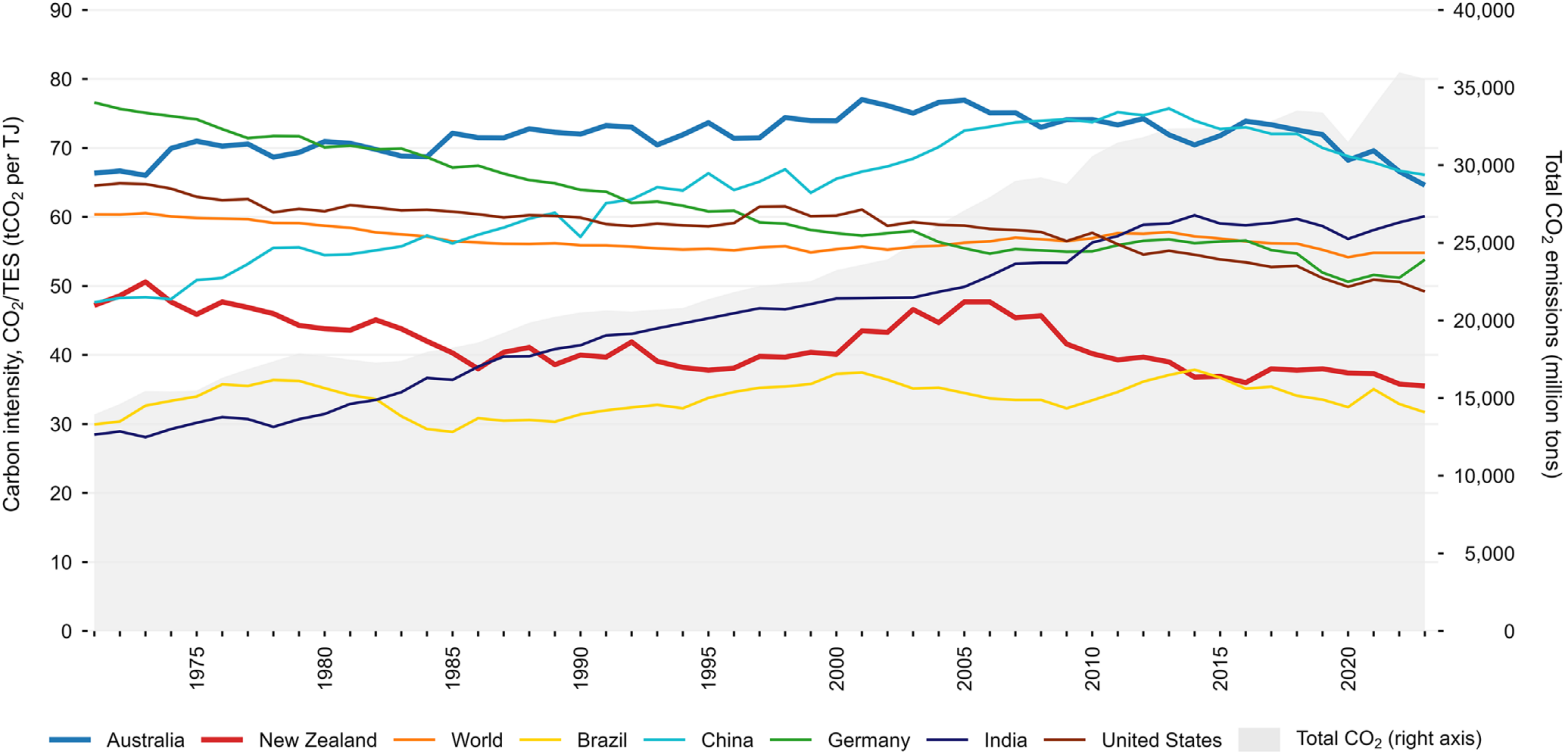
Carbon intensity of total primary energy supply for Australia, New Zealand and selected countries and total carbon dioxide (CO_2_) emissions from fuel combustion. Data source: International Energy Agency (IEA) [102].

Australia's energy supply remains heavily fossil‐fuel dependent. Achieving net zero by 2050 will require accelerated renewable deployment, grid investment, workforce reskilling and significant investments in a just transition.

### 5.2 Household Energy Use (Indicator 3.2)

Using Clean Energy Council data, this indicator monitors renewable contributions to Australia's electricity supply and the uptake of distributed energy resources at the household level (Appendix S1, 3.2). In 2024, 40% of Australia's electricity came from renewables, continuing an upward trend from 20% in 2019 [103, 104]. Australia surpassed 4 million rooftop solar installations in 2024, and home battery sales rose to around 75,000 units, up from 46,127 in 2023 [104, 105].

Despite these advancements, the First Nations Clean Energy Strategy 2024–2030 recognises First Nations peoples as among the most energy‐insecure populations in Australia, with disaggregated data on remote and Indigenous community access remaining limited [106].

In addition, households without access to capital, suitable roof space or home ownership (including the nearly 3 million renting households in Australia) remain largely excluded from the bill savings that rooftop solar and storage provide [105, 107].

### 5.3 Sustainable and Healthy Transport (Indicator 3.3)

Active transport is an important mitigation action and is known to enable improved health outcomes [108]. Developed and presented for the first time, this indicator uses computer vision‐based surveillance of pedestrians and cyclists in the central business district (CBD) and inner and outer suburbs of Australian capital cities (except Darwin) from 2022 to 2025. We leveraged crowdsourced Mapillary street view images to analyse pedestrian and cycling uptake as well as spatial density of these transport modes (Appendix S1, 3.3; Figures S3–S9 and S12–S15). Analysis of images from Melbourne and Sydney consistently indicated increased detection rates of active transport, especially within the central CBD and adjacent areas (Table S4). Detection of cycling in images from Melbourne CBD increased almost sixfold from 2022 to 2025 (Figure 9), which is consistent with the sustained infrastructure investment in Melbourne over the period. Similar increases were seen in analysed images from Canberra and Adelaide CBDs, whereas detection of cycling in images from Hobart CBD decreased over this period (Appendix S1, Figure S11). Across all cities, image analyses suggested a decrease in active transport activity when moving further away from the centre of the city, highlighting the importance of the urban form in enabling physical activity for its residents (Figure 9; Appendix S1, Figures S10 and S11, and Table S4).

**FIGURE 9:**
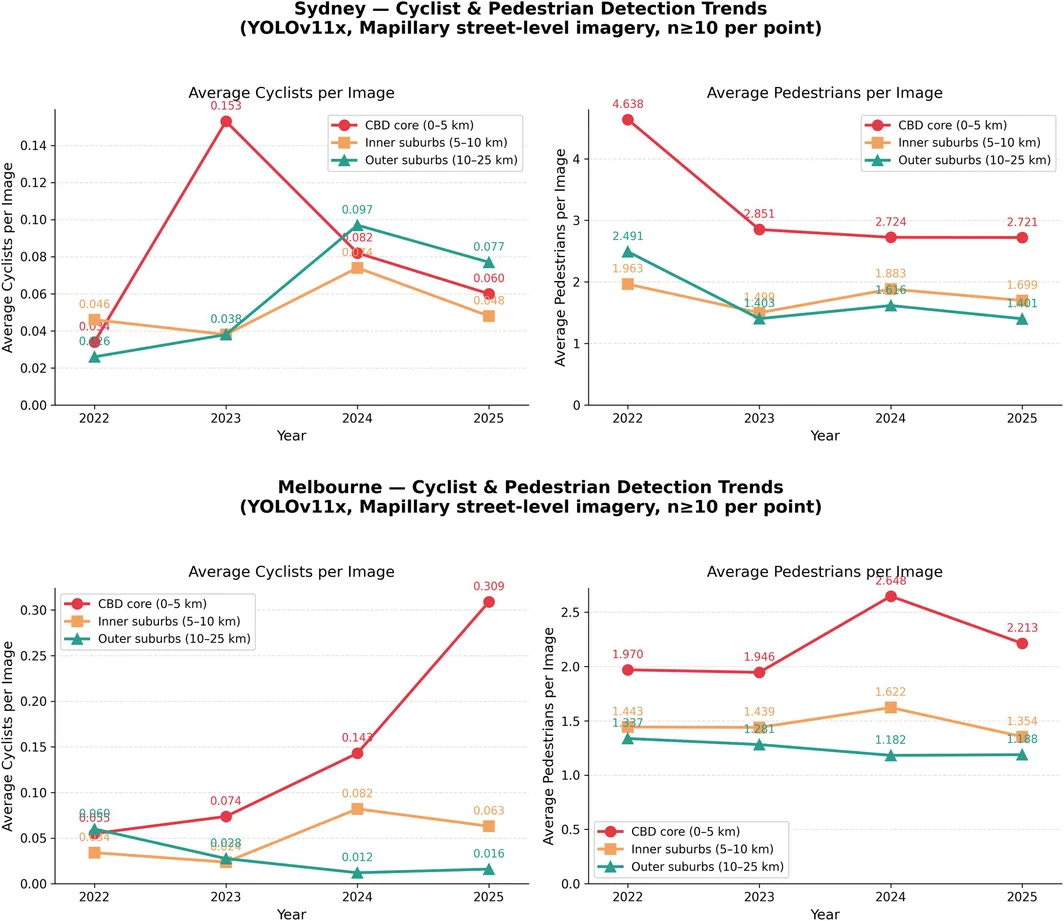
Sydney and Melbourne average cyclists and pedestrians per human‐containing image by zone, 2022–2025. CBD, central business district.

As a novel method presented for the first time, we wish to highlight a number of likely biases and weaknesses in it. The analytical workflow inherits the weaknesses of the imagery provider (Mapillary), such as inconsistent coverage (of different routes, times of the day, seasons, etc.) and poor representation of outer urban and rural areas. The detection performance of the model is known to vary with image quality, crowd density and occlusion.

### 5.4 Emissions From Agricultural Production and Consumption (Indicator 3.4)

This indicator tracks greenhouse gas emissions from agricultural production and consumption over time (Appendix S1, 3.4, Figure S16). In Australia, emissions are dominated by beef and lamb production, which accounted for 84% of agricultural emissions in 2023 (Figure 10). In New Zealand, emissions are dominated by dairy products and beef and lamb production, which accounted for 98% of its agricultural emissions in 2023.

**FIGURE 10:**
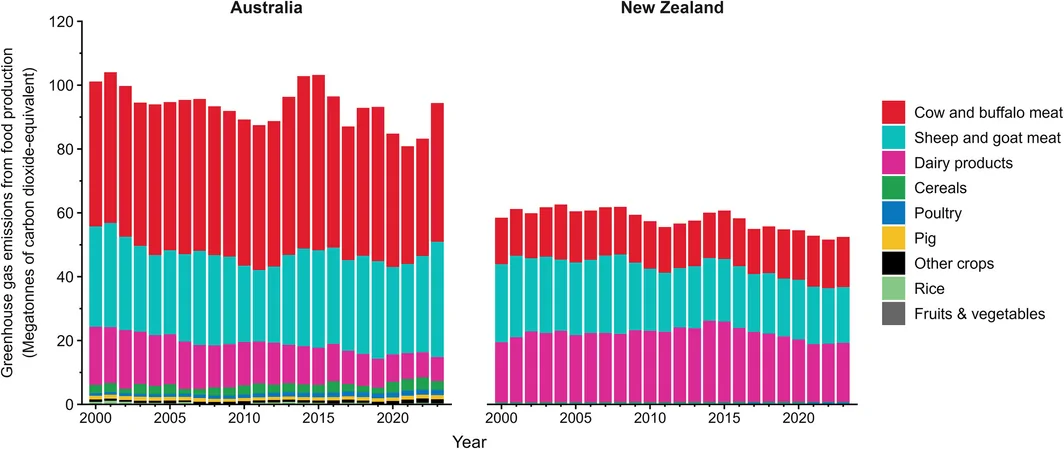
Annual greenhouse gas emissions from food production in Australia and New Zealand by commodity from 2000 to 2023.

New Zealand's agricultural emissions are lower at about 52 megatonnes in 2023, compared with about 94 megatonnes in Australia. However, New Zealand's population is just a fifth that of Australia's and its relatively large agricultural emissions are mainly due to New Zealand's very high production volumes for export markets. Although New Zealand has established emissions reduction targets for agriculture, in 2024, the government removed agricultural products from the country's emissions trading scheme, demonstrating tensions between climate goals, economic productivity and public health.

### 5.5 Diet and Health Co‐Benefits (Indicator 3.5)

Population diets are shaped by food systems and have implications for health, environmental sustainability and equity. This indicator uses comparative risk assessment methods, drawing on meta‐analyses of diet–disease relationships, dietary intake, bodyweight trends and population data to estimate diet‐attributable mortality (Appendix S1, 3.5). Between 2016 and 2023, diet‐related deaths in Australia increased from 29,994 to 33,405 (+11%), while in New Zealand, they increased from 6411 to 6777 (+6%). Deaths attributable to excess red meat intake remained relatively stable in Australia (from 6347 to 6341 deaths) and declined modestly in New Zealand (from 1235 to 1187 deaths). In contrast, deaths attributable to refined grain intake increased substantially in both countries, more than doubling in Australia (74 to 158 deaths) and New Zealand (301 to 602 deaths). In Australia, deaths attributable to low vegetable intake also increased (1400 to 1853 deaths), whereas in New Zealand, they declined (218 to 179 deaths). These trends are consistent with ongoing shifts in dietary patterns towards higher consumption of ultra‐processed foods [109, 110]. Reducing greenhouse gas emissions from agricultural production (see Indicator 3.4) could have substantial health co‐benefits by reducing disease from excessive red meat consumption.

### 5.6 Healthcare Sector Emissions (Indicator 3.6)

The healthcare sector contributes a significant share of greenhouse gas emissions, driven by the energy and material inputs required to deliver its services [111]. This indicator calculates healthcare emissions in Australia and New Zealand from 2016 to 2023 (Appendix S1, 3.6). It combines World Health Organization (WHO) national health expenditure data with the EXIOBASE (https://exiobase.eu) environmentally extended input–output model to estimate greenhouse gas emissions associated with healthcare, covering both direct emissions from healthcare facilities and indirect emissions from the goods and services they use. Total healthcare emissions are estimated from expenditure and EXIOBASE emission coefficients, while per‐capita emissions use the national population as the denominator.

Australia shows a generally declining trend in total and per capita healthcare‐related greenhouse gas emissions, despite noticeable spikes around 2018 and a modest increase from 2022 to 2023 (Figure 11A,B). New Zealand exhibits lower total emissions, but higher per capita emissions, the latter markedly increasing in the early 2020s. Healthcare‐related disability‐adjusted life years (DALYs) in Australia decreased from a peak in 2018, indicating some improvement in public health damages from pollution caused by the health sector. New Zealand shows a fluctuating trend over time (Figure 11C,D).

**FIGURE 11:**
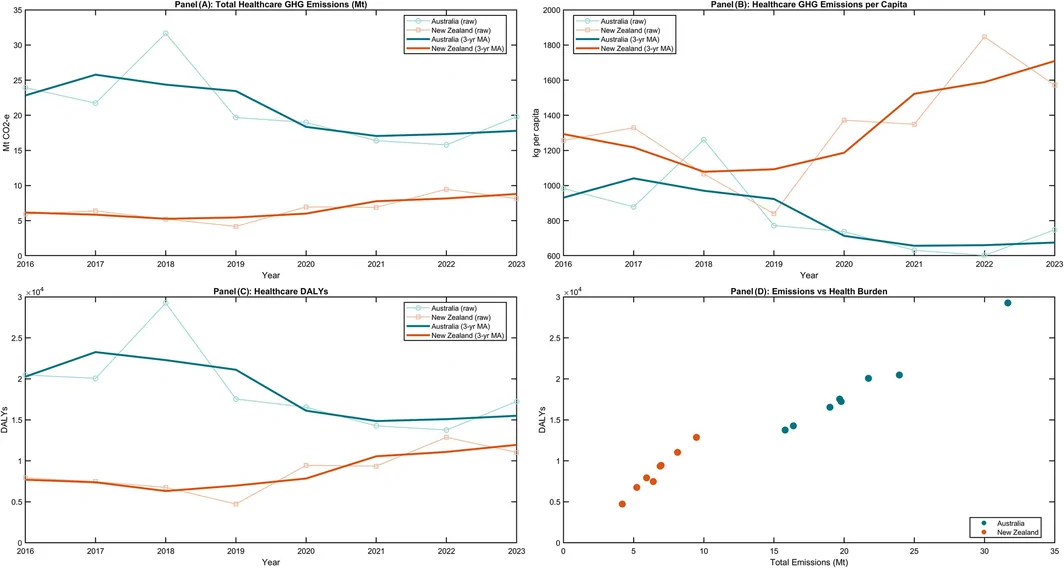
(A) Annual total healthcare‐related GHG emissions, (B) annual total healthcare‐related GHG emissions per capita, (C) associated DALYs^a^ and (D) scatterplot of annual total healthcare‐related GHG emissions and DALYs, for Australia and New Zealand from 2016 to 2023.^b^ GHG, greenhouse gas; DALYs, disability‐adjusted life years; PM_2.5_, fine particulate matter; O_3_, ozone; MA, moving average; Mt, megatonne; CO_2‐e_, carbon dioxide‐equivalent. ^a^Total public health damages due to PM_2.5_ and O_3_ pollution from the national healthcare sector are captured here. ^b^(A–C) Raw annual values are shown alongside smoothed trends using a 3‐year moving average to highlight underlying trends over time.

### 5.7 Summary

The indicators presented in this section point to mixed progress in aligning climate mitigation with health and equity objectives. Renewable energy generation, rooftop solar uptake and active transport options have continued to expand and show how emissions reduction opportunities can improve public health outcomes. At the same time, however, fossil fuel dependence, inequitable access to clean energy technologies, emissions‐intensive agricultural systems, worsening dietary trends and ongoing healthcare sector emissions remain significant challenges. These findings suggest that mitigation policies will deliver the greatest health benefits when they are designed alongside broader social, infrastructure and public health priorities. More integrated and inclusive approaches will be critical to achieving both emissions reductions and long‐term improvements in population health and well‐being. Box 3 provides an overview of Australian healthcare quality and safety standards for climate resilience and environmental sustainability.

BOX 3 **Australian Healthcare Quality and Safety Standards for Climate Resilience and Environmental Sustainability.**

**Table:** 

Health organisations face a bidirectional challenge of building health systems that are simultaneously climate resilient and environmentally sustainable [112]. Many Australian health organisations have declared an intent to meet this challenge (see Indicators 2.1 and 3.6; [113]). For example, the New South Wales Ministry of Health's Net Zero Roadmap commits to a ‘high‐quality, low‐carbon and climate resilient health system by focusing on quality, value, innovation and equity’ [114]. Central to implementing these ambitions is the overarching requirement to maintain quality and safety, underpinned by robust clinical governance systems. Health practitioners in this discipline provide leadership, not just for compliance, but for continuous improvement and health system transformation across hospitals, clinics and communities. Clinical governance teams can now position themselves at the forefront of the health response to climate change, with the launch of the Healthcare Sustainability and Resilience Module [115]. This module sets out new safety and quality standards for sustainability and resilience, integrated across eight National Safety and Quality Health Service (NSQHS) Standards for the accreditation of hospitals and healthcare services. Accreditation is a major undertaking whereby health organisations demonstrate efforts to protect the public from harm and improve the quality of health service provision. Accreditation can also drive clinical care standards and improve hospital operations—during business as usual and climate‐related disruptions. The new standards can produce a step change in redefining sustainability as a domain of healthcare quality, embedding the principles of sustainable clinical practice and reducing low value care [116]. Quality and safety practitioners are identifying how these new standards can be achieved through routine clinical governance, quality improvement projects, innovations in clinical practice and low‐carbon models of care. The International Society for Quality in Health Care (ISQua) Green Paper outlines 12 pathways for practical actions on environmental sustainability in health systems [117]. A joint statement between Australia's healthcare quality and safety regulator and medical colleges supports health organisations to develop low‐emissions models of care, including actions to improve care, minimise wasteful healthcare and involve patients in decisions about appropriate care; and mobilise and support the health workforce to lead the health system response to climate change [118]. Leading health organisations are incorporating sustainability concepts such as the Triple Bottom Line in their clinical governance frameworks [119]. Closer to the clinician and patient care interface, validated tools such as Sustainable Quality Improvement are available [120, 121]. Together, these approaches enable health organisations to lead clinical transformation efforts in a time of climate crisis.

## 6 Section 4: Economics and Finance

This section reports economic losses from climate‐related extreme events and examines financial and economic mechanisms relevant to protecting human health from the impacts of climate change and supporting the transition to a low‐carbon economy. We continue to examine inflation‐adjusted insured losses from disaster events as well as the increasing economic costs associated with heat‐related mortality. The section also focuses on recent developments related to the renewable energy transition and climate mitigation. Indicators track investment in clean energy generation and the net effect of fossil fuel subsidies and carbon pricing. For the first time, we include an indicator assessing country preparedness for the transition to net zero, presenting results for both Australia and New Zealand.

### 6.1 Economic Losses due to Climate‐Related Extreme Events (Indicator 4.1)

This indicator tracks the total insured economic losses from disaster events for Australia and New Zealand and is based on data provided by the Insurance Councils of Australia [122] and New Zealand [123] (Appendix S1, 4.1). Figure S17 presents inflation‐adjusted (base year 2019) cumulative annual insured losses for Australia, arising from bushfires, cyclones, flooding, hailstorms, storm flooding and tornados. For 2025, major losses were attributed to flooding as a result of severe tropical cyclone Alfred in February and March, hailstorms in South‐East Queensland in October and severe storm and hail activity in Queensland and New South Wales in November. Total annual insured losses from catastrophes in Australia were AU$585 million in 2024 (the lowest level since 2012) and AU$2.615 billion in 2025. For New Zealand, following the unprecedented and highly damaging floods in 2023, which resulted in cumulative insured losses of nearly NZ$4 billion, annual losses fell to NZ$63.5 million in 2024 and NZ$119.3 million in 2025.

### 6.2 Costs of Heat‐Related Mortality (Indicator 4.2)

Climate change is increasing heat‐related mortality and people aged over 65 are particularly vulnerable. This indicator estimates the costs of heat‐related mortality in Australia and New Zealand from 2000 to 2025. It uses ERA5 temperature data, Global Burden of Disease mortality rates and population data to estimate heat‐related deaths among adults aged 65 years and older, and converts these deaths into years of life lost using WHO life expectancy data. These years of life lost are monetised using the Organisation for Economic Co‐Operation and Development value of a statistical life‐year and expressed in constant 2025 United States dollars (Appendix S1, 4.2). In Australia, costs reached an average US$1.49 billion per year in 2021–2025, a 119% increase from the 2000 to 2004 average cost of US$680 million (Figure 12A). In New Zealand, costs reached an average US$229 million per year in 2021–2025, a 342% increase from the 2000 to 2004 average cost of US$52 million (Figure 12B). The 2025 costs were the second highest on record for each country, behind peaks in 2019 for Australia and 2022 for New Zealand.

**FIGURE 12:**
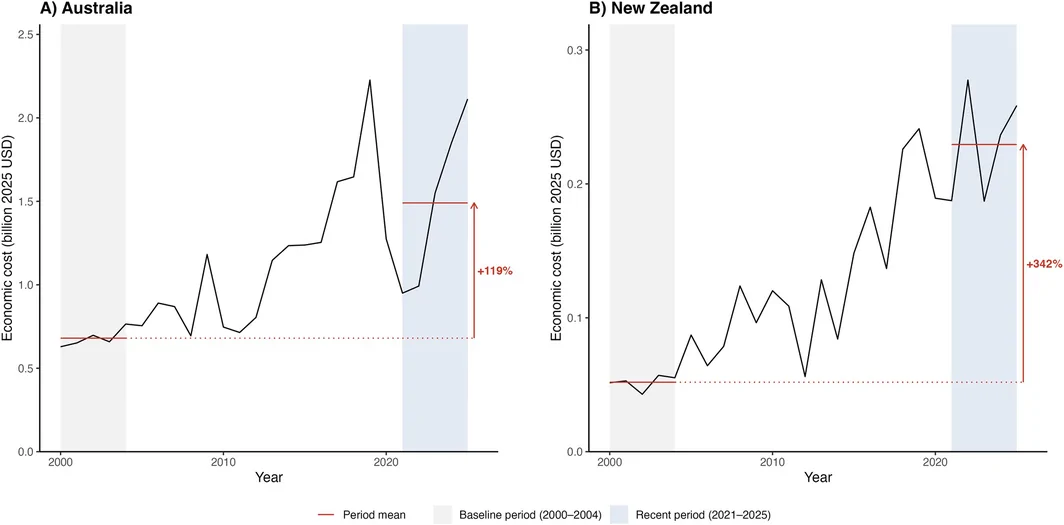
Costs of heat‐related mortality in 2025 United States dollars among people aged over 65 years in (A) Australia and (B) New Zealand. Average costs are reported for the baseline (2000–2004) and current (2021–2025) periods. The percentage change between periods is reported.

### 6.3 Country Preparedness for the Transition to Net Zero (Indicator 4.3)

Achieving a net zero greenhouse gas economy is critical for long‐term health outcomes, but it entails substantial economic and structural transformation. Although the transition offers important co‐benefits, it may also introduce risks. These risks are more pronounced in countries with carbon‐intensive industries, strong dependence on fossil fuels and workforces concentrated in high‐emission sectors, particularly where existing social inequalities are significant. This indicator evaluates Australia's and New Zealand's preparedness for the net zero transition from 2023 to 2025 (Appendix S1, 4.3). It combines 25 country‐level measures: 16 capturing resilience, such as economic stability, infrastructure, finance and governance; and 9 capturing vulnerability, such as fossil‐fuel dependence, carbon‐intensive industries and income inequality. The measures are normalised and weighted using principal component analysis, then combined and rescaled from 0 to 1 to estimate countries' resilience to transition risk, lack of vulnerability to transition risk and overall preparedness for the low‐carbon transition, with higher scores indicating better preparedness. As this is a composite index rather than a rate, there is no single population or economic denominator. Figure 13 illustrates that, in 2025, Australia achieved an overall score of 0.60 (down from 0.84 in 2023) and New Zealand a score of 0.61 (down from 0.81 in 2023), with the resilience of these two countries to transition risk in particular dropping substantially from 2023 to 2025.

**FIGURE 13:**
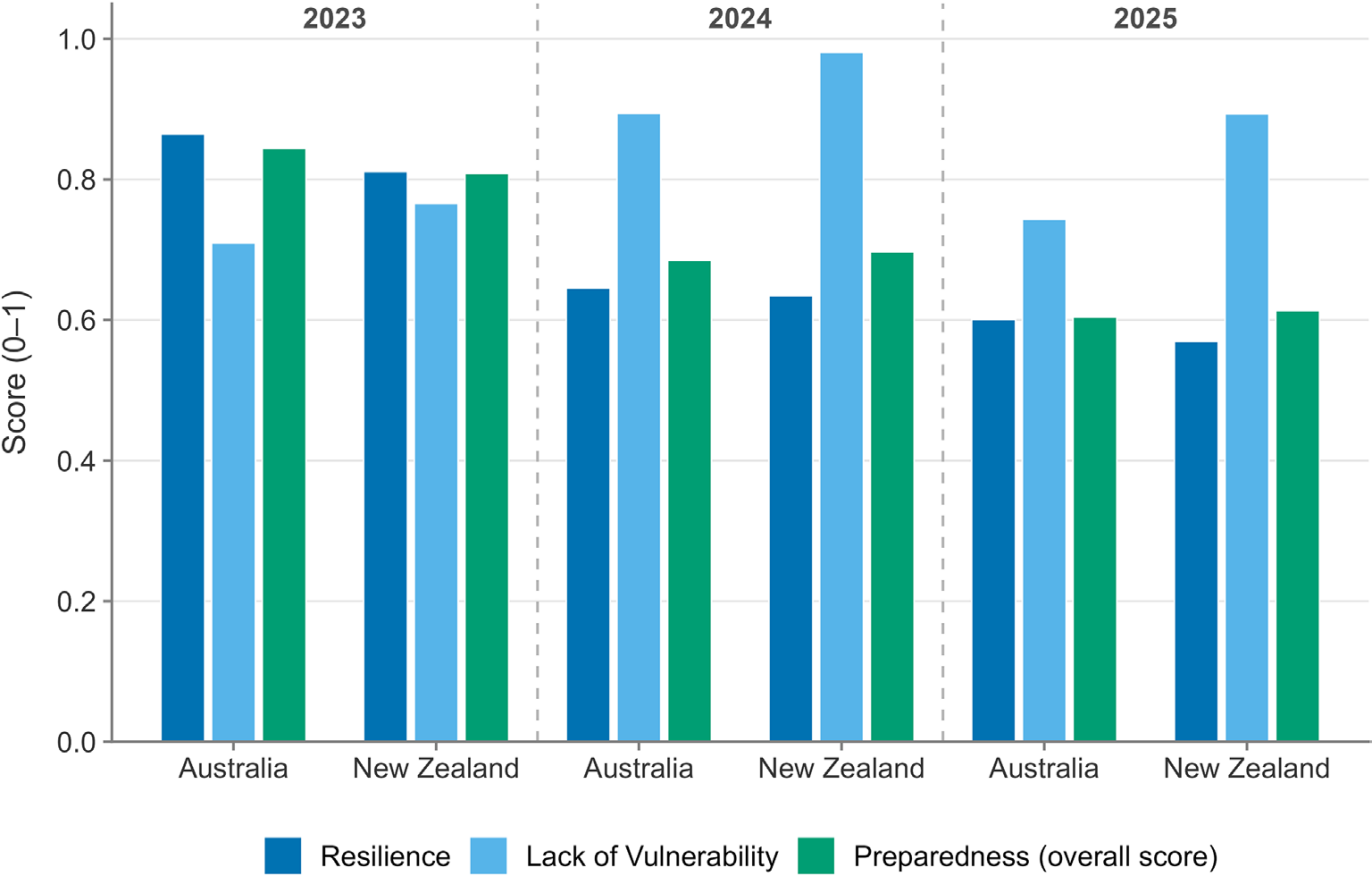
Australia's and New Zealand's preparedness for the transition to net zero, 2023–2025. Scores are presented for resilience, lack of vulnerability and overall preparedness. All scores range from 0 (lowest preparedness) to 1 (highest preparedness).

### 6.4 Clean Energy Investment (Indicator 4.4)

This indicator reports investment in renewable energy based on generation capacity in the Australian National Electricity Market (NEM) (Appendix S1, 4.4). According to the latest reports of the Australian Energy Regulator [124] and Australian Energy Market Operator [125], investment in renewable generation continues to increase, reflecting strong growth in large‐scale wind and solar as well as distributed rooftop installations. Over the two financial years 2023–2024 and 2024–2025, about 2900 megawatts (MW) of wind and 1850 MW of solar generation capacity were added (Figure 14). During this period, additional battery capacity (over 1900 MW) and some gas‐fired generation (320 MW) were also introduced to support peak demand and ensure system reliability during periods of high demand and low renewable output. Notably, over the same period, no further coal‐fired generation capacity has been withdrawn, while current projections do not indicate any new investment in coal‐fired generation.

**FIGURE 14:**
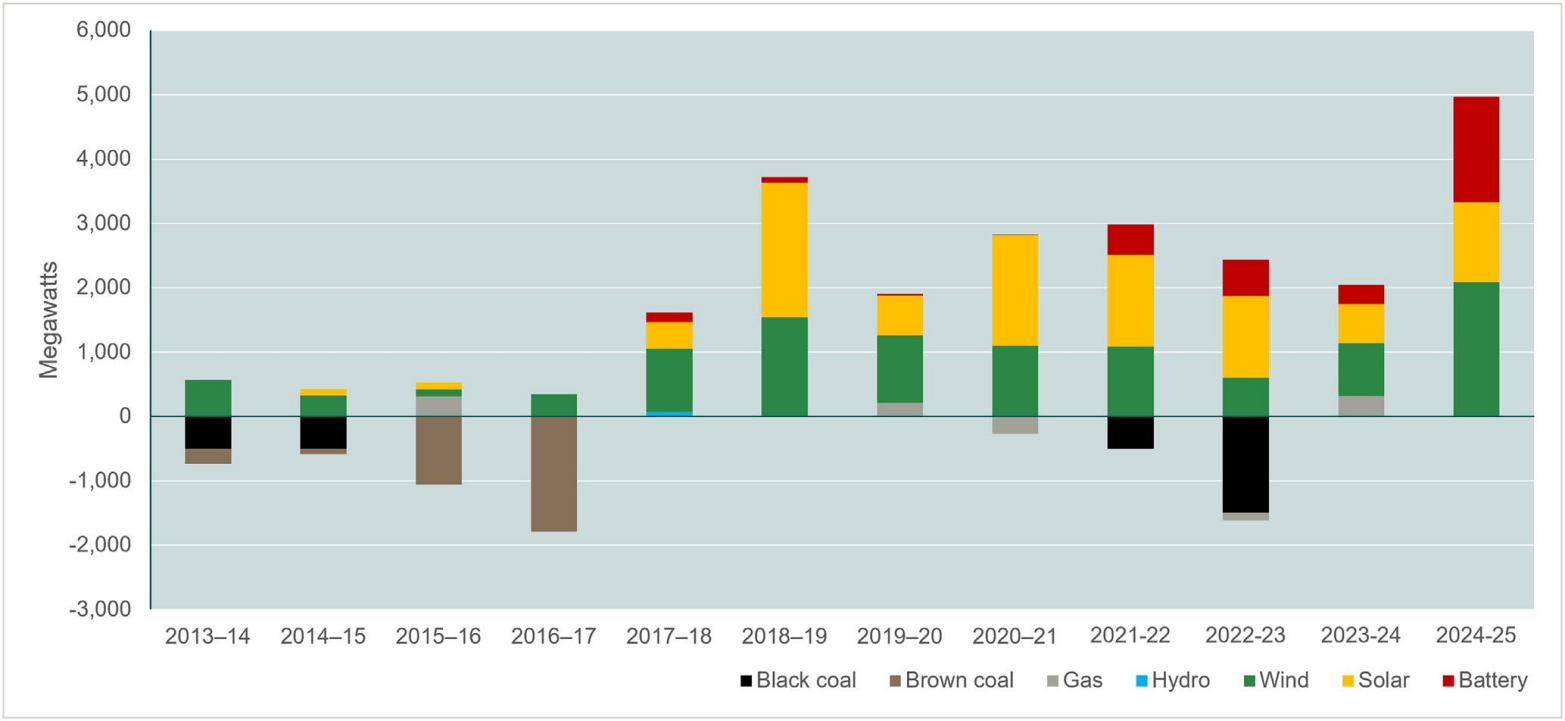
New investment and capacity withdrawal in the Australian National Electricity Market for different fuel sources (black coal, brown coal, gas, hydro, wind, solar and battery) for the financial years 2013–2014 to 2024–2025. Data sources: Australian Energy Regulator (AER) [124] and Australian Energy Market Operator (AEMO) [125].

### 6.5 Net Value of Fossil Fuel Subsidies and Carbon Prices (Indicator 4.5)

The phasing out of fossil fuels is urgently required to reduce the health impacts of climate change, with carbon pricing working for this endeavour and fossil fuel subsidies working against it. This indicator reports the net value of fossil fuel subsidies and carbon prices in Australia (Appendix S1, 4.5; Figure S18).

In 2024, Australia's net carbon price (the carbon price less the subsidy) was ‐AU$27.8 per tonne of CO_2_. This represents a substantial ongoing net fossil fuel subsidy. Australia still does not currently have an explicit carbon price, relying on the recently deployed Safeguard Mechanism that requires large carbon emitters, which are defined as facilities that emit more than 100,000 t of CO_2_ per year, to offset excess emissions above an assigned baseline by purchasing and surrendering Safeguard Mechanism Credits or Australian Carbon Credit Units [126].

### 6.6 Summary

The indicators in this section show that climate change continues to impose substantial economic and financial costs across Australia and New Zealand. Although insured losses from climate‐related disasters declined in 2024 compared with recent peak years, major extreme weather events continue to cause considerable economic damage in both countries. The estimated costs associated with heat‐related mortality have also increased substantially over recent decades. At the same time, investment in renewable energy generation and storage continues to increase, supporting progress towards a lower‐carbon electricity system. However, Australia continues to provide considerable net fossil fuel subsidies. The new indicator on preparedness for the transition to net zero emissions suggests that both Australia and New Zealand have gone backwards from 2023 to 2025, with their resilience to transition risk in particular dropping substantially.

## 7 Section 5: Public and Political Engagement

Active public and political engagement are a precondition for action on health and climate change. Engagement is necessary to share understanding of health impacts of climate change and of the need for urgent climate change mitigation and adaptation across all societal sectors to safeguard human health. The indicators reported in this section include the tracking and analysis of scientific publications on health and climate change by Australian and New Zealand authors. This section also reports on Australian media coverage, parliamentary engagement, research funding and litigation in relation to health and climate change.

### 7.1 Media Coverage of Health and Climate Change (Indicator 5.1)

The media plays a critical role in engaging the public on climate and health issues and shaping policy debates. This indicator tracks coverage in major Australian newspapers and Australian Broadcasting Corporation (ABC) news and transcripts from 2008 to 2025 using the Factiva global news database (Appendix S1, 5.1). A total of 2661 relevant articles were identified over the period. Newspaper coverage of health and climate change peaked in 2022, followed by a 60% decline to 124 articles in 2025 (Figure 15). ABC news and transcripts also showed a 50% decrease from 2022 to 2025 (Appendix S1, Figure S19). Trends varied across states, with relatively stable coverage in Hobart (Tasmania) and Darwin (Northern Territory), but notable declines in national reporting and across other states, particularly in Western Australia, where climate and health issues have received minimal attention in recent years. No consistent seasonal pattern was observed; instead, coverage tended to align with extreme weather events, the release of academic reports, government policy announcements and international meetings.

**FIGURE 15:**
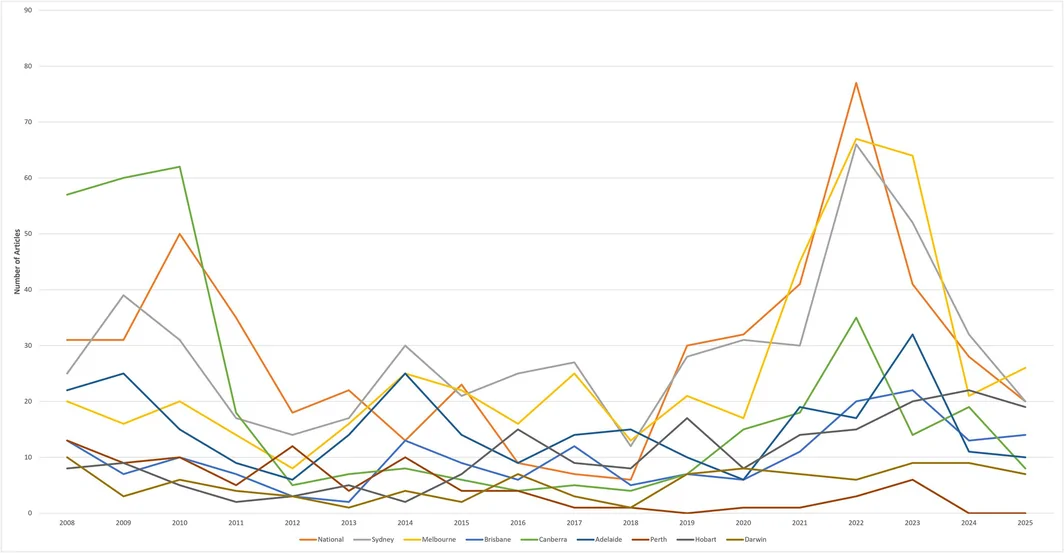
Annual number of newspaper articles on health and climate change by Australian major cities and country level, 2008–2025. Data source: Factiva [127].

### 7.2 Scientific Articles on Health and Climate Change (Indicator 5.2)

This indicator tracks scientific publications on health and climate change in Australia and New Zealand (Appendix S1, 5.2). We adopted the methods and country‐level data (by author affiliation) provided by the global *Lancet* Countdown [31]. As shown in Figure S20, the number of articles published by Australian researchers increased eightfold, from 38 in 2006 to 309 in 2023, before declining by 38% to 192 in 2025, which was similar to the 2016 level (171 articles). Of the 3267 relevant articles identified, 86% focused on health impacts, 11% on adaptation and 3% on mitigation. In New Zealand, a smaller body of work was identified (349 articles), with output increasing over time. Of these, 75% addressed health impacts, while 11% and 14% focused on adaptation and mitigation, respectively.

### 7.3 Scientific Engagement on the Health Impacts of Climate Change (Indicator 5.3)

This indicator builds on the dataset used for Indicator 5.2 to further analyse scientific publications on the health impacts of climate change (Appendix S1, 5.3). It tracks studies examining health outcomes associated with temperature and rainfall attributable to climate change between 2006 and 2025, using methods developed by the global *Lancet* Countdown [31]. In Australia, 1885 relevant attributable publications were identified, of which 91% (1720) addressed climate drivers and 71% (1344) were published from 2016 onwards. In New Zealand, 162 publications were identified over the same period, with annual outputs ranging from 0 to 18; 70% (113) were published since 2016 and 93% (151) examined climate drivers. The most frequently studied health outcomes in Australia and New Zealand were infectious diseases (29% and 29%, respectively), general mortality and morbidity (17% and 16%, respectively), respiratory diseases (16% and 13%, respectively), direct injuries (7% and 11%, respectively) and mental health and sentiment (8% and 7%, respectively).

### 7.4 Government Engagement in Health and Climate Change (Indicator 5.4)

The extent of government engagement on health and climate change signals how highly decision‐makers prioritise the issue. This indicator reports on government engagement by tracking bills, legislation, committees and inquiries in the parliaments of the Commonwealth of Australia and all eight states and territories (Appendix S1, 5.4). Consistent with previous years, the Parliament of Australia showed some engagement in 2024 and 2025 on the issues of climate change and health; however, they remain largely siloed (Table S5). The exception was the Climate Change Amendment (Duty of Care and Intergenerational Climate Equity) Bill 2025, which was re‐introduced by independent Senator David Pocock (an updated version of the one previously introduced in 2023) [128]. The Bill sought to amend the *Climate Change Act 2022* to ‘require decision‐makers to consider the health and well‐being of children in Australia when making significant decisions’ but did not pass into law. In addition, there was an inquiry into the impact of microplastics and other toxics on human health [129]. There was more engagement on climate health in the state and territory parliaments, primarily through parliamentary inquiry but also Bills put forward by more left‐leaning governments. Twenty‐four Bills were introduced and 40 Inquiries were held across the jurisdictions on topics discussing climate health issues (Appendix S1, Tables S6 and S7).

### 7.5 Health and Climate Change Research Funding (Indicator 5.5)

Research on health and climate change in Australia is fundamental to Australia's understanding of and response to this critical issue [130]. This indicator tracks the annual number of funded and not‐funded National Health and Medical Research Council (NHMRC) health and climate change grant applications from 2000 to 2025 (Appendix S1, 5.5), adding 2 further years (2024 and 2025) since we last reported on this indicator and, for the first time, benchmarking the success rate of health and climate change grant applications against the overall NHMRC success rate (Figure 16). Health and climate change grant applications surged in 2024 and 2025, with a record 87 applications in 2024. The success rate also jumped in these 2 years compared with the previous 2 years (2022 and 2023) and was just above the overall NHMRC success rate, resulting in a record 13 grants funded in 2024.

**FIGURE 16:**
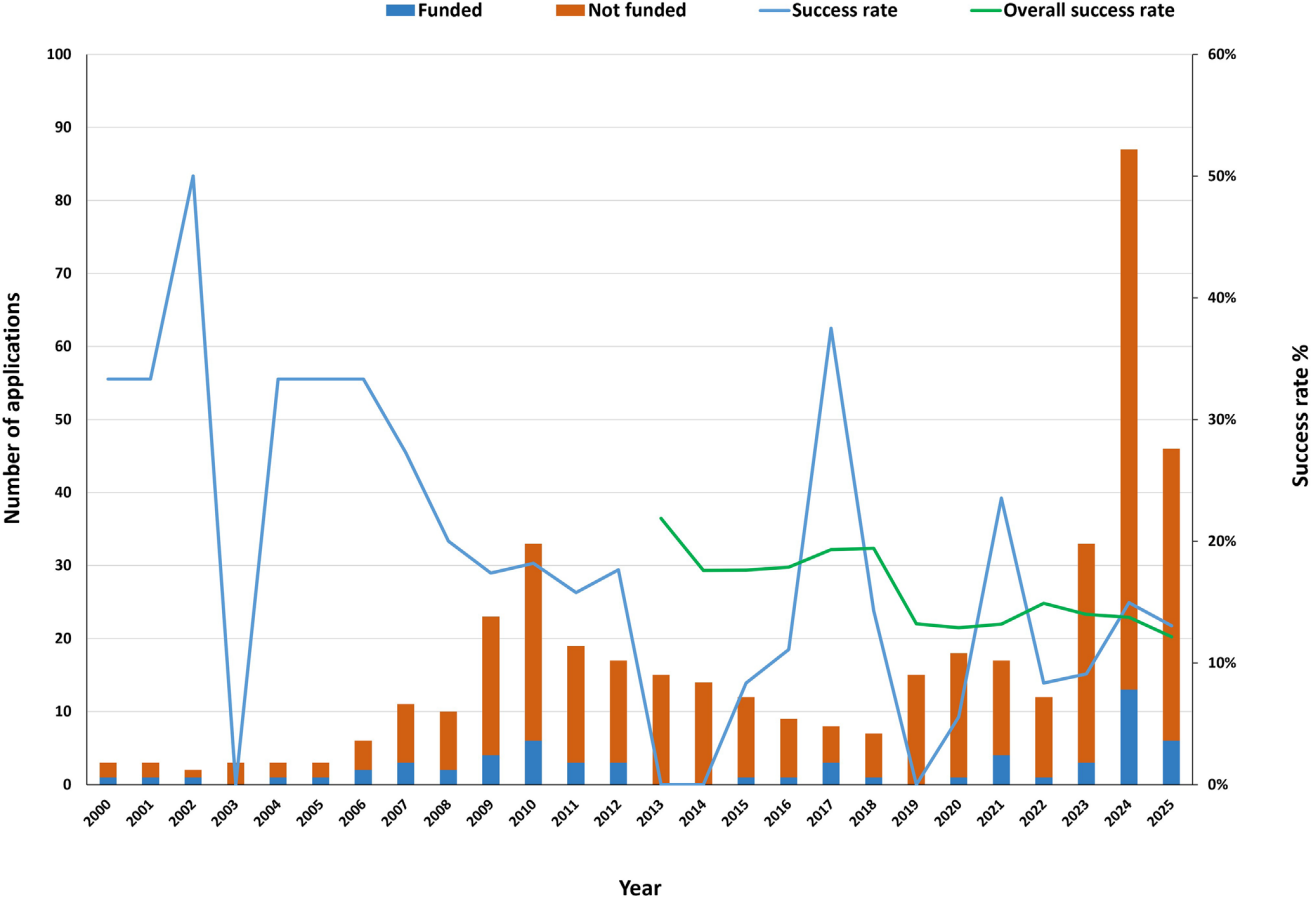
Annual number of funded and not‐funded National Health and Medical Research Council (NHMRC) grant applications with a focus on health and climate change, and success rate, 2000–2025. Also shown is the overall annual NHMRC success rate, 2013–2025. Note that not all rounds from 2025 application year had been announced by the date the search was conducted (12 February 2026). Data source: Australian Government [131].

### 7.6 Health and Climate Change Litigation (Indicator 5.6)

Litigation has emerged as a tool for individuals, civil society and governments to challenge inadequate action on climate change and health by both public institutions and private entities [132, 133]. This indicator quantifies, tracks and characterises health and climate change‐related litigation in Australia from 2014 to 2025. The methodology was refined this year to also include a chronological search of all recent cases filed and an assessment if the relevant judgements were positive, negative or neutral with respect to action on climate change (Appendix S1, 5.6). Health featured in 13 Australian climate cases from 2024 to 2025. Some cases (e.g., some planning decisions, costs) were considered to be neutral for climate action. Of the 13 cases, 4 related to coal mines, 1 related to a gas project, 1 related to a duty of care and 7 cases related to planning. The planning cases were connected to climate change through the potential for increased risks associated with flooding and associated threat to life. These cases are important examples of how everyday decisions around planning and lives are being impacted by climate risks.

### 7.7 Summary

Media coverage of health and climate change has declined in most Australian jurisdictions from a peak in 2022, while remaining relatively stable in Tasmania and the Northern Territory. The number of scientific articles on health and climate change published by Australian and New Zealand authors has declined from a peak in 2023. The most frequently studied health outcomes were infectious diseases, general mortality and morbidity and respiratory diseases. The number of health and climate change applications to the NHMRC surged in 2024 and 2025, with a success rate comparable to the overall NHMRC success rate. In Australia, there was more engagement on climate change and health in the state and territory parliaments than the national parliament, with a total of 24 Bills introduced and 40 Inquiries addressing climate change and health issues across the jurisdictions. Health featured in 13 climate change legal cases in Australia during 2024–2025.

## 8 Conclusion: The *MJA* –*Lancet* Countdown Oceania in 2026

Our analyses show that exposure of infants and older adults to heatwaves in Australia and New Zealand has increased substantially over the past decade due to climate change (Figure 17), and half of the 16,230 young Australians aged 14–19 years surveyed reported that they are worried or sad about the impacts of climate change (including its threat to people) and about 1 in 7 reported that such feelings are negatively affecting their daily life. Increasing heat‐related mortality alone is placing a large economic burden on Australians, with associated inflation‐adjusted costs over the period 2021–2025 more than double what they were at the start of the century, now averaging an estimated US$1.49 billion per year nationally.

**FIGURE 17:**
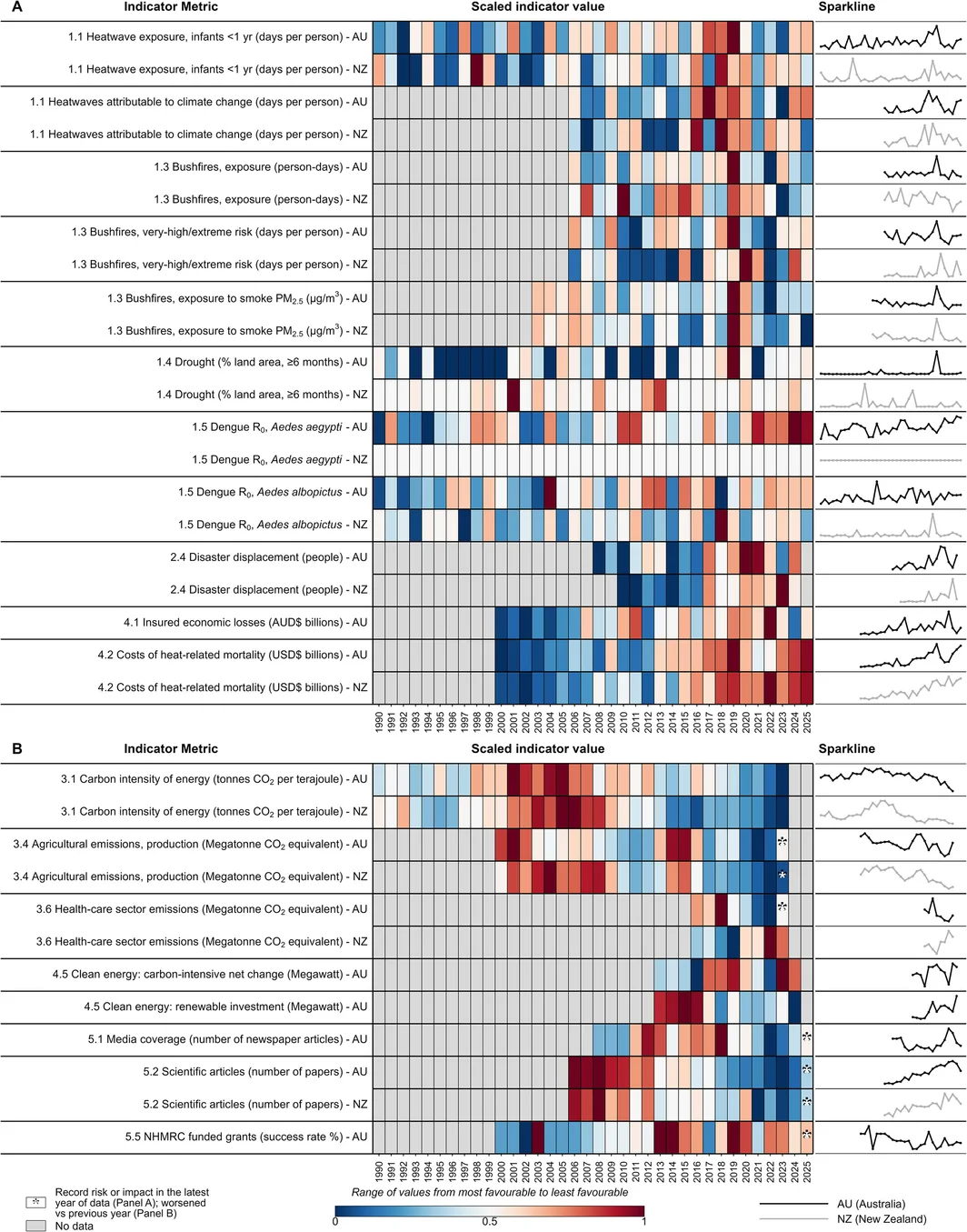
Summary of indicators for which quantitative data per year are available, from 1990 to 2025.^a^ PM, particulate matter; CO_2_, carbon dioxide. ^a^The heatmaps present the time series for each indicator, with values linearly scaled into the range 0–1, such that 0 and 1 represent the minimum or maximum values in the time series shown, and 0.5 represents the median. The sparklines present a line graph of the scaled indicator. (A) health hazard, exposure and impact indicators; and (B) indicators reflecting responses to climate change.

In other areas, some progress is being made. New Zealand's Health National Adaptation Plan 2024–2027, published in October 2024, lays the foundation for building climate resilience within its health system, while acknowledging the environmental and social determinants of health that apply more broadly. The provision of climate services to the health sector in both Australia and New Zealand is categorised as advanced. In Australia, healthcare‐related greenhouse gas emissions generally declined from 2016 to 2023.

In conclusion, the threat to physical and mental health from climate change continues to grow. Despite pockets of progress, Australia and New Zealand have a long way to go to adequately mitigate and adapt to climate change, and in so doing not only avoid health impacts but seize the opportunity for generational health gains. Important opportunities have been lost, such as the failed attempts to amend Australia's *Climate Change Act 2022* to ‘require decision‐makers to consider the health and well‐being of children in Australia when making significant decisions’. In 2026, Australia is in the spotlight as President of Negotiations for the United Nations Climate Change Conference (COP31)—an unprecedented opportunity for the Australian Government to push through the sizable step change in global climate action that is now critically required. Australia's role as one of the world's largest exporters of coal and liquefied natural gas means that its contribution to global greenhouse gas emissions substantially exceeds its domestic footprint [134, 135], a tension that sits at the heart of its responsibilities as COP31 President of Negotiations. Human health must be central to all climate change negotiations, with the evidence now clear and plentiful of the impacts not only in Australia and New Zealand but also among our Pacific island country neighbours [22, 136, 137].

## Author Contributions

Paul J. Beggs is the Director of the Lancet Countdown Oceania Regional Centre and leads the *MJA–Lancet* Countdown and contributed to the conceptualization of the manuscript. The following authors contributed to the writing (original draft) of the section introductions and summaries: Alistair J. Woodward (Section 1), Hilary Bambrick (Section 2), Martina K. Linnenluecke (Section 3), Stefan Trueck (Section 4) and Anthony G. Capon (Section 5). The following authors contributed to the writing (original draft) of the Indicators: Alistair J. Woodward (indicator 2.1), Arunima Malik (indicator 3.6), Bronwyn Wilkes (indicator 2.5), Celia McMichael (indicator 2.4), Christopher D. McKay (indicator 2.5), Donna Green (indicator 4.5), Harry R. Kennard (indicator 3.4), Ivan C. Hanigan (indicator 1.4), Jacqueline Peel (indicator 5.6), James Smallcombe (indicator 1.2), Jake T.W. Williams (indicator 4.2), Kathryn Bowen (indicator 5.6), Kerry Nice (indicator 3.3), Katherine Sievert (indicator 3.5), Maddie Heenan (indicator 5.4), Martina K. Linnenluecke (indicators 3.1 and 3.2), Mark Stevenson (indicator 3.3), Nicolas Borchers Arriagada (indicator 1.3), Ollie Jay (indicator 1.2), Paul J. Beggs (indicators 1.5, 2.2, 2.3 and 5.5), Raymond Lovett (indicator 2.5), Rebekkah Markey‐Towler (indicator 5.6), Sachith Seneviratne (indicator 3.3), Stefan Trueck (indicators 4.1, 4.3 and 4.4), Sotiris Vardoulakis (indicator 2.1), Tara Crandon (indicator 1.6), Thanh Ho (indicator 3.3), Troy J. Cross (indicator 1.2), Tanya Singh (indicators 1.1 and 5.5), Ying Zhang (indicators 5.1, 5.2 and 5.3) and Zerina Lokmic‐Tomkins (indicator 1.1). Fiona Langridge wrote (original draft) the box on Child health and climate change in Oceania (Box 1), Veronica Matthews and Lynette Dimer wrote (original draft) the box on First Nations strengths in the face of climate change (Box 2) and Aditya Vyas wrote (original draft) the box on Australian healthcare quality and safety standards for climate resilience and environmental sustainability (Box 3). Muhammad M. Hasan provided the coordination of data sharing through the *Lancet* Countdown. Calla MacGregor, Philomena Colagiuri and Tanya Singh contributed to the background research. Paul J. Beggs wrote (original draft) the manuscript and all authors contributed to reviewing it critically for important intellectual content. All authors provided final approval of the version to be published and agreement to be accountable for all aspects of the work. Paul J. Beggs, Marina B. Romanello and Tanya Singh made substantial contributions to the overall conceptualization and design of the work.

## Funding

This work was supported by the Wellcome Trust (304972/Z/23/Z).

## Disclosure

Not commissioned; externally peer reviewed.

## Conflicts of Interest

The authors declare no conflicts of interest, except A.G.C. who declares that he chairs the WHO Technical Advisory Group on Climate Change, the Environment and Health in the Western Pacific Region; and H.B. declares that she is Director of the National Centre for Epidemiology and Population Health, The Australian National University, on the Board of Directors and Research Committee Chair of The Australia Institute, on the Climate and Health Expert Advisory Group of the Australian Centre for Disease Control, and on the Expert Advisory Committee of the National Climate Risk Assessment.

## Supporting information


**Appendix S1:** mja270315‐sup‐0001‐supinfo.pdf.

## Data Availability

Some of the study data are publicly available, as indicated in the article. Other study data can be accessed by contacting the corresponding author.
