# Supplementary material for: The 2026 Oceania Report of the MJA–Lancet Countdown on Health and Climate Change: Children Already Impacted, With a Lifetime of Exposure to Come
Source: Med J Aust. 2026 Oct 28;224(11):e70315. doi: 10.5694/mja2.70315 (PMC13715459; doi:10.5694/mja2.70315)

Supporting Information

Supplementary material

This appendix was part of the submitted manuscript and has been peer reviewed. It is posted as supplied by the authors.

Appendix to: P. J. Beggs, T. Singh, H. Bambrick, et al. The 2026 Oceania Report of the *MJA–Lancet* Countdown on Health and Climate Change: Children Already Impacted, With a Lifetime of Exposure to Come. *Medical Journal of Australia* 2026; doi: 10.5694/mja2.70315.

Appendix S1: The 2026 Oceania report of the *MJA-Lancet* Countdown on health and climate change: children already impacted, with a lifetime of exposure to come

This Appendix includes details of the data, methods, and caveats for each of the 28 indicators assessed in the 2026 *MJA-Lancet* Countdown. It also includes additional figures and tables that further illustrate some of the indicators. Finally, it includes, for several indicators, current thoughts regarding the potential future form of the indicator. This is provided in the context of this being the eighth *MJA-Lancet* Countdown annual report and the acknowledgement that its indicators, like those of the *Lancet* Countdown, will likely continue to develop in future annual assessments.

Section 1: Health hazards, exposures, and impacts

1.1 Exposure of vulnerable populations to heatwaves

Data

Data for this indicator are the same as those used for "Indicator 1.1.1: exposure of vulnerable populations to heatwaves" in Romanello et al. [1].

Methods

Methods for this indicator are the same as those used for "Indicator 1.1.1: exposure of vulnerable populations to heatwaves" in Romanello et al. [1].

Caveats

Caveats for this indicator are the same as those used for "Indicator 1.1.1: exposure of vulnerable populations to heatwaves" in Romanello et al. [1].

Future form of the indicator

Future modifications for this indicator are the same as those used for "Indicator 1.1.1: exposure of vulnerable populations to heatwaves" in Romanello et al. [1].

1.2 Heat and physical activity

Data

Data for this indicator are the same as those used for "Indicator 1.1.2: heat and physical activity" in Romanello et al. [1].

Methods

Methods for this indicator are the same as those used for "Indicator 1.1.2: heat and physical activity" in Romanello et al. [1].

Caveats

Caveats for this indicator are the same as those used for "Indicator 1.1.2: heat and physical activity" in Romanello et al. [1].

Future form of the indicator

Future modifications for this indicator are the same as those used for "Indicator 1.1.2: heat and physical activity" in Romanello et al. [1].

1.3 Bushfires

Data

Data for this indicator are the same as those used for "Indicator 1.2.1: Wildfires" in Romanello et al. [1].

Methods

Methods for this indicator are the same as those used for "Indicator 1.2.1: Wildfires" in Romanello et al. [1].

Caveats

Caveats for this indicator are the same as those used for "Indicator 1.2.1: Wildfires" in Romanello et al. [1].

Future form of the indicator

In future iterations, this indicator could be extended to provide results at a sub national scale. Furthermore, the bushfire exposure indicator could also be enhanced by incorporating population density, allowing results to be more comparable across time and geographic locations. Finally, the bushfire risk indicator could be refined by accounting for land cover types with higher ignition potential and fuel (i.e., biomass) availability. For example, highly arid environments such as deserts may exhibit elevated fire danger indices yet lack sufficient biomass to sustain bushfire ignition and spread.

1.4 Drought

Data

Data for this indicator are the same as those used for "Indicator 1.2.2 Drought" in Romanello et al. [1].

Methods

Methods for this indicator are the same as those used for "Indicator 1.2.2 Drought" in Romanello et al. [1].

Caveats

Caveats for this indicator are the same as those for "Indicator 1.2.2 Drought" in Romanello et al. [1].

Future form of the indicator

Future modifications for this indicator are the same as those for "Indicator 1.2.2 Drought" in Romanello et al. [1].

1.5 Climate suitability for infectious disease transmission

Data

Data for this indicator are the same as those used for "Indicator 1.3.1: Climate suitability for infectious disease transmission - Dengue" in Romanello et al. [1].

Methods

Methods for this indicator are the same as those used for "Indicator 1.3.1: Climate suitability for infectious disease transmission - Dengue" in Romanello et al. [1].

Caveats

Caveats for this indicator are the same as those for "Indicator 1.3.1: Climate suitability for infectious disease transmission - Dengue" in Romanello et al. [1].

Future form of the indicator

The future form of this indicator is the same as that for "Indicator 1.3.1: Climate suitability for infectious disease transmission - Dengue" in Romanello et al. [1].

1.6 Mental health and climate change

Data

Data for this indicator were obtained from Mission Australia's 2025 Youth Survey, wherein results relevant to climate change were sourced, with permission, from a report developed by Orygen and Mission Australia [2].

Methods

The Mission Australia Youth Survey is an annual, national survey of Australian youth aged 14-19 years. For the survey conducted in 2025, data collection occurred between the 10th of April to the 20th of August. Recruitment was conducted in schools, and through local community and service organisations. Young people were invited to take part in the survey through three predominant means: (1) via email; (2) through social media advertisements; and (3) by receiving the link through networks such as local organisations. A total of 17,155 young people completed the survey in 2025, of which 16,230 young people completed all questions on climate change. Further information on the 2025 survey and results can be found in the national report developed by Mission Australia [3].

Caveats

Given that the Mission Australia Youth Survey does not predominantly focus on climate change, the data available on how youth are impacted remain limited. Feelings about climate change will oscillate in salience, valence (pleasant to unpleasant emotions), arousal, duration as well as intensity. Though this indicator presents data on how worried and sad young people feel about climate change, there could be additional emotional experiences and mental health impacts at play. Furthermore, a plethora of socio-ecological factors shape how youth feel about and cope with climate change, and these influences could not be captured or analysed. The data presented in this report could be used as a baseline indicator for which to compare how youth feel about climate change over time [4].

Future form of the indicator

Though this indicator represents a promising start to understanding how young people feel about climate change, it is still in early development. The continuation of a mental health indicator such as youth emotions about climate change is the key priority. In the coming years, future indicators on mental health could capture a broader range of mental health

outcomes, including other emotional states outside of worry or sadness, as well as coping mechanisms, subjective well-being and mental health disorders.

Section 2: Adaptation, planning, and resilience for health

2.1 Health adaptation plans and assessments

Data

Data for this indicator are largely the same as those used for "Indicator 2.1 Health adaptation plans and assessments" in Beggs et al. [5].

Methods

Methods for this indicator are the same as those used for "Indicator 2.1 Health adaptation plans and assessments" in Beggs et al. [5].

Caveats

Caveats for this indicator are the same as those for "Indicator 2.1 Health adaptation plans and assessments" in Beggs et al. [5].

Future form of the indicator

Future versions of this indicator will focus on the National Adaptation Plan and Health National Adaptation Plan, as well as state and territory adaptation and health adaptation plans.

2.2 Climate information for health

Data

This indicator uses data for Australia and New Zealand in 2019 and 2024 from the World Meteorological Organization's (WMO's) Climate Services Dashboard [6]. This provides self-reported data on climate services capacities by National Meteorological and Hydrological Services (NMHSs) - with these being the Australian Government Bureau of Meteorology and New Zealand's MetService. This includes the overall climate services capacity level, categorised as: less than basic, basic, essential, full, or advanced. The provision or not of climate services to the health sector is also included across the six services assessed: data services, climate monitoring, climate analysis and diagnostics, climate projections, climate change projections, and tailored products.

Methods

This indicator uses data from the WMO's Climate Services Dashboard to track the provision of climate services to the health sector in Australia and New Zealand in 2019 and 2024.

Caveats

The data from WMO used for this indicator only consider climate services provided by NMHSs. It is unclear the degree to which other providers are being used.

Future form of the indicator

There are currently no plans to further develop this indicator.

2.3 Detection, preparedness, and response to health emergencies

Data

Data for this indicator for Australia and New Zealand for the years 2021-2024 are obtained from the World Health Organization International Health Regulations Capacity Progress website [7]. The annual implementation status, expressed as a percentage, of the Health Emergency Management capacity (C7) and the Zoonotic Diseases capacity (C12) was obtained for both countries, along with the respective global and World Health Organization Western Pacific Region average implementation statuses, which serve as useful benchmarks. Data were obtained on 19 February 2026. Description of the International Health Regulations capacities can be found in World Health Organization [8].

Methods

The International Health Regulations (2005) [9] (IHRs) are an instrument of international law designed to prevent, protect against, control and provide a public health response to potential public health emergencies and the international spread of infectious diseases. With weather and climate causing or contributing to many public health emergencies and the spread of many infectious diseases, the IHRs serve as an important indicator of national climate change and health adaptive capacity. Countries report to the World Health Assembly on the implementation of the IHRs across 15 capacities. The score for each capacity is expressed as a percentage (0–100%), with 100% indicating full implementation, such as policies and strategies to support and facilitate the development and implementation of the capacity are in place at the national, intermediate and local levels by all relevant sectors and are revised and updated on a regular basis [8]. The indicator tracks the annual implementation status in

Australia and New Zealand over the period 2021-2024 of two capacities: the Health Emergency Management capacity (C7) and the Zoonotic Diseases capacity (C12). These are benchmarked against the respective global and regional average implementation statuses (Figure S2).

Caveats

Several limitations to this indicator are identified by Romanello et al. [10]. These include:

- the IHR capacity implementation status is self-reported by countries, which may introduce bias or subjectivity;
- the countries that report IHR implementation differ to some extent from year to year, which may lead to increases or decreases in global and regional average implementation statuses that do not reflect actual changes;
- IHR capacities are not specific to climate change, meaning they do not directly or specifically measure a country's adaptive capacity in relation to climate driven risk changes;
- the indicator captures potential capacity – not action.

Future form of the indicator

Future forms of this indicator will need to evolve along with any relevant changes to the International Health Regulations and associated capacity reporting and assessment.

2.4 Migration and displacement

Data

This indicator comprises two components: (a) population exposure to sea level rise of one metre, and (b) weather-related disaster displacement.

Data for population exposure to a global mean sea level rise of one metre for Australia and New Zealand are consistent with those used for Indicator 2.3.3 (Rising sea levels, migration, and displacement) in Romanello et al. [1].

Data on internal displacement caused by weather-related disasters in Australia (2008–2024) and New Zealand (2010–2024) are obtained from the Internal Displacement Monitoring Centre (IDMC) disaster displacement database [11]. The IDMC records the number of

internal displacements triggered by individual disaster events, disaggregated by hazard category (weather-related or geophysical) and hazard type. National population data used to calculate displacement rates are obtained from the World Bank's World Development Indicators [12, 13]. Both data series were extracted in March 2026. The IDMC data for New Zealand begin in 2010, as earlier years contain only geophysical (earthquake) events.

Methods

Methods for population exposure to a global mean sea level rise of one metre for Australia and New Zealand are consistent with those used for Indicator 2.3.3 (Rising sea levels, migration, and displacement) in Romanello et al. [1].

For the weather-related displacement component, all events classified under the 'Weather related' hazard category are extracted from the IDMC disaster displacement database for Australia and New Zealand. Events classified as 'Geophysical' (e.g., earthquakes, volcanic activity) are excluded. For each country, the total number of people displaced by weather-related events is summed for each calendar year. This annual displacement total is then divided by the corresponding mid-year national population estimate (from the World Bank's World Development Indicators) and multiplied by 100,000 to express the result as a displacement rate per 100,000 population. Weather-related hazard types captured include floods, storms (including tropical cyclones and tornadoes), bushfires, weather-related mass movements (landslides), and erosion.

Caveats

Caveats for the sea level rise component are the same as those described for Indicator 2.3.3 (Rising sea levels, migration, and displacement) in Romanello et al. [1].

The IDMC cannot be understood to represent displacement attributable solely to anthropogenic climate change; climate change operates as a risk amplifier for weather-related disasters. Importantly, since the 1970s there has been a greater increase in the number of weather-related disasters (e.g., floods) than non-weather-related disasters (e.g., earthquakes), suggesting that the upward trajectory in recorded weather-related disasters reflects a real trend.

Both the IDMC Global Internal Displacement Database and the World Bank's World Development Indicators are revised on an ongoing basis as new event reports, updated damage assessments and revised population estimates become available. In this analysis, each year's value is extracted once at the time of the corresponding *MJA-Lancet* Countdown annual report and is then carried forward unchanged in subsequent reporting, so retrospective revisions made to earlier years in the source databases are not reflected in the time series shown.

A separate methodological consideration is that IDMC counts the number of new internal displacements triggered by individual disaster events rather than unique individuals, so a person displaced multiple times across separate events is counted multiple times.

Future form of the indicator

As newer and higher spatial resolution and more precise datasets become available, methods will be updated to produce more robust estimates of population exposure to sea-level rise and weather-related population displacement.

2.5 Aboriginal and Torres Strait Islander health and climate change

Data

Time spent on Country item from *Mayi Kuwayu: The National Study of Aboriginal and Torres Strait Islander Wellbeing*, Wave 2 Release 7.1 data file, as accessed under Mayi Kuwayu Data Governance Committee approval #D250629.

Methods

Weighted prevalence estimates of the amount of time spent on Country for Aboriginal and Torres Strait Islander adults by remoteness area (ASGS-RA).

The sample includes 5,067 Aboriginal and Torres Strait Islander adults aged 16 years and over who responded to the Wave 2 questionnaire of *Mayi Kuwayu: The National Study of Aboriginal and Torres Strait Islander Wellbeing*, between January 2021 and December 2025. Respondents were asked 'How much time do you spend on Country?', with response options: 'Want to but can't'; 'Not at all'; 'A little bit'; 'A fair bit'; 'A lot'. The sample was weighted using iterative proportional fitting with benchmark variables of age, sex, and remoteness, using population distributions from the Australian Bureau of Statistics' Census-based

Estimates of Aboriginal and Torres Strait Islander Australians, June 2021 [14]. Weighted prevalence and 95% Confidence Intervals were estimated for each response option for the Time spent on Country item. Analysis was conducted in Stata 18, data visualisation was conducted in R using R Studio (2024.04.2+764).

Caveats

Time spent on Country is an indirect indicator of climate change and health, measuring Aboriginal and Torres Strait Islander Peoples' access to Country. The indicator does not distinguish whether 'Country' is the respondent's ancestral homelands or other Country to which the respondent may be connected. The indicator is only one aspect of the multi-faceted relationships that exist between climate change, Country, and Aboriginal and Torres Strait Islander peoples' wellbeing. Disaggregation by remoteness gives some insight into the variation of this indicator geospatially, however it does not show variation across different Aboriginal and Torres Strait Islander nations or cultures.

Future form of the indicator

Further research is being undertaken by Aboriginal and Torres Strait Islander research groups to refine an Aboriginal and Torres Strait Islander climate and health indicator that aligns with cultural values.

Section 3: Mitigation actions and health co-benefits

3.1 Energy system and health

Data

Data for this indicator are consistent with those used for "3.1.1 Energy system and health" in Romanello et al. [1].

Methods

Methods for this indicator are consistent with those used for "3.1.1 Energy system and health" in Romanello et al. [1].

Caveats

Caveats for this indicator are consistent with those used for "3.1.1 Energy system and health" in Romanello et al. [1].

Future form of the indicator

Future iterations of this indicator can be strengthened by offering a more detailed analysis of changes in Australia's energy mix, including sub-national disaggregation for Australia, which would identify the jurisdictions driving aggregate decarbonisation trends and those lagging. The indicator can be aligned with the trajectories implied by Australia's Net Zero Plan and the 2035 target of a 62-70% reduction in emissions on 2005 levels, thereby permitting an explicit assessment of whether observed trends in carbon intensity are consistent with the trajectory required for compliance. Such alignment would shift the indicator from a descriptive to a diagnostic instrument and provide a clearer basis on which to assess the adequacy of mitigation policy in protecting population health.

3.2 Household energy use

Data

Data for Australia were sourced from Clean Energy Council [15-18] and the First Nations Clean Energy Strategy [19].

Methods

This indicator offers a descriptive summary.

Caveats

Caveats for this indicator are the same as those for Indicator 3.2 Clean household energy in Beggs et al. [20].

Future form of the indicator

This indicator can be expanded in several ways in subsequent years. First, more granular data on the health co-benefits of Australia's renewable electricity transition could be incorporated, including air pollution impacts associated with the displacement of coal- and gas-fired generation. Second, with the release of the First Nations Clean Energy Strategy 2024-2030 and its commitment to filling knowledge gaps about First Nations peoples' energy use and access [19], future iterations of this indicator should aim to integrate disaggregated data on energy access, reliability, and disconnections in remote and Indigenous communities as it becomes available. Third, the rapid uptake of household batteries and virtual power plants offers an opportunity to track the equity of distributed energy resource adoption, including across renters, social housing tenants, and lower-income households who have so far been

underrepresented in rooftop solar uptake. As we noted in previous assessments, persistent gaps in data collection for Indigenous and remote communities continue to hinder a comprehensive understanding of energy use and its environmental and health consequences, and addressing these gaps remains a priority.

3.3 Sustainable and healthy transport

Data

- Street-level imagery sourced from Mapillary (mapillary.com)
- Cities covered – Adelaide, Brisbane, Canberra, Hobart, Melbourne, Perth, Sydney
- Data was divided into 3 zones based on distance from city centre – 0-5, 5-10, 10-25km

Methods

For each year – zone combination, images were drawn randomly until 1000 images meeting the requirements could be collected. The requirement (presence of pedestrians/cyclists) was confirmed using the same methodology used for counting. Images were analysed using object detection deep learning model YOLOv11 [21].

Several metrics were used to capture transport presence/density:
Detection rate – ratio of number of images containing at least one person : number of total images sampled
Cyclist/pedestrians per image – average number of cyclists or pedestrians taken across all images with at least one present.

The detection rate captures the probability that a randomly selected street shows evidence of walking or cycling, while the per image metrics capture the density.

To verify reproducibility, several samples were repeated in Sydney to ensure sampling biases were minimal across runs. Detection results across all reported metrics were within acceptable variance for the repeated runs.

Caveats

The workflow inherits the weaknesses of the imagery provider (Mapillary) – inconsistent coverage, poor representation of urban and rural areas. The detection performance of the model is known to vary with image quality, crowd density, and occlusion.

Future form of the indicator

The indicator will be extended to different cities (globally), zones and different modes (e-scooters etc.).

3.4 Emissions from agricultural production and consumption

Data

Data for this indicator are the same as those used for "Indicator 3.3.1 Emissions from agricultural production and consumption" in Romanello et al. [1].

Methods

Methods for this indicator are the same as those used for "Indicator 3.3.1 Emissions from agricultural production and consumption" in Romanello et al. [1].

Caveats

Caveats for this indicator are the same as those used for "Indicator 3.3.1 Emissions from agricultural production and consumption" in Romanello et al. [1]. In addition, the data for this report do not include new estimates of land-use change emissions attributed to crops.

Future form of the indicator

Work is currently on-going to integrate the emissions impacts of land-use changes attributable to agricultural production. It is expected they will be fully addressed in the coming year.

3.5 Diet and health co-benefits

Data

Data for this indicator are the same as those used for "Indicator 3.3.2: Diet and health co-benefits" in Romanello et al. [1].

Methods

Methods for this indicator are the same as those used for "Indicator 3.3.2: Diet and health co-benefits" in Romanello et al. [1].

Caveats

Caveats for this indicator are the same as those for "Indicator 3.3.2: Diet and health co-benefits" in Romanello et al. [1].

Future form of the indicator

The future form of this indicator is the same as that for "Indicator 3.3.2: Diet and health co-benefits" in Romanello et al. [1].

3.6 Health-care sector emissions

Data

Data for this indicator are the same as those used for "Indicator 3.5 Healthcare Emissions" in Romanello et al. [1].

Methods

Methods for this indicator are the same as those used for "Indicator 3.5 Healthcare Emissions" in Romanello et al. [1].

Caveats

Caveats for this indicator are the same as those used for "Indicator 3.5 Healthcare Emissions" in Romanello et al. [1].

Future form of the indicator

This indicator could be expanded to include other environmental and social impacts of the health sector.

Section 4: Economics and finance

4.1 Economic losses due to climate-related extreme events

Data

Reported data is based on figures on total insured economic losses from disaster events provided in the Historical Catastrophe Data of the Insurance Council of Australia (ICA) [22].

The database includes recorded data from the ICA on disaster events that have occurred over the last 50 years in Australia.

Cumulative annual insured losses arising from bushfires, cyclones, flooding, hailstorms, storm flooding, tornados and other climate-related extreme events are considered. Extreme events related to earthquakes, arson, gas disruptions, etc. (that are also reported in the ICA database), have been excluded.

Methods

Methods for this indicator are the same as those used for "4.1. Economic losses due to climate-related extreme events" in Beggs et al. [5].

Caveats

Caveats for this indicator are the same as those used for "4.1. Economic losses due to climate-related extreme events" in Beggs et al. [5].

Future form of the indicator

An ideal form of this indicator would allow attribution of the total economic losses (instead of total insured economic losses) to events induced by climate change. However, such attribution is unlikely to be feasible. As such, it is not envisaged that this indicator will significantly alter.

4.2 Costs of heat-related mortality

Data

Data for this indicator are the same as those used for "Indicator 4.1.2 Costs of heat-related mortality" in Romanello et al. [1].

Methods

Methods for this indicator are the same as those used for "Indicator 4.1.2 Costs of heat-related mortality" in Romanello et al. [1].

Caveats

Caveats for this indicator are the same as those used for "Indicator 4.1.2 Costs of heat-related mortality" in Romanello et al. [1].

Future form of the indicator

Future modifications for this indicator are the same as those for "Indicator 4.1.2 Costs of heat-related mortality" in Romanello et al. [1].

4.3 Country preparedness for the transition to net zero

Data

Data for this indicator are the same as those used for "Indicator 4.2.4: Country preparedness for the transition to net zero" in Romanello et al. [1].

Methods

Methods for this indicator are the same as those used for "Indicator 4.2.4: Country preparedness for the transition to net zero" in Romanello et al. [1].

Caveats

Caveats for this indicator are the same as those used for "Indicator 4.2.4: Country preparedness for the transition to net zero" in Romanello et al. [1].

Future form of the indicator

Further development of the indicator will be consistent with that for "Indicator 4.2.4: Country preparedness for the transition to net zero" in Romanello et al. [1].

4.4 Clean energy investment

Data

The data for this indicator are sourced from the annual State of the Energy Market reports by the Australian Energy Regulator [23] and the Australian Energy Market Operator's Generation Information Page [24]. Numbers are reported as generation of MW capacity and not as investment in AUD.

Methods

Methods for this indicator are the same as those used for "Indicator 4.2 Coal and clean energy investment" in Beggs et al. [5].

Caveats

Caveats for this indicator are the same as those used for “Indicator 4.2 Coal and clean energy investment” in Beggs et al. [5].

Future form of the indicator

It is not envisaged that the form of this indicator will change over time.

4.5 Net value of fossil fuel subsidies and carbon prices

Data and Methods

Data and methods for this indicator are consistent with those used for “Indicator 4.5: Net value of fossil fuel subsidies and carbon prices” in Romanello et al. [1].

This indicator reports the net value of fossil fuel subsidies and carbon prices in Australia. The net carbon price is calculated as the carbon price less fossil fuel subsidies expressed per tonne of greenhouse gas emissions.

Fossil fuel subsidy data for Australia were obtained from the Organisation for Economic Co-operation and Development (OECD) Inventory of Support Measures for Fossil Fuels, accessed via the OECD Data Explorer (https://data-explorer.oecd.org) in March 2026, corresponding to version 3.0 of the dataset (December 2025 release; annual data). The OECD reported that Australia’s total fossil fuel subsidies were $12.4 billion in 2024 [25]; values for 2024 are preliminary and may contain OECD-generated estimates.

National greenhouse gas emissions data were obtained from the Quarterly Updates of Australia’s National Greenhouse Gas Inventory, published by the Australian Government Department of Climate Change, Energy, the Environment and Water. The value used is the National Inventory Total - inclusive of land use, land-use change and forestry - for the year to December 2024, as reported in the December 2024 Quarterly Update [26]. The total emissions of CO_2 were reported to be 446Mt for 2024.

Carbon price revenues were reported to be zero in 2025 [27]. On these inputs, the subsidy per tonne of CO_2 equals $27.8: the carbon price per tonne is zero, and the net carbon price (the carbon price less the subsidy) equals −$27.8 per tonne of CO_2.

Australia still does not have an explicit carbon price, relying on the Safeguard Mechanism, which came into effect in July 2023. This scheme requires large carbon emitters, defined as facilities that emit more than 100,000 tonnes of CO_2 per year, to offset emissions above an assigned baseline by purchasing and surrendering Safeguard Mechanism Credits or Australian Carbon Credit Units (ACCUs). Under the scheme, facilities can purchase carbon credits at a fixed cap price of $82.7 in 2025–26; however, because open-market spot prices are roughly half the fixed cap price, most facilities buy ACCUs on the secondary market [28]. Australia's carbon market has grown considerably through this channel: the total traded market value of ACCUs reached A$1.4 billion in 2025, more than 3.5 times the value of three years earlier. However, this value flows between private buyers and sellers of carbon credits — not to government as carbon price revenue.

Caveats

Caveats for this indicator are the same as those used for "Indicator 4.5: Net value of fossil fuel subsidies and carbon prices" in Romanello et al. [1].

Additionally, the OECD periodically revises historical fossil fuel subsidy estimates as new data become available, and the Australian Government revises national emissions figures in successive quarterly updates and annual inventories. These revisions mean that subsidy-per-tonne and net carbon price calculations for earlier years may differ from those reported in previous annual *MJA-Lancet* Countdown reports (Figure S18).

Section 5: Public and political engagement

5.1 Media coverage of health and climate change

Data

The data were collected by accessing the Factiva (Dow Jones) global news databases via the University of Sydney library. Thirteen Australian national or regional newspapers were included as sources, including The Australian, Australian Financial Review, Sydney Morning Herald, Sun Herald (Sydney), The Daily Telegraph (Sydney), The AGE (Melbourne), Herald Sun (Melbourne), Canberra Times (Canberra), The Courier Mail (Brisbane), The West Australian (Perth), Adelaide Advertiser (Adelaide), Hobart Mercury (Hobart), NT News (Darwin).

Methods

The search methods for major Australian newspapers were consistent with those used in our previous reports, with the addition of data covering the period from 1 January 2024 to 31 December 2025.

Temporal trends in the coverage of climate change and health topics were identified in thirteen selected newspaper sources across Australia and major cities from 1st January 2008 through 31st December 2025. These newspapers were selected based on four factors considered by the *Lancet* Countdown report on climate change and health, but modified with Australian contexts, including: (1) geographical diversity (covering all Australian state capital cities), (2) circulation (higher circulated newspapers), (3) both national and regional coverage, and (4) reliable access to archives over time in Factiva.

The following keywords were used for searching in 'headline and lead paragraph' and English publications only. The keywords were more comprehensive than those used in the global report, and can better reflect Australian contexts, including national climate risks and related health concerns. We also used search operators to include more relevant articles: AND - search for both terms; OR- search for either term; * Truncation-locate variant endings of a word; near N-search terms on either side of this operator must appear within up to the specified number of words of each other.

(malaria or dengue or diarrhoea or infecti or disease or sars or measles or pneumonia or epidemic or pandemic or public health or (health near5 care) or epidemiology or health or mortality or morbidity or nutrition or illness or disease or NCD or non-communicable disease or communicable disease or air pollut* or nutrition or malnutrition or mental or disorder or stunting) AND ((climate near5 change) or global warming or temperature* or extreme weather or global environmental change or climate variability or greenhouse or low carbon or greenhouse gas emission* or renewable energy or carbon emission* or co2 emission* or climate pollut*)*

In 2019, we introduced separate Factiva analyses of news and transcripts from the Australian Broadcasting Corporation (ABC) to capture trends in media coverage beyond newspapers. The ABC is Australia's main public broadcaster, delivering widely circulated news and current affairs across television, radio, and online platforms. Data from 2008 were first

included in the 2019 report, with additional data for 2024 and 2025 incorporated in the current report (Figure S19). The same search strategy and database were used throughout.

The consistent approach ensured comparability of results across years.

Caveats

The analyses did not include all media sources in Australia. Only ABC programs were included in addition to mainstream newspapers, but social media coverage was not analysed. The results are not directly comparable with the global report due to differences in methods, including key searching terms, additional Australian news agencies and the study periods.

Future form of the indicator

We can develop valid methods to analyse social media coverage in future reports.

5.2 Scientific articles on health and climate change

Data

Data for this indicator are the same as those used for “Indicator 5.3.1: Scientific articles on health and climate change” in Romanello et al. [1]. We used Australian and New Zealand country data (by affiliation) from the global dataset for this indicator.

Methods

Methods for this indicator are the same as those used for “Indicator 5.3.1: Scientific articles on health and climate change” in Romanello et al. [1]. We calculated trends between 2006 and 2025 using the data provided by the global team.

Caveats

Due to the changes to the methods, numbers in this report are not directly comparable to previous revisions of this indicator in the *MJA-Lancet* Countdown reports. The other caveats for this indicator are the same as those for “Indicator 5.3.1: Scientific articles on health and climate change” in Romanello et al. [1].

Future form of the indicator

Further development of the indicator will be consistent with those for “Indicator 5.3.1: Scientific articles on health and climate change” in Romanello et al. [1].

5.3 Scientific engagement on the health impacts of climate change

Data

Data for this indicator are the same as those used for "Indicator 5.3.2: Scientific engagement on the health impacts and climate change" in Romanello et al. [1]. We used Australian and New Zealand country data (by affiliation) from the global dataset for this indicator.

Methods

Methods for this indicator are the same as those used for "Indicator 5.3.2: Scientific engagement on the health impacts and climate change" in Romanello et al. [1]. We calculated trends between 2006 and 2025 using the data provided by the global team.

Caveats

The caveats for this indicator are the same as those for "Indicator 5.3.2: Scientific engagement on the health impacts and climate change" in Romanello et al. [1].

Future form of the indicator

Further development of the indicator will be consistent with those for "Indicator 5.3.2: Scientific engagement on the health impacts and climate change" in Romanello et al. [1].

5.4 Government engagement in health and climate change

Data

This indicator sought to identify the extent to which Australian governments were working on climate change as a health issue, and/or health was being addressed as an issue affected by climate change. The data used for this report were derived from the Parliamentary websites of the Commonwealth of Australia and it's eight States and Territories.

Jurisdiction	Parliamentary website
Commonwealth of Australia	https://www.aph.gov.au
Queensland	https://www.parliament.qld.gov.au/
New South Wales	https://www.parliament.nsw.gov.au/
Australian Capital Territory	https://www.parliament.act.gov.au/
Victoria	https://www.parliament.vic.gov.au/
Tasmania	https://www.parliament.tas.gov.au/
South Australia	https://www.parliament.sa.gov.au/
Western Australia	https://www.parliament.wa.gov.au/
Northern Territory	https://nt.gov.au/

The data collected were as follows: for the Commonwealth of Australia we looked for Bills and Legislation, Committees and Inquiries, and Chamber documents that included a consideration of climate change and health. For the States and Territories, we looked for relevant Bills and Legislation, Committees and Inquiries and overall Hansard records. Hansard is the report of proceedings and verbatim record of what was said in Parliament.

Methods

National

The datasets of the Parliament of Australia public website were searched (https://www.aph.gov.au/Parliamentary_Business) for records from 1 January 2024 to 31 December 2025.

Bills & legislation were searched by whole document and included all current and previous Bills sponsored by Government and Private Members/Senators. Committees & inquiries were searched using titles and summary and included all Senate, House and Joint committees, inquiries and hearings. Chamber documents were searched by whole document and included notice papers, tabled papers, votes and proceedings from the House and journals of the Senate.

Datasets were searched using each key search term for (a) climate change and for (b) health, as listed below. Searches were then conducted for (c) climate change AND health. Terms have been updated to reflect the changing terminology used to discuss climate change and the key health issues facing Australians.

(a) climate change:
"climate change", "changing climate", "climate energy", "climate action", "climate decay", "global warming", "temperature", "extreme weather", "global environmental change", "climate variability", "greenhouse", "low carbon", "ghge", "renewable energy", "clean energy", "carbon emission", "co2 emission", "carbon dioxide", "carbon-dioxide", "decarbonisation", "decarbonization", "carbon neutral", "carbon-neutral", "carbon neutrality", "carbon-neutrality", "net-zero", "net zero", "climate pollutant", "air pollution", "drought", "flood", "bushfire", "heat", "cyclone".

(b) health:

“malaria”, “dengue”, “diarrhoea”, “infection”, “disease”, “pneumonia”, “epidemic”, “pandemic”, “public health”, “health care”, “epidemiology”, “healthcare”, “health”, “mortality”, “morbidity”, “nutrition”, “illness”, “infectious”, “ncd”, “non-communicable disease”, “noncommunicable disease”, “chronic disease”, “communicable disease”, “malnutrition”, “mental disorder”, “mental health”, “depression”, “suicide”, “obesity” “death”, “injury”, “food”, “active travel”, “urban design”, “bicycle”, “alcohol”.

(c) climate change AND health:
“climate health”, “‘climate change’ AND ‘health’”

Any Bills & legislation identified through searches (a) and (b) were further analysed for overlap between climate change and health by key word searching within the legislation and explanatory memorandum. Documents identified through (a) climate change were searched using the term “health” and documents identified through (b) health were searched using the term “climate change”.

States and Territories

Parliamentary websites were searched for records from 1 January 2024 to 31 December 2025 using the datasets *Bills & Legislation and Committees & Inquiries*, as well as a total Hansard search. State and territory websites were only searched using terms (c) only: i.e., “climate health”, “climate change” AND “health”. Documents identified were hand searched for relevance.

Caveats

Documents that did not use health in the context of human health (e.g., ecosystem, ecological or reef health) were excluded.

Due to time constraints, we only used terms in category (c) for the states and territories.

State and territory websites search tools are quite variable and many do not have advanced search functionality, thereby requiring hand searching of all Bills introduced and Committee Inquiries held in 2024 and 2025.

At the national level, search (c) did not yield many results (Table S5). As such, (a) and (b) results for Bills were hand searched to identify documents that referenced climate change and health.

Due to the large number of search results, we did not hand search chamber documents from the Parliament of Australia to remove duplicates.

Future form of the indicator

The indicator could be improved over time by using (a) and (b) search terms in state and territory parliaments. However, search functionality of state and territory parliament websites are problematic, requiring hand searching of all Bills introduced and Committee Inquiries held that year.

5.5 Health and climate change research funding

Data

The Australian Government National Health and Medical Research Council's (NHMRC's) Research Grants Management System (RGMS) was analysed by NHMRC personnel for the annual number of research funding applications over the years 2000 to 2025 with a focus on health and climate change. All grant applications submitted to NHMRC are health-related. As such, grant applications were searched for one or more of the following climate keywords:

CHANGE_IN_CLIMATE
CHANGE_OF_CLIMATE
CHANGES_IN_CLIMATE
CLIMATE_CHANGE
CLIMATE_CHANGES
CLIMATE_PATTERN
CLIMATE_PATTERNS
CLIMATE_SYSTEM
CLIMATE_VARIABILITIES
CLIMATE_VARIABILITY
GLOBAL_TEMPERATE
GLOBAL_TEMPERATURE
GLOBAL_TEMPERATURES
GLOBAL_WARMING

GREENHOUSE
PATTERN_OF_WEATHER
PATTERNS_OF_WEATHER
WARMING_CLIMATE
WEATHER_PATTERN
WEATHER_PATTERNS

Grants are recorded against the year of application and are categorised into those that are funded and those that are not funded. The annual success rate was calculated by dividing the annual number of grants funded by the total number of grant applications each year and expressed as a percentage.

In order to benchmark the annual health and climate change grant application success rate, the overall annual NHMRC success rate was obtained from the NHMRC "Outcomes of funding rounds" webpage:
https://www.nhmrc.gov.au/funding/data-research/outcomes#download

Methods
The indicator tracks the annual number of health and climate change grant applications that are funded and not funded, and the corresponding success rate. The success rate for health and climate change grant applications is benchmarked against the overall annual NHMRC success rate.

Caveats
The number of applications is likely to be an underestimate due to the choice of category descriptors for areas of research in the RGMS [36].

Future form of the indicator
There is potential to develop this indicator by also reporting the annual dollar amounts funded and not funded, funding by funding program, funding by broad research area, funding by field of research, and other more detailed analysis of the data. In the future, this indicator could also track health and climate change research grant applications to the other major Australian Government funder of research, the Australian Research Council (ARC), and New Zealand funders of research.

5.6 Health and climate change litigation in Australia

Data

Data used for this indicator were records of Australian climate change litigation raising health issues located using the University of Melbourne's Australian and Pacific Climate Change Litigation Database, https://law.app.unimelb.edu.au/climate-change/index.php. This database records litigation (including settled cases and court orders) on issues of climate change in Australia, New Zealand and the Pacific Islands from 2000 to the present. It is maintained and regularly updated with new litigation records by Melbourne Climate Futures at the University of Melbourne. The database provides summaries of the litigation included, with links to the full text of court-issued judgments or orders in those proceedings.

Methods

A key word search was performed using the database general search function using the terms 'health', 'injury', 'illness', 'death', 'air pollution' 'life' and 'heat'. Terms needed to be searched one by one as the database cannot search for multiple terms simultaneously. Cases were limited to those filed in Australia between 2024-2025 or where a judgment was delivered for the Australian case between 2024-2025. Cases were excluded where they did not directly refer to health-related matters. Searches returned, for this period:

- 4 instances of cases mentioning the term 'health'
- 1 instance of cases mentioning the term 'injury'
- 0 instances of a case mentioning the term 'illness'
- 0 instances of cases mentioning the term 'death'
- 1 instance of cases mentioning the term 'heat'
- 0 instances of cases mentioning the term 'air quality'
- 0 instances of cases mentioning the term 'air pollution'
- 3 instances of cases mentioning the term 'life'

In addition, cases included in the database's 'human rights' and 'duty of care' categories were searched. This yielded 1 additional case. Finally, all cases filed in 2023, 2024 and 2025 were checked to see if there were any references to health-related matters. This yielded 9 additional cases.

Cases were then further screened. Cases that were filed but where no court decisions had been made as at 31 December 2025 were excluded. Cases were analysed to identify if any references to health-related matters were incidental or whether there was meaningful engagement with these issues. If there only an incidental reference to health-related matters, they were removed from the dataset.

This yielded a dataset of **13** cases between 2024-2025.

The full text judgments of court decisions or orders in each case record were read and analysed to determine the nature and significance of the health arguments made.

Caveats

The database provides a summary of cases but not the full text of relevant judgments. By searching the database with key terms, it may be that some relevant case records are missed. In this instance, the authors used their expert subject matter knowledge to verify that all relevant cases had been included.

While useful for indicating future trends, pending cases — where no court decision had yet been issued — were removed from the dataset.

Future form of the indicator

A capacity to include AI natural language processing to screen full text judgments for references to health could improve the comprehensiveness of the data set analysed over time.

We would expect over time that additional categories of climate cases raising health issues will emerge as the energy transition proceeds. This could include cases where communities raise health impacts in opposition to renewable energy/zero emissions energy projects despite the climate benefits of such proposals. The case law database records examples of cases of this kind in Australia pre-2014 associated with the early development of wind farms.

References

1. M. Romanello, M. Walawender, S.-C. Hsu, et al., "The 2026 report of the *Lancet* Countdown on health and climate change: ," *The Lancet* (2026).
2. C. Gao, J. Menssink, M. Ziou et al., *More than distress: How climate change is shaping young people's lives - Findings from the 2025 Mission Australia Youth Survey* (Orygen: Melbourne, Victoria and Mission Australia: Sydney, New South Wales, 2026), https://www.missionaustralia.com.au/what-we-do/evidence-impact-and-advocacy/research/reports/more-than-distress-youth-survey-report/ (viewed 01 June, 2026).
3. R. McHale, N. Brennan, T. Freeburn, E. Richardson, A. Rossetto, and M. Carmody, *Youth Survey Report 2025* (Sydney, New South Wales: Mission Australia, 2025), https://www.missionaustralia.com.au/publications/youth-survey (viewed 29 April, 2026).
4. T. J. Crandon, J. G. Scott, F. J. Charlson, and H. J. Thomas, "Coping with Climate Change: Correlates of Adaptive and Maladaptive Coping in Young People," *Chronic Stress* 10 (2026).
5. P. J. Beggs, A. J. Woodward, S. Trueck et al., "The 2024 report of the *MJA–Lancet* Countdown on health and climate change: Australia emerging as a hotspot for litigation," *Medical Journal of Australia* 222, no. 6 (2025): 272-296.
6. World Meteorological Organization, "Climate services dashboard," World Meteorological Organization, https://app.powerbi.com/view?r=eyJrIjoiZTMyNTVkNDMtZmZmMy00YTI2LWJkYjktYjA4MjJmMDg5ZTg4IiwidCI6ImVhYTZiZTU0LTQ2ODctNDBjNC05ODI3LWMwNDRiZDhlOGQzYyIsImMiOjl9 (viewed 16 February, 2026).
7. World Health Organization, "IHR capacity progress," World Health Organization, 2022, https://extranet.who.int/e-spar/Home/Capacity (viewed February 16, 2026).
8. World Health Organization, *International Health Regulations (2005): guidance document for the state party self-assessment annual reporting tool* (Geneva: World Health Organization, 2018), https://iris.who.int/bitstream/handle/10665/272438/WHO-WHE-CPI-2018.17-eng.pdf?sequence=1 (viewed 16 February, 2026).
9. World Health Organization, *International Health Regulations (2005), 3rd ed [as amended in 2014, 2022, and 2024]* (Geneva: World Health Organization, 2025), https://apps.who.int/gb/bd/pdf_files/IHR_2014-2022-2024-en.pdf (viewed 16 February, 2026).
10. M. Romanello, M. Walawender, S.-C. Hsu et al., "The 2025 report of the *Lancet* Countdown on health and climate change: climate change action offers a lifeline," *The Lancet* 406, no. 10521 (2025): 2804-2857.
11. Internal Displacement Monitoring Centre, "Disaster Displacement Data," 2026, https://www.internal-displacement.org/database/displacement-data/ (viewed 10 March, 2026).
12. World Bank Group, "Population, total - Australia," The World Bank Group, 2026, https://data.worldbank.org/indicator/SP.POP.TOTL?locations=AU (viewed 26 February, 2026).
13. World Bank Group, "Population, total - New Zealand," The World Bank Group, 2026, https://data.worldbank.org/indicator/SP.POP.TOTL?locations=NZ (viewed 26 February, 2026).
14. Australian Bureau of Statistics, "Estimates of Australian Aboriginal and Torres Strait Islander Australians," ABS, 2021, https://www.abs.gov.au/statistics/people/aboriginal-and-torres-strait-islander-peoples/estimates-aboriginal-and-torres-strait-islander-australians/latest-release (viewed 20 March, 2026).
15. Clean Energy Council, *Clean Energy Australia 2024* (Melbourne: Clean Energy Council, 2024), https://cleanenergycouncil.org.au/news-resources/clean-energy-australia-report-2024 (viewed 05 March, 2026).
16. Clean Energy Council, *Rooftop solar and storage report: July to December 2024* (Melbourne: Clean Energy Council, 2025), https://cleanenergycouncil.org.au/news-resources/rooftop-solar-and-storage-report-july-to-december-2024 (viewed 05 March, 2026).

17. Clean Energy Council, *Clean Energy Australia 2025* (Melbourne: Clean Energy Council, 2025), https://cleanenergycouncil.org.au/news-resources/clean-energy-australia-report-2025 (viewed 05 March, 2026).
18. Clean Energy Council, *Rooftop solar and storage report: January to June 2025* (Melbourne: Clean Energy Council, 2025), https://cleanenergycouncil.org.au/news-resources/rooftop-solar-and-storage-report-january-to-june-2025 (viewed 05 March, 2026).
19. Australian Government, Department of Climate Change, Energy, the Environment and Water, *The First Nations Clean Energy Strategy 2024-2030* (Canberra: Commonwealth of Australia, 2024), https://www.energy.gov.au/energy-and-climate-change-ministerial-council/working-groups/community-engagement-working-group/first-nations-engagement-sub-working-group/first-nations-clean-energy-strategy (viewed March 05, 2026).
20. P. J. Beggs, S. Trueck, M. K. Linnenluecke et al., "The 2023 report of the *MJA–Lancet* Countdown on health and climate change: sustainability needed in Australia's health care sector," *Medical Journal of Australia* 220, no. 6 (2024): 282-303.
21. G. Jocher, A. Chaurasia, and J. Qiu, “Ultralytics YOLO,” ed. Editor (City: GitHub, 2024).
22. Insurance Council of Australia, "Data Hub, Historical Normalised Catastrophe list – February 2026," 2026, https://insurancecouncil.com.au/industry-members/data-hub/ (viewed 02 May, 2026).
23. Australian Government, Australian Energy Regulator, *State of the Energy Market 2025* (Canberra: Commonwealth of Australia, 2025), https://www.aer.gov.au/publications/reports/performance/state-energy-market-2025 (viewed March 05, 2026).
24. Australian Energy Market Operator, "Generation information," AEMO, 2026, https://www.aemo.com.au/energy-systems/electricity/national-electricity-market-nem/nem-forecasting-and-planning/forecasting-and-planning-data/generation-information (viewed 30 April, 2026).
25. Organisation for Economic Co-operation and Development, "OECD Data Explorer," 2025, https://data-explorer.oecd.org/vis?lc=en&tm=fossil%20fuel%20support&pg=20&snb=89&vw=ov&df[ds]=dsDisseminateFinalDMZ&df[id]=DSD_FFS%40DF_FFS_AUS&df[ag]=OECD.TAD.ADM&df[vs]=3.0&dq=A.........&pd=2010%2C&to[TIME_PERIOD]=false (viewed March 05, 2026).
26. Australian Government, Department of Climate Change, Energy, the Environment and Water, *Quarterly Update of Australia's National Greenhouse Gas Inventory: December 2024* (Canberra: Commonwealth of Australia, 2025), https://www.dcceew.gov.au/climate-change/publications/national-greenhouse-gas-inventory-quarterly-update-december-2024 (viewed March 05, 2026).
27. World Bank Group, "State and Trends of Carbon Pricing Dashboard," The World Bank Group, 2026, https://carbonpricingdashboard.worldbank.org/ (viewed 05 March, 2026).
28. Australian Government, Clean Energy Regulator, "Cost containment measure," Clean Energy Regulator, 2026, https://cer.gov.au/schemes/safeguard-mechanism/managing-excess-emissions/cost-containment-measure (viewed March 05, 2026).
29. Australian Government, Department of the Environment and Energy, *Quarterly Update of Australia's National Greenhouse Gas Inventory: December 2017* (Canberra: Commonwealth of Australia, 2018), https://www.dcceew.gov.au/climate-change/publications/national-greenhouse-gas-inventory-quarterly-update-december-2024 (viewed March 05, 2026).
30. Australian Government, Department of the Environment and Energy, *Quarterly Update of Australia's National Greenhouse Gas Inventory: December 2018* (Canberra: Commonwealth of Australia, 2019), https://www.dcceew.gov.au/climate-change/publications/national-greenhouse-gas-inventory-quarterly-update-december-2024 (viewed March 05, 2026).
31. Australian Government, Department of Industry Science Energy and Resources, *Quarterly Update of Australia's National Greenhouse Gas Inventory: December 2019* (Canberra: Commonwealth of Australia, 2020), https://www.dcceew.gov.au/climate-

change/publications/national-greenhouse-gas-inventory-quarterly-update-december-2024 (viewed March 05, 2026).
32. Australian Government, Department of Industry Science Energy and Resources, *Quarterly Update of Australia's National Greenhouse Gas Inventory: December 2020* (Canberra: Commonwealth of Australia, 2021), https://www.dcceew.gov.au/climate-change/publications/national-greenhouse-gas-inventory-quarterly-update-december-2024 (viewed March 05, 2026).
33. Australian Government, Department of Industry Science Energy and Resources, *Quarterly Update of Australia's National Greenhouse Gas Inventory: December 2021* (Canberra: Commonwealth of Australia, 2022), https://www.dcceew.gov.au/climate-change/publications/national-greenhouse-gas-inventory-quarterly-update-december-2024 (viewed March 05, 2026).
34. Australian Government, Department of Climate Change, Energy, the Environment and Water, *Quarterly Update of Australia's National Greenhouse Gas Inventory: December 2022* (Canberra: Commonwealth of Australia, 2023), https://www.dcceew.gov.au/climate-change/publications/national-greenhouse-gas-inventory-quarterly-update-december-2024 (viewed March 05, 2026).
35. Australian Government, Department of Climate Change, Energy, the Environment and Water, *Quarterly Update of Australia's National Greenhouse Gas Inventory: December 2023* (Canberra: Commonwealth of Australia, 2024), https://www.dcceew.gov.au/climate-change/publications/national-greenhouse-gas-inventory-quarterly-update-december-2024 (viewed March 05, 2026).

36. Australian Government, National Health and Medical Research Council, *Background paper: NHMRC's role in addressing health implications of environmental change* (Canberra: NHMRC, 2019), https://www.nhmrc.gov.au/sites/default/files/documents/attachments/communique-addressing-health-environmental-change.pdf (viewed May 05, 2026).

Table S1. Heatwave exposure (days per person per year) in Australia and New Zealand, by age group (≥65 years and <1 year), shown for 2025 and selected periods (1986-2005 baseline, 2006-2015, 2016-2025, and 2021-2025). A heatwave is defined as ≥3 consecutive days during which both daily minimum and maximum temperatures exceed the location-specific 95th percentile of the 1986 - 2005 baseline. Percentage changes are period means relative to the 1986–2005 baseline mean.

		Heatwave days per person per year					Change relative to baseline (1986-2005)			
**Age group**	**Country**	**2025**	**1986 to 2005**	**2006 to 2015**	**2016 to 2025**	**2021 to 2025**	**2025 vs baseline**	**2006 to 2015 vs baseline**	**2016 to 2025 vs baseline**	**2021 to 2025 vs baseline**
Adults aged ≥ 65	Australia	4.61	2.74	3.76	4.63	2.88	68.2%	37.2%	68.8%	5.1%
	New Zealand	2.48	3.37	2.38	7.14	4.41	-26.4%	-29.3%	112.0%	31.0%
Infants aged <1	Australia	4.73	2.76	3.64	4.58	2.98	71.8%	32.1%	66.3%	8.0%
	New Zealand	2.63	3.52	2.57	7.35	4.61	-25.2%	-26.9%	108.9%	31.0%

Table S2. Heatwave days attributable to climate change (days per person) in Australia and New Zealand by period. Observed values represent total heatwave exposure (counterfactual + attributable); counterfactual values represent expected heatwave exposure in the absence of anthropogenic climate change, and attributable values represent the additional heatwave days due to climate change. Estimates are population-weighted using a fixed 2021–2025 average to isolate the climate signal from demographic change and are not stratified by age group.

Country	Values	Time period				
		2025	2006 to 2015	2006 to 2025	2016 to 2025	2021 to 2025
Australia	Observed	4.65	3.39	3.62	3.85	3.07
	Counterfactual	1.67	1.82	1.55	1.28	1.08
	Attributable	2.98	1.57	2.07	2.57	1.99
	Attributable (%)	64.0	46.3	57.2	66.8	64.8
New Zealand	Observed	1.72	3.14	5.85	8.56	5.44
	Counterfactual	0.32	0.44	1.39	2.34	1.45
	Attributable	1.40	2.70	4.46	6.22	3.99
	Attributable (%)	81.3	85.9	76.3	72.7	73.3

Table S3. Average annual (*) bushfire exposure (person-days), bushfire risk (days) and bushfire-related $PM_{2.5}$ ($\mu g/m^3$) in Australia and New Zealand: 2006-2015, 2016-2025, and % change.

Country	Period	Bushfire exposure *(person-days)*	Bushfire risk *(days)*	Bushfire-related $PM_{2.5}$ *($\mu g/m^3$)*
Australia	2006-2015	362,256	27.7	0.41
	2016-2025	394,305	28.8	0.45
	% change	8.9%	3.9%	9.5%
New Zealand	2006-2015	14,970	0.05	0.082
	2016-2025	12,864	0.18	0.088
	% change	-14.1%	253.7%	7.7%

(*) All three sub-indicators were population-weighted to obtain country-level estimates.

Table S4. Detection rate (proportion of scanned images containing ≥1 detected human) and sample size (n) by city, distance zone, and year, with mean cyclists and pedestrians per image for 2024–2025. Cells showing '—' indicate strata with n < 10 human-containing images. Darwin is excluded (insufficient coverage).

City	Zone	2022 n / hit%	2023 n / hit%	2024 n / hit%	2025 n / hit%	Avg cyclists per image (2024–25)	Avg pedestrians per image (2024–25)
Sydney	0-5km	1,000 37.2%	1,000 21.7%	1,000 23.5%	1,000 30.8%	0.071	2.723
	5-10km	1,000 26.1%	1,000 10.3%	1,000 15.6%	1,000 16.1%	0.061	1.791
	10-25km	1,000 17.8%	1,000 6.3%	1,000 8.1%	1,000 6.7%	0.087	1.508
Melbourne	0-5km	1,000 54.6%	855 17.9%	1,000 44.8%	1,000 34.3%	0.226	2.430
	5-10km	1,000 46.0%	635 10.2%	1,000 20.4%	1,000 11.0%	0.073	1.488
	10-25km	1,000 5.6%	654 3.3%	1,000 37.5%	1,000 8.0%	0.014	1.185
Brisbane	0-5km	15 8.7%	17 8.7%	815 31.3%	1,000 10.2%	0.157	1.626
	5-10km	16 3.8%	6 2.3%	699 6.3%	1,000 5.1%	0.040	1.253
	10-25km	86 4.0%	97 11.6%	715 3.6%	444 2.2%	0.069	1.130
Perth	0-5km	—	107 6.9%	219 10.5%	119 8.7%	0.036	1.284
	5-10km	16 10.4%	82 4.7%	150 3.6%	689 5.6%	0.075	1.256
	10-25km	160 15.7%	33 2.1%	352 1.8%	474 2.4%	0.021	1.222
Adelaide	0-5km	15 4.0%	399 20.0%	134 18.2%	1,000 14.8%	0.090	1.422
	5-10km	344 6.1%	688 7.4%	238 30.8%	1,000 6.0%	0.021	1.242
	10-25km	205 7.7%	646 12.1%	197 13.0%	1,000 6.6%	0.019	1.577
Hobart	0-5km	202 25.1%	384 83.3%	1,000 53.6%	14 2.5%	0.003	3.890
	5-10km	74 6.7%	7 7.4%	1 5.3%	16 1.1%	0.000	1.000
	10-25km	519 2.6%	86 1.6%	111 17.2%	182 1.6%	0.000	1.775
Canberra	0-5km	63 4.6%	953 25.6%	435 3.5%	621 5.2%	0.148	1.298
	5-10km	94 1.6%	104 5.6%	191 2.4%	196 3.2%	0.026	1.207
	10-25km	168 2.7%	8 0.3%	194 1.3%	324 2.3%	0.058	1.156

Table S5. Total number of national Parliament of Australia documents related to climate change, health and climate change and health.

		(a) Climate change	(b) Health	(c) Climate change AND health
**Bills & legislation**	Passed	21	20	0
	Not passed	7	9	1
	Before parliament	4	4	0
	**Sub-total**	**32**	**33**	**1**
**Committees & inquiries**	Committees	2	3	0
	Inquiries	9	11	1
	Hearings	40	17	0
	**Sub-total**	**51**	**31**	**1**
**Chamber documents***		460	705	329

Note: * The Bill identified using (c) search terms was also identified using both (a) and (b) search terms – [Climate Change Amendment (Duty of Care and Intergenerational Climate Equity) Bill 2025]. It is an updated version of the 2023 Bill listed in the 2024 *MJA-Lancet* Countdown Report [5]. There were 16 separate issues discussed in the included inquiries and hearings. The number of chamber documents may contain duplicates across different search terms.

Table S6. Total number of bills, committees and inquiries, and Hansard results related to climate change and health in Australian State and Territory Parliaments.

Jurisdiction	Bills	Committees & Inquiries	Total Hansard results
QLD	2	1	61
NSW	4	9	325
ACT	5	12	157
VIC	5	8	147
SA	7	3	110
TAS	0	3	113
WA	1	6	71
NT	0	0	56
**All States and Territories**	**24**	**40**	**1040**

Table S7. Climate health related bills and inquiries before Australian State and Territory Parliaments.

Jurisdiction	Bill or Inquiry title	Sponsor/ Committee*
QLD	Disaster Management and Other Legislation Amendment Bill 2024	Opposition (ALP)
	Environmental Protection (Powers and Penalties) and Other Legislation Amendment Bill 2024	Opposition (ALP)
	2025-2026 Budget Estimates	Health, Environment and Innovation Committee
NSW	Environment Protection Legislation Amendment (Stronger Regulation and Penalties) Bill 2024	Government (ALP)
	Environmental Legislation Amendment Bill 2025	Government (ALP)
	Environmental Planning and Assessment Amendment (Planning System Reforms) Bill 2025	Government (ALP)
	Product Lifecycle Responsibility Bill 2025	Government (ALP)
	Inquiry into clean indoor air	Portfolio Committee No. 2 - Health
	2024 Annual Report of the Net Zero Commission	Joint Standing Committee on Net Zero Future
	Inquiry on PFAS Contamination in Waterways and Drinking Water Supplies Throughout New South Wales	Select Committee on PFAS Contamination in Waterways and Drinking Water Supplies Throughout New South Wales
	Impact of Renewable Energy Zones (REZ) on rural and regional communities and industries in New South Wales	Portfolio Committee No. 4 - Regional NSW
	Inquiry on electric and hybrid vehicle batteries[+]	Joint Standing Committee on Road Safety
	Inquiry Climate Change (Net Zero Future) Bill 2023[+]	Planning and Environment Committee
	2024 review of the annual reports and other matters of the Office of the Advocate for Children and Young People and the Office of the Children's Guardian[+]	Committee on children and young people
	Inquiry on the planning system and the impacts of climate change on the environment and communities[+]	Portfolio Committee No. 7 - Planning and Environment
	Inquiry on the Feasibility of Undergrounding the Transmission Infrastructure for Renewable Energy Projects[+]	Select Committee on the Feasibility of Undergrounding the Transmission Infrastructure for Renewable Energy Projects
ACT	Environment Protection Legislation Amendment Bill 2024	Government (ALP/ GRN)
	Environment Protection (Fossil Fuel Company Advertising) Amendment Bill 2024	Government (ALP/ GRN)

	Human Rights (Housing) Amendment Bill 2025	Private members (GRN)
	Planning (Territory Priority Project) Amendment Bill 2025	Government (ALP/ GRN)
	Inquiry into unpaid work	Standing Committee on Economy and Gender and Economic Equality
	Inquiry into Loneliness and Social Isolation in the ACT	Standing Committee on Education and Community Inclusion
	Inquiry into the ACT environment's bushfire preparedness⁺	Standing Committee on Environment, Climate Change and Biodiversity
	Inquiry into Petition 17-23: Indian (Common) Myna Control	Standing Committee on Environment, Climate Change and Biodiversity
	Inquiry into Climate change and a just transition	Standing Committee on Environment, Climate Change and Biodiversity
	Inquiry into Raising Children in the ACT	Standing Committee on Health and Community Wellbeing
	Inquiry into Appropriation Bill 2024-2025 and Appropriation (Office of the Legislative Assembly) Bill 2024-2025	Select Committee on Estimates 2024-2025
	Inquiry into Insurance Costs in the ACT	Standing Committee on Economics, Industry and Recreation
	Inquiry into Annual and Financial Reports 2024-25	Standing Committees on Legal Affairs, Environment and Planning, Public Accounts and Administration, and Social Policy
	Inquiry into the Role and Future of the Woden Town Centre in the context of a compact city	Standing Committee on Environment and Planning
	Inquiry into men's suicide rates	Standing Committee on Social Policy
	Inquiry into Appropriation Bill 2025-2026 and Appropriation (Office of the Legislative Assembly) Bill 2025-2026	Select Committee on Estimates 2025-2026
VIC	Entities Legislation Amendment (Consolidation and Other Matters) Bill 2025	Government (ALP)
	Sustainable Forests (Timber) Repeal Bill 2024	Government (ALP)
	Roads and Ports Legislation Amendment (Road Safety and Other Matters) Bill 2025	Government (ALP)
	Justice Legislation Amendment (Police and Other Matters) Bill 2025	Government (ALP)
	Planning Amendment (Better Decisions Made Faster) Bill 2025	Government (ALP)
	Inquiry into the supply of homes in regional Victoria	Legislative Assembly Environment and Planning Committee
	Inquiry into securing the Victorian food supply	Legislative Assembly Environment and Planning Committee
	Inquiry into Electricity Supply for Electric Vehicles	Legislative Council Economy and Infrastructure Com
	Inquiry into wildlife roadstrike in Victoria	Legislative Council Economy and Infrastructure Committee

	Inquiry into Climate Resilience	Legislative Council Environment and Planning Committee
	Inquiry into the 2022 Flood Event in Victoria[+]	Legislative Council Environment and Planning Committee
	Inquiry into food security in Victoria[+]	Legislative Council Legal and Social Issues Committee
	Inquiry into the 2024-25 financial and performance outcomes[+]	Public Accounts and Estimates Committee
SA	Climate Change and Greenhouse Emissions Reduction (Intergenerational Equity) Amendment Bill	Independent
	Drought Response and Recovery Coordinator Bill	Opposition (LP)
	Biodiversity Bill	Government (ALP)
	Emergency Management (Miscellaneous) Amendment Bill	Government (ALP)
	State Development Coordination and Facilitation Bill	Government (ALP)
	Plastic Shopping Bags (Waste Avoidance) Repeal Bill	Government (ALP)
	Climate Change and Greenhouse Emissions Reduction (Miscellaneous) Amendment Bill	Government (ALP)
	Inquiry into 2022-23 River Murray Flood Event	Select Committee on the 2022-23 River Murray Flood Event
	Inquiry into harmful Algal Blooms in South Australia	Joint Committee on Harmful Algal Blooms in South Australia
	Inquiry into recycling of Soft Plastics and Other Recyclable Material[+]	Select Committee on Recycling Soft Plastics & Other Recyclable Material
WA	Climate Action this Decade Bill 2025	Minor Party (GRN)
	Inquiry into community use of WA public school facilities	Education and Health Standing Committee
	Inquiry into the safety, regulation and penalties associated with the use of eRideables	Community Development and Justice Standing Committee
	Inquiry into support for health and medical research funding and priorities	Education and Health Standing Committee
	Annual Report 2023-24 (Committee report)	Education and Health Standing Committee
	Inquiry into how the Western Australian Government's progress towards achieving Environmental, Social and Governance (ESG) outcomes is assisting to secure international investment[+]	Public Accounts Committee

	New Bite - How alternative proteins could improve dietary and planetary health[+]	Education and Health Standing Committee
TAS	Annual report - Legislative Council Government Businesses Scrutiny Committees 2025	Legislative Council Government Businesses Scrutiny Committees 2025
	Annual report - Legislative Council Estimates Committees 2025	Legislative Council Estimates Committees 2025
	Annual report - House of Assembly Estimates Committees 2025	House of Assembly Estimates Committees 2025

Figure S1. Annual heatwave exposure time series for vulnerable populations in Australia and New Zealand, 1980–2025. (A) Heatwave exposure (days per person per year), adults aged ≥ 65 years. (B) Annual change in heatwave exposure (days per person) relative to the 1986-2005 baseline, adults aged ≥ 65 years. (C) Heatwave exposure (days per person per year), infants (<1 year). (D) Annual change in heatwave exposure (days per person) relative to the 1986-2005 baseline, infants (<1 year). In panels A and C, the light grey shading indicates the 1986-2005 baseline period used to derive the 95th-percentile temperature thresholds. In panels B and D, positive valued indicate higher exposure than the baseline average and negative values indicate lower exposure.

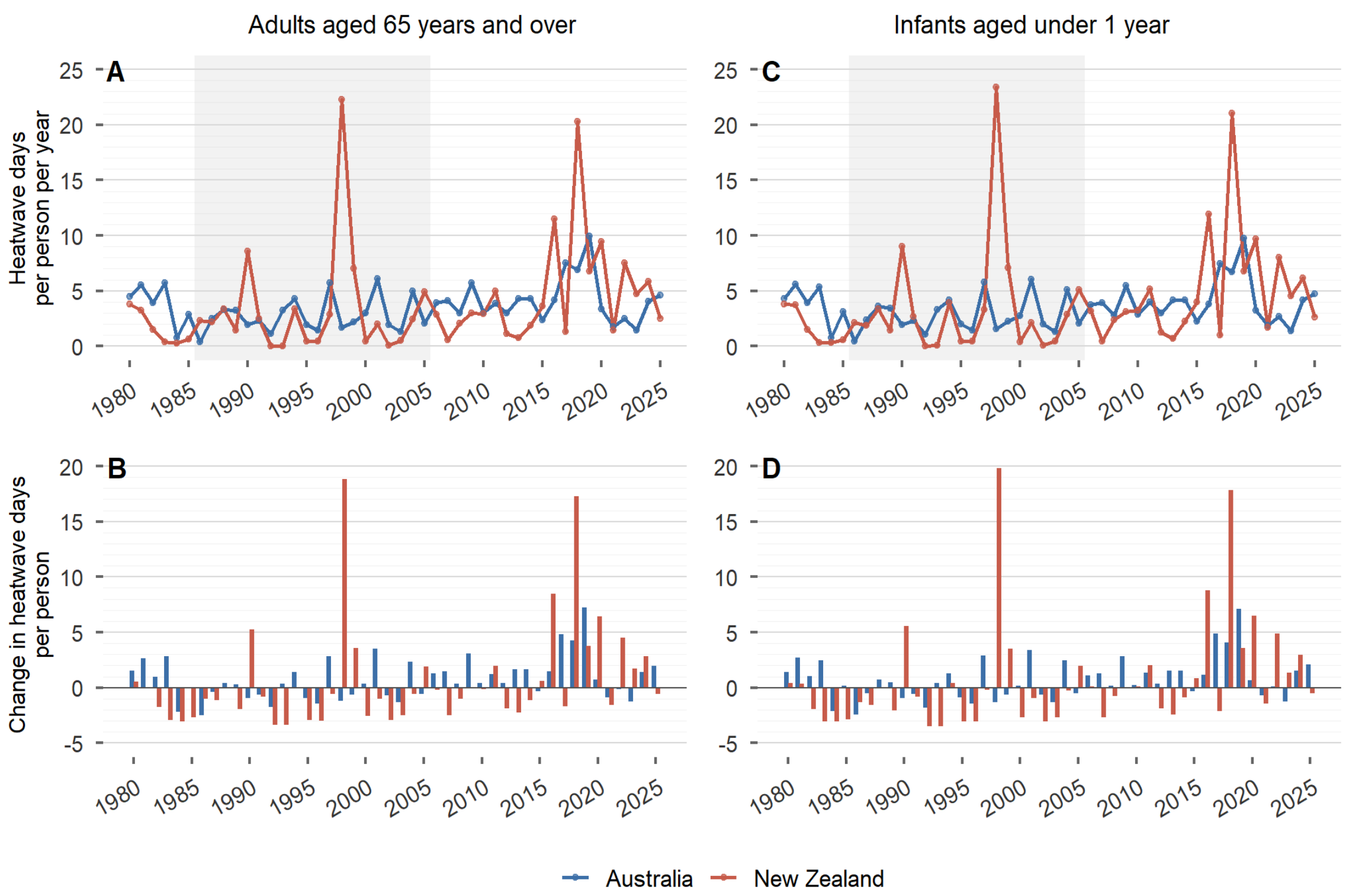

Figure S2. Implementation status (%) of International Health Regulations capacities C7 (Health Emergency Management) and C12 (Zoonotic Diseases) for Australia and New Zealand, 2021–2024, benchmarked against global and regional averages.

Data source: World Health Organization [7].

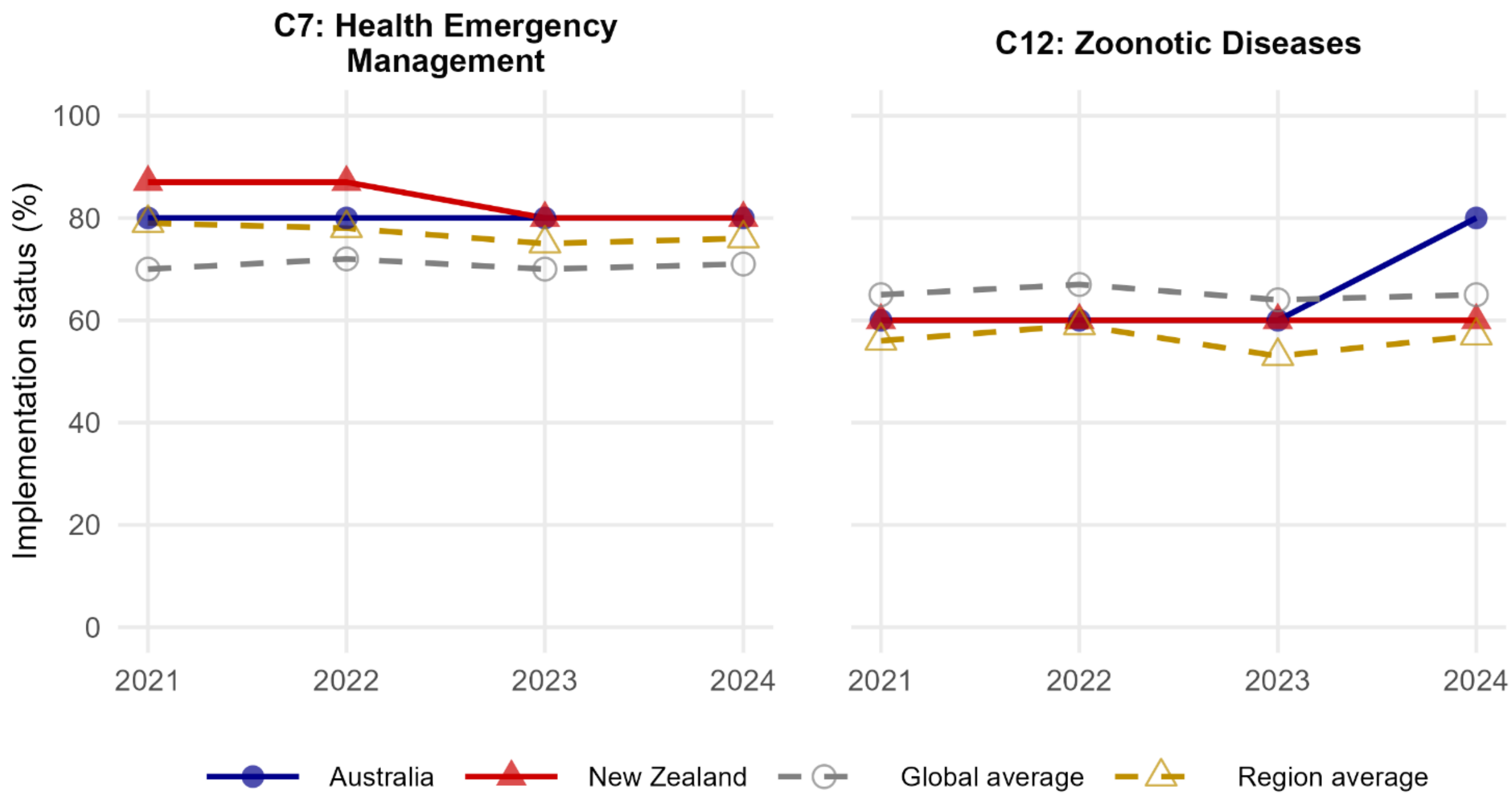

Figure S3. Mapillary image locations within 25 km of Sydney CBD, coloured by distance zone.

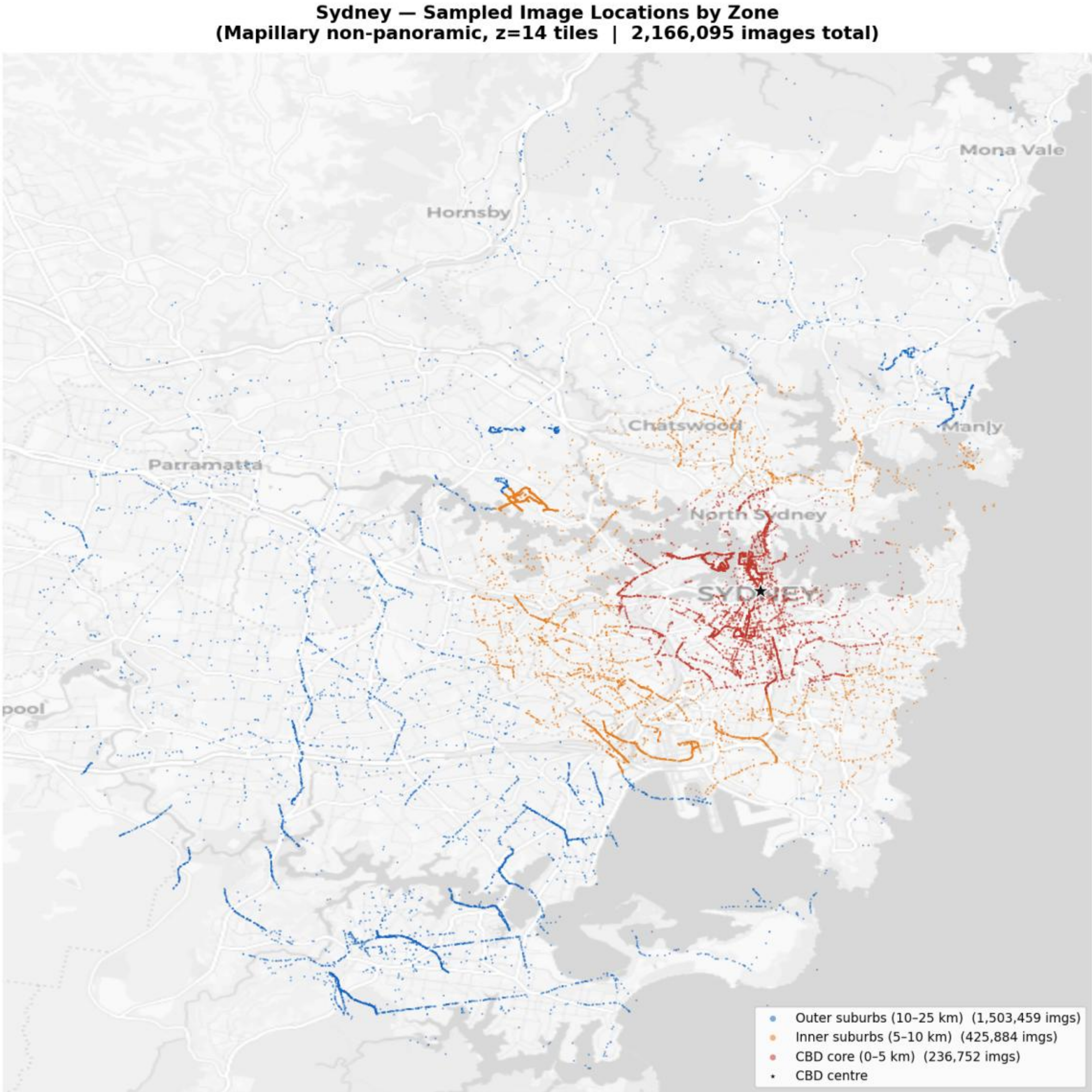

Figure S4. Mapillary image locations within 25 km of Melbourne CBD, coloured by distance zone.

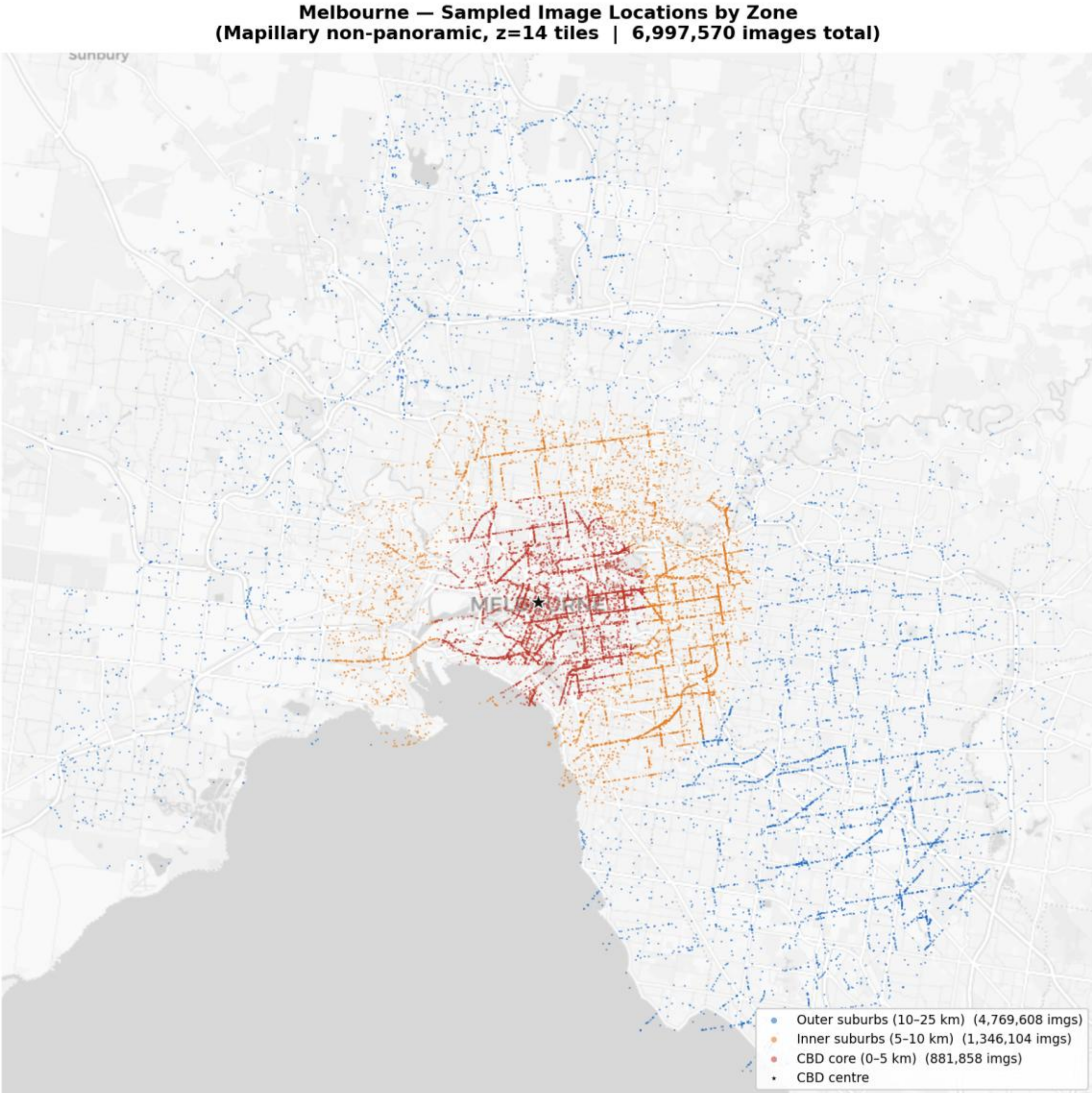

Figure S5. Mapillary image locations within 25 km of Brisbane CBD, coloured by distance zone.

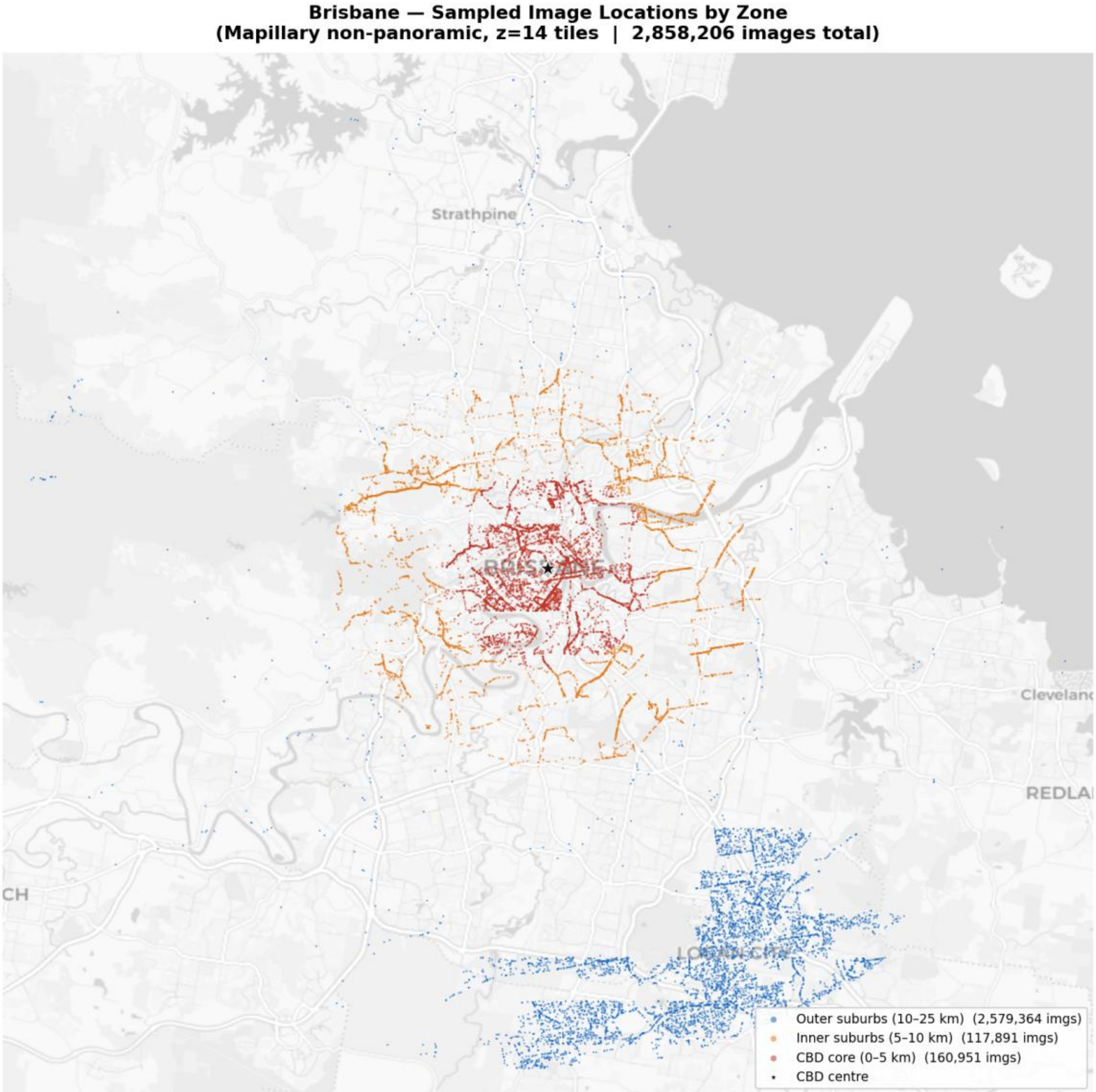

Figure S6. Mapillary image locations within 25 km of Perth CBD, coloured by distance zone.

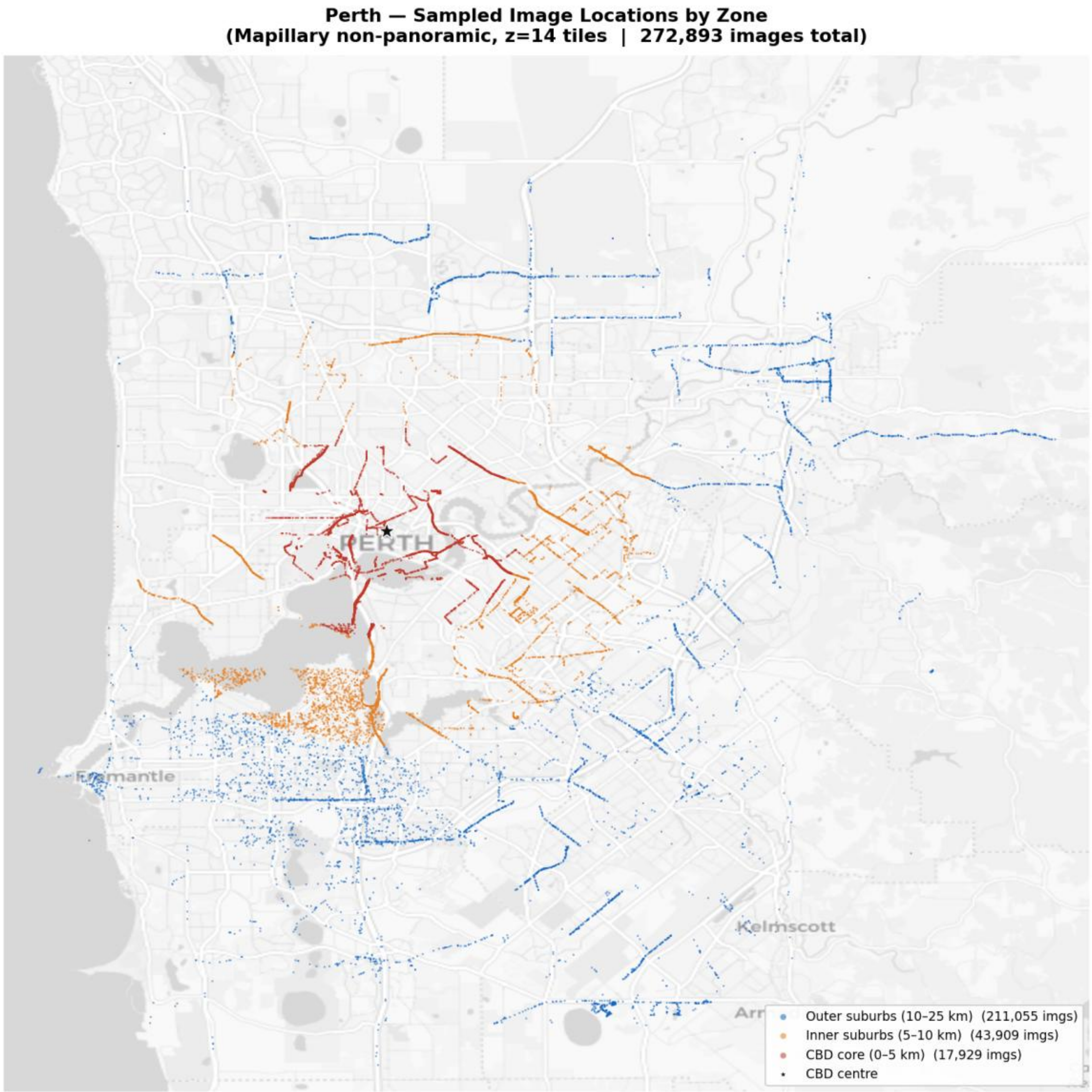

Figure S7. Mapillary image locations within 25 km of Adelaide CBD, coloured by distance zone.

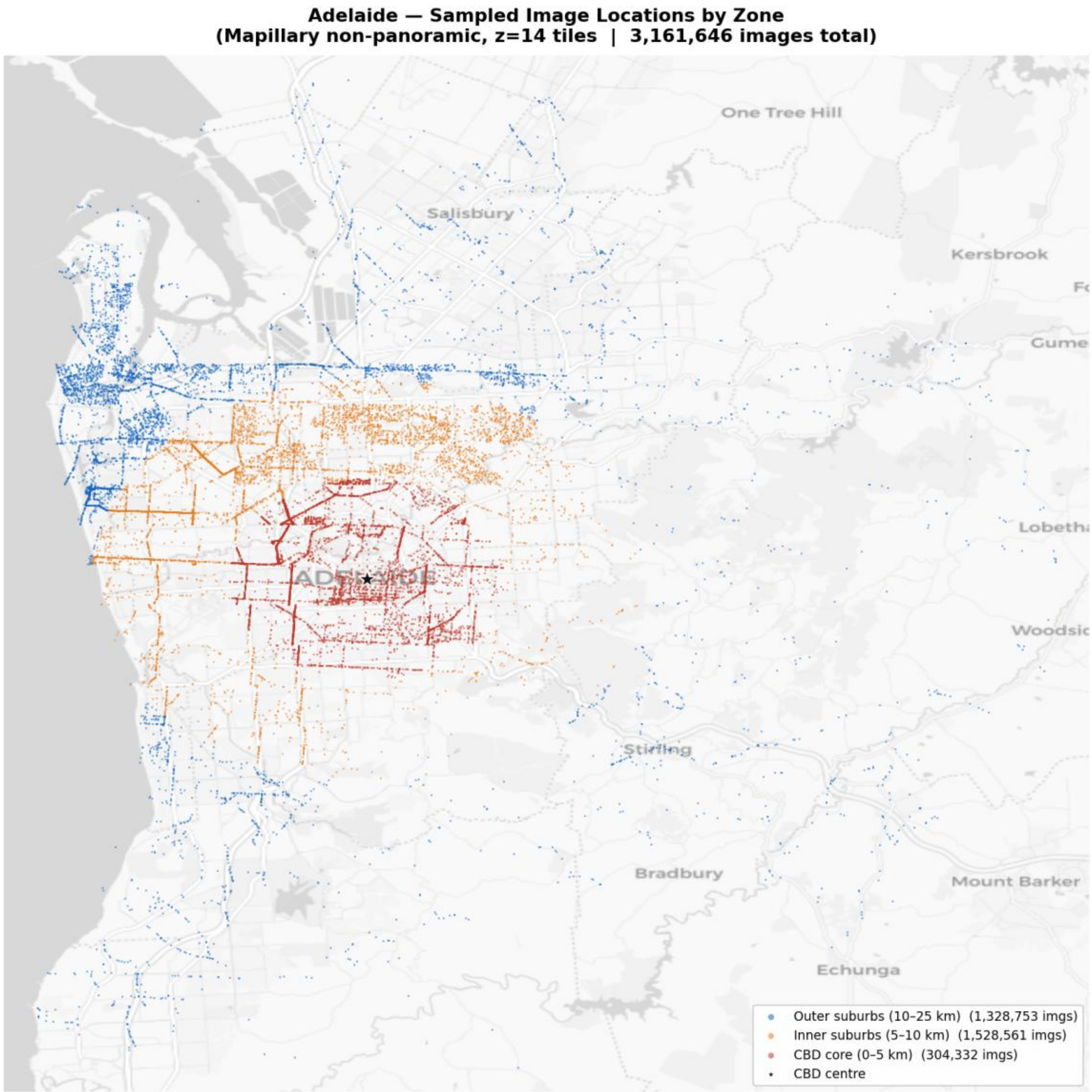

Figure S8. Mapillary image locations within 25 km of Hobart CBD, coloured by distance zone.

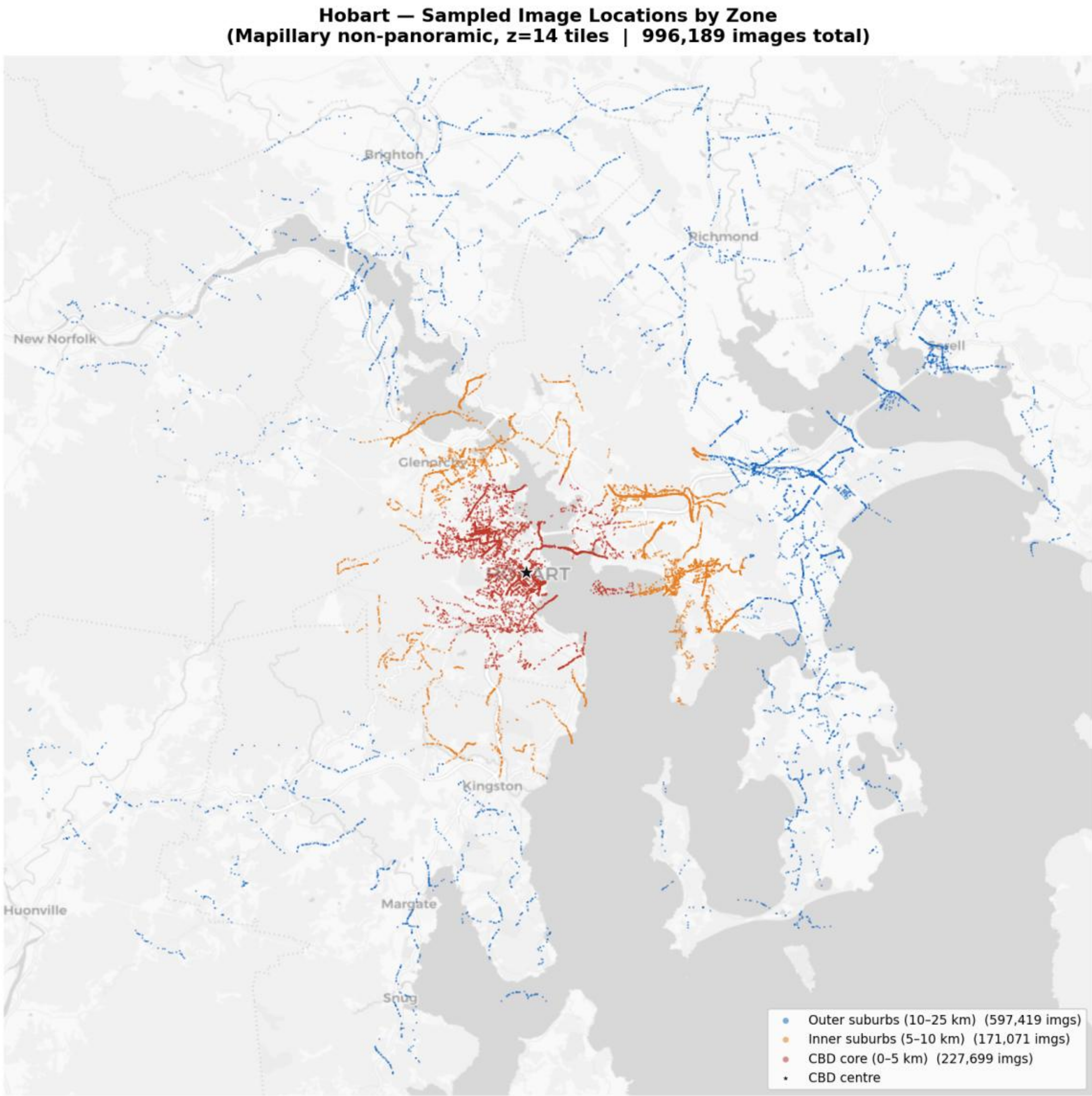

Figure S9. Mapillary image locations within 25 km of Canberra CBD, coloured by distance zone.

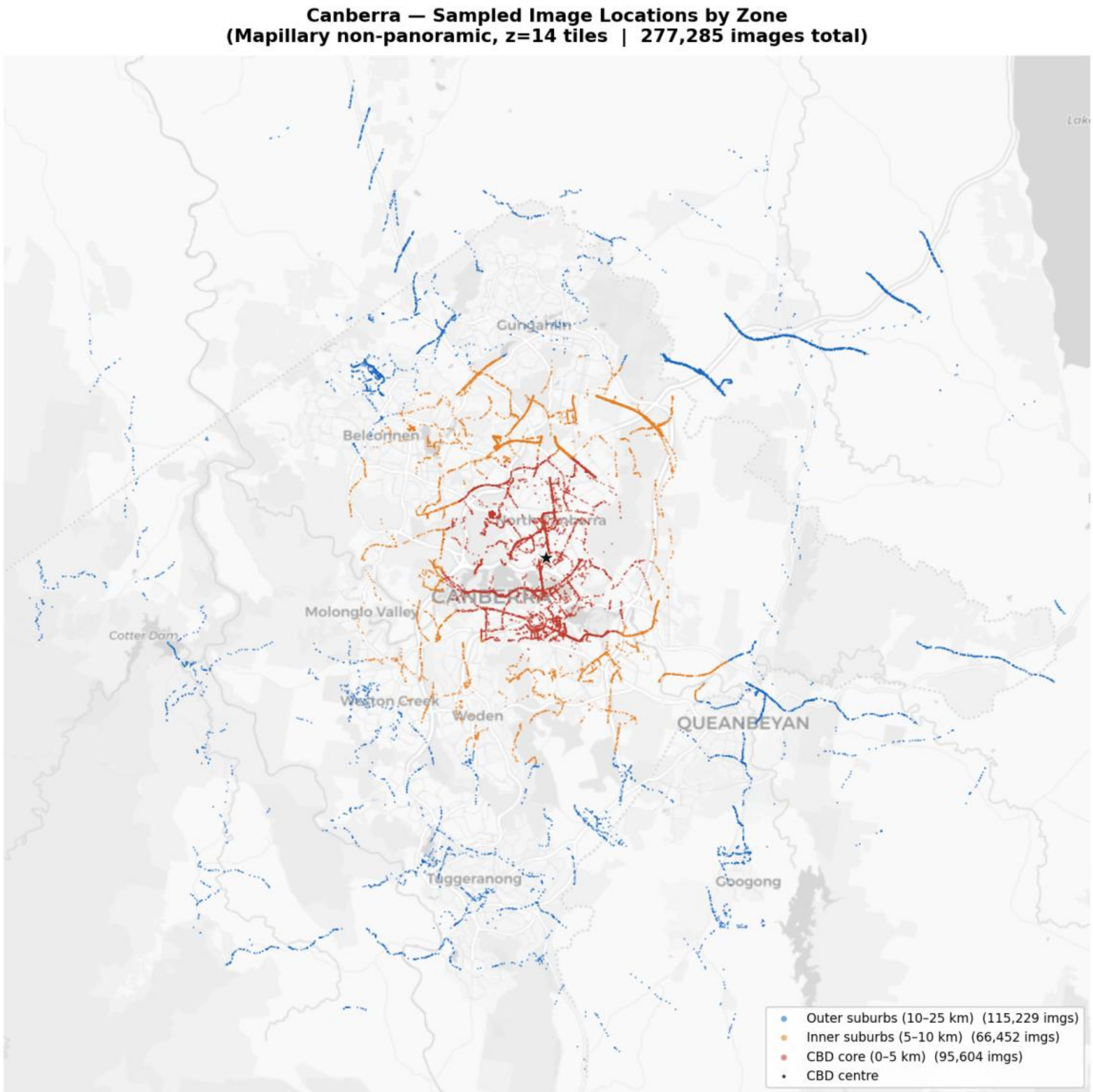

Figure S10. Brisbane, Perth and Adelaide - average cyclists and pedestrians per human-containing image by zone, 2022–2025.

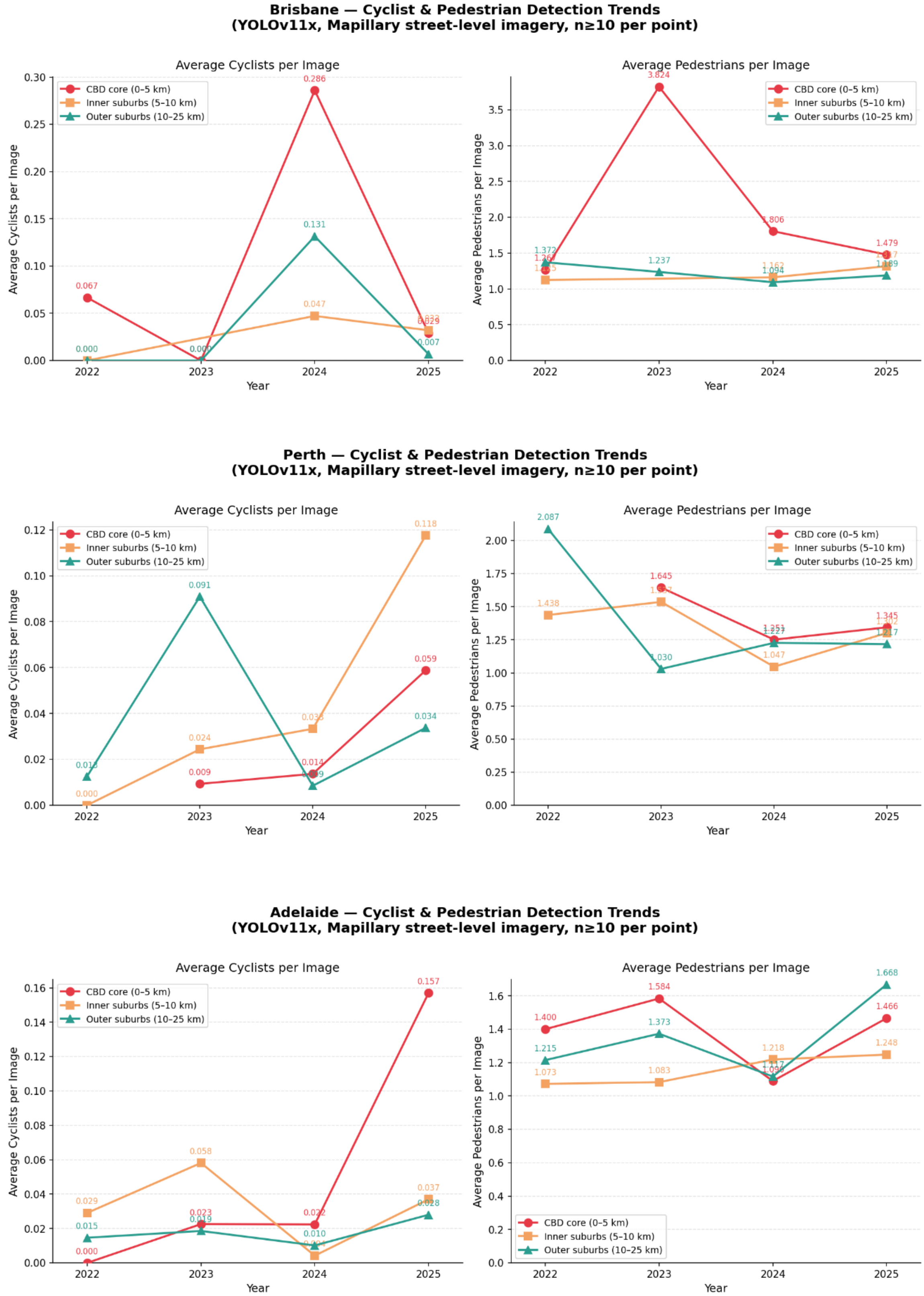

Figure S11. Hobart and Canberra - average cyclists and pedestrians per human-containing image by zone, 2022–2025.

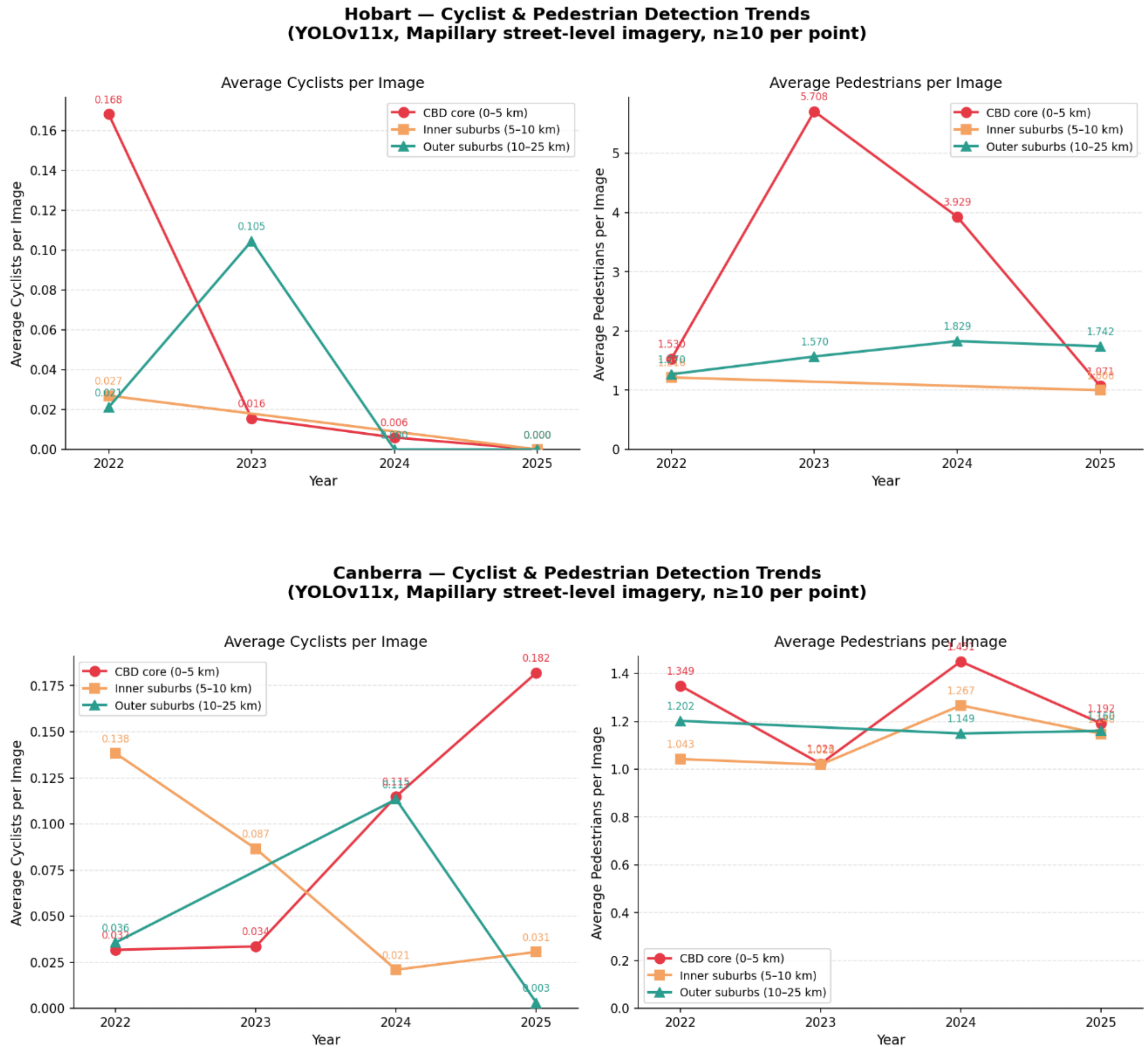

Figure S12. Sydney CBD (George Street), 2022 — multiple pedestrians detected. YOLOv11x detected 0 cyclist(s) and 13 pedestrian(s).

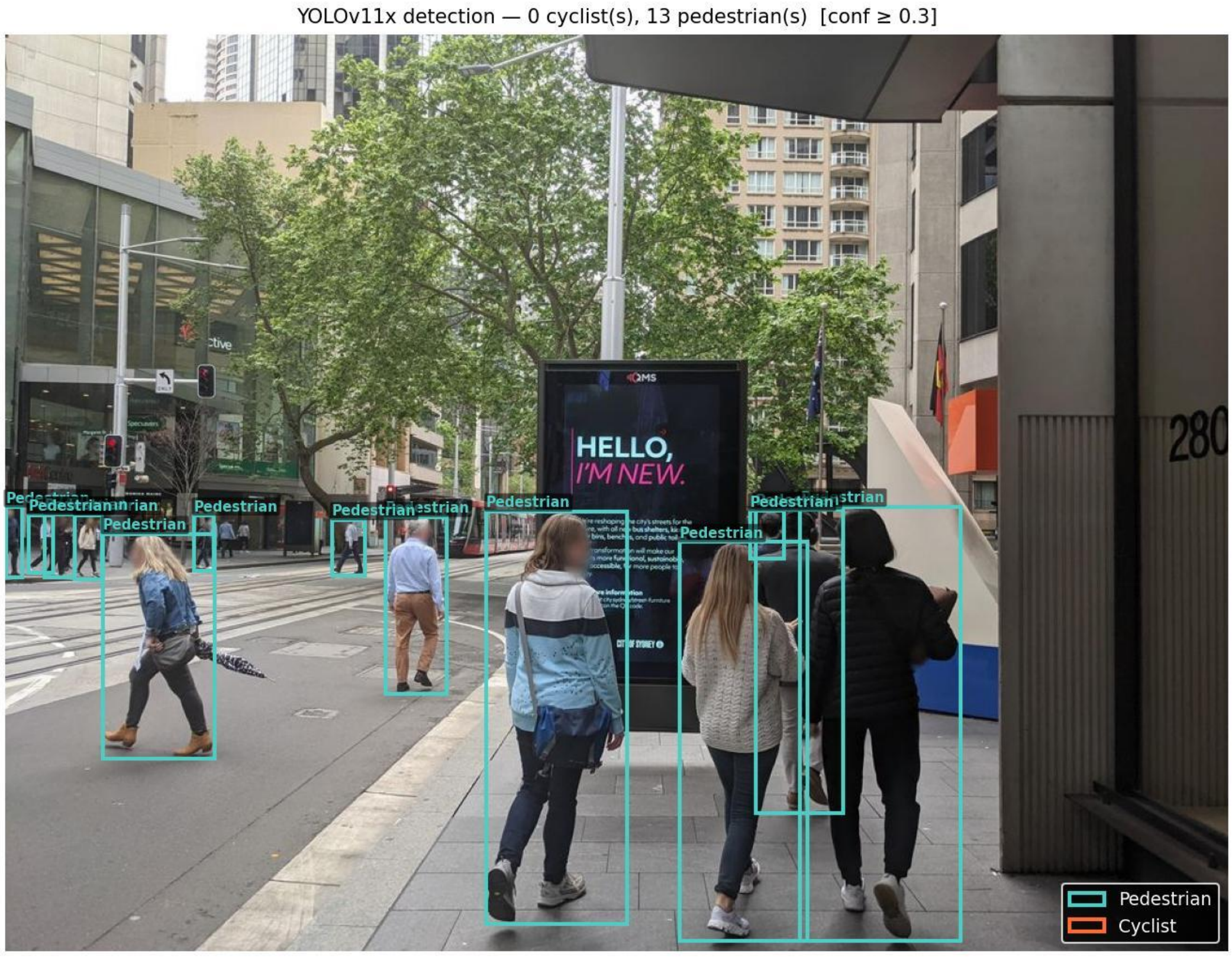

Figure S13. Sydney (Hyde Park), 2022 — pedestrians in public open space. YOLOv11x detected 0 cyclist(s) and 8 pedestrian(s).

YOLOv11x detection — 0 cyclist(s), 8 pedestrian(s) [conf ≥ 0.3]

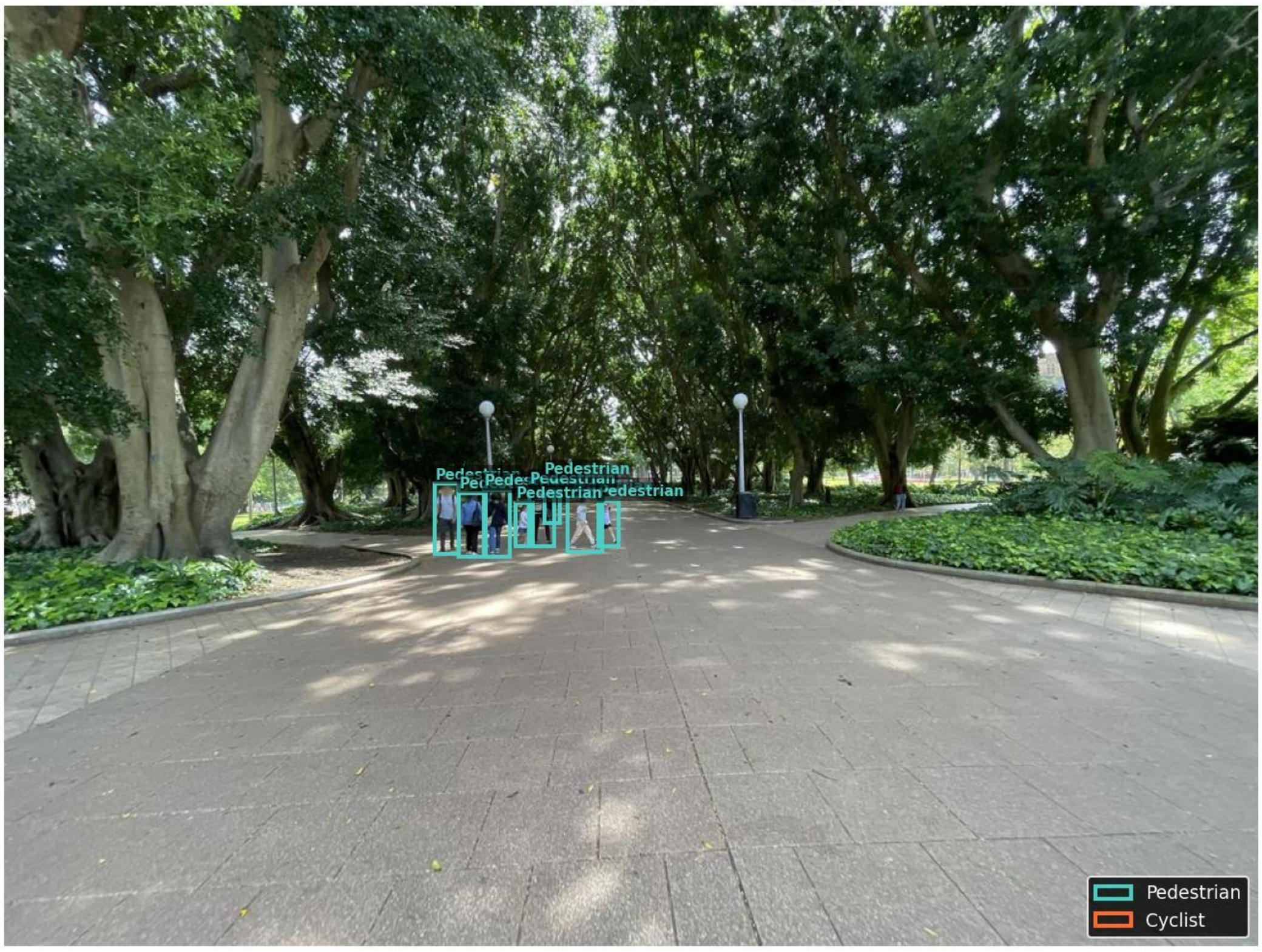

Figure S14. Melbourne CBD (Flinders Street), 2022 — pedestrians crossing at tram stop. YOLOv11x detected 1 cyclist(s) and 8 pedestrian(s).

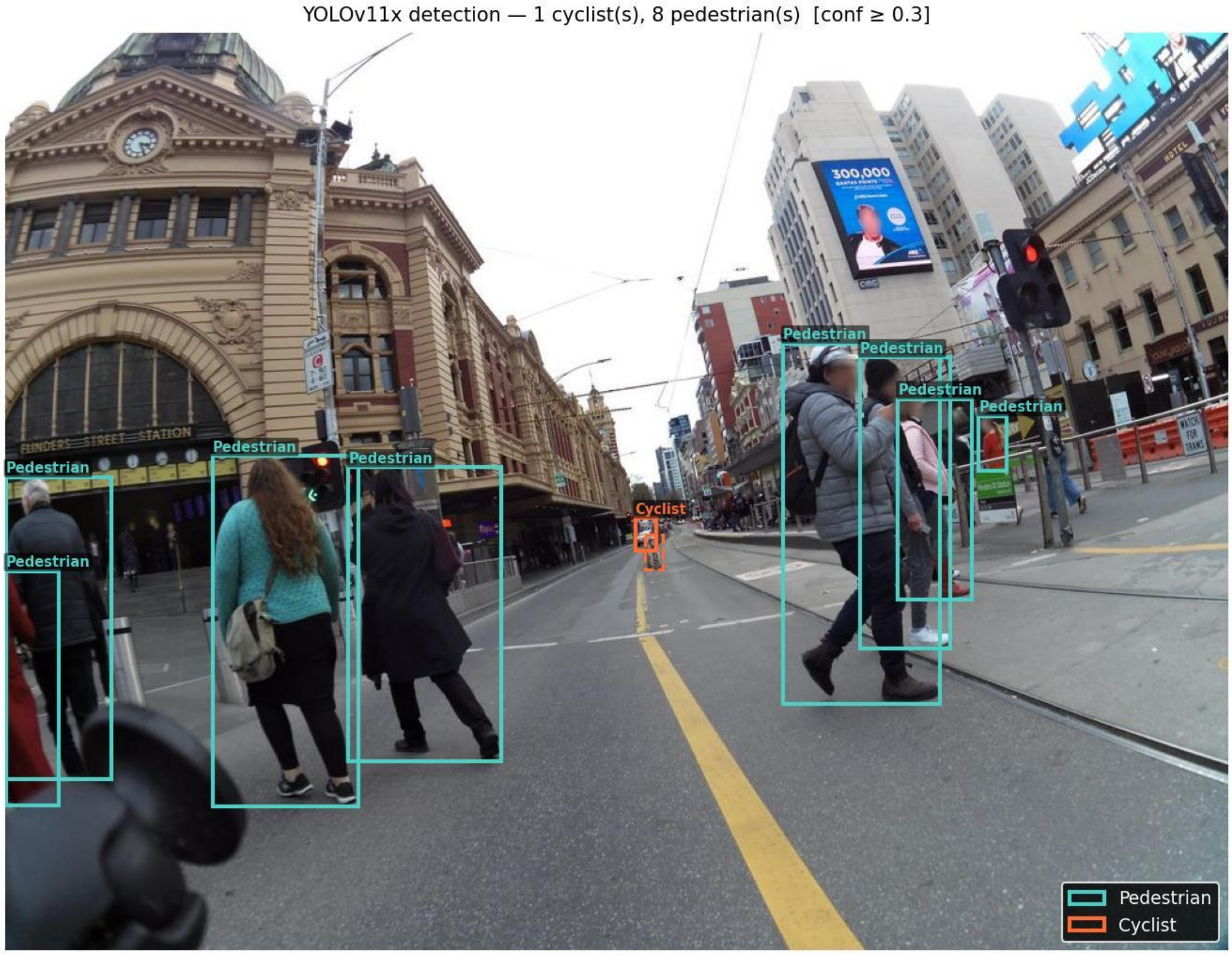

Figure S15. Melbourne inner suburbs, 2022 — cyclist detected on dedicated lane. YOLOv11x detected 1 cyclist(s) and 0 pedestrian.

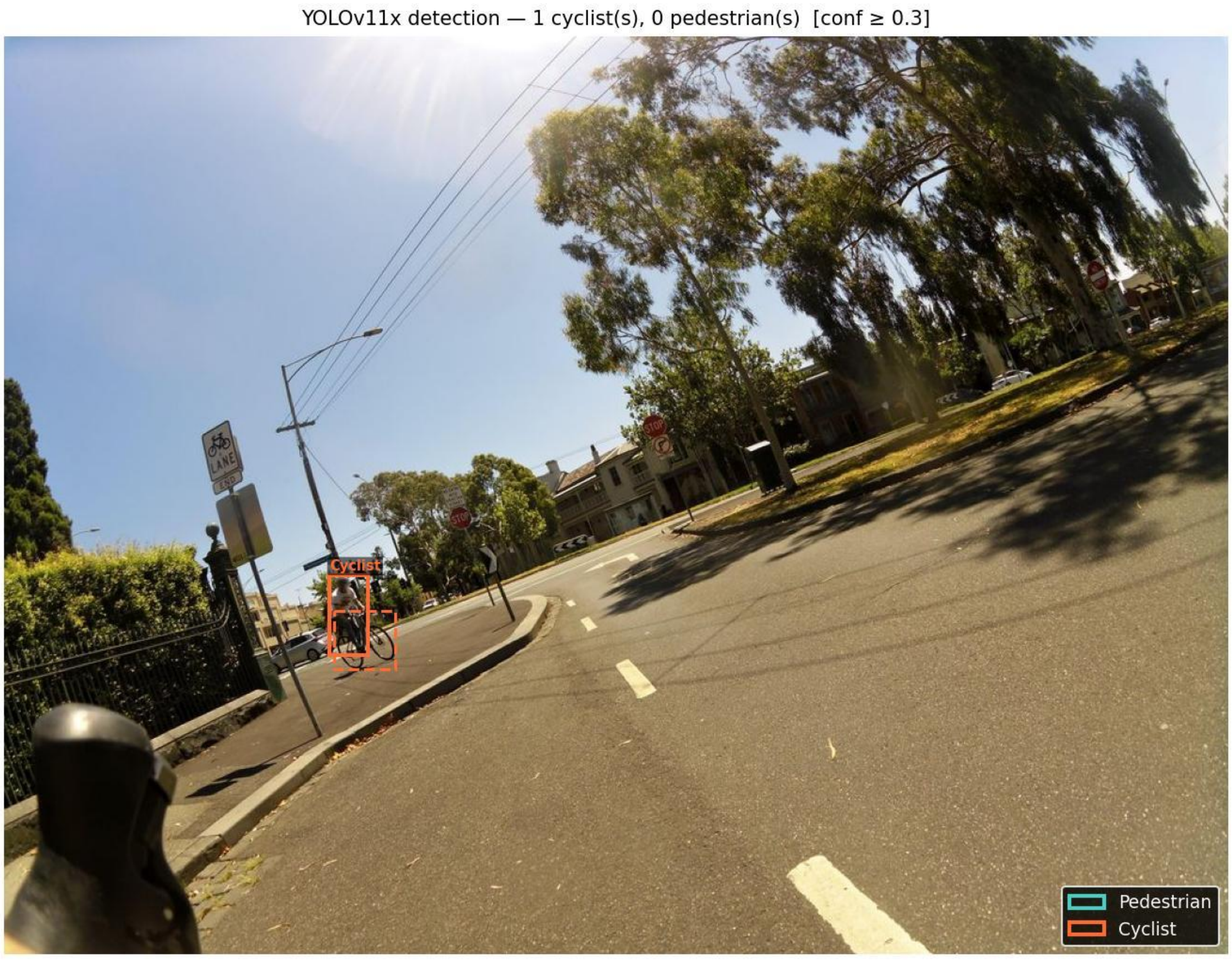

Figure S16. Annual greenhouse gas emissions per capita from food production in Australia and New Zealand by commodity from 2000 to 2023.

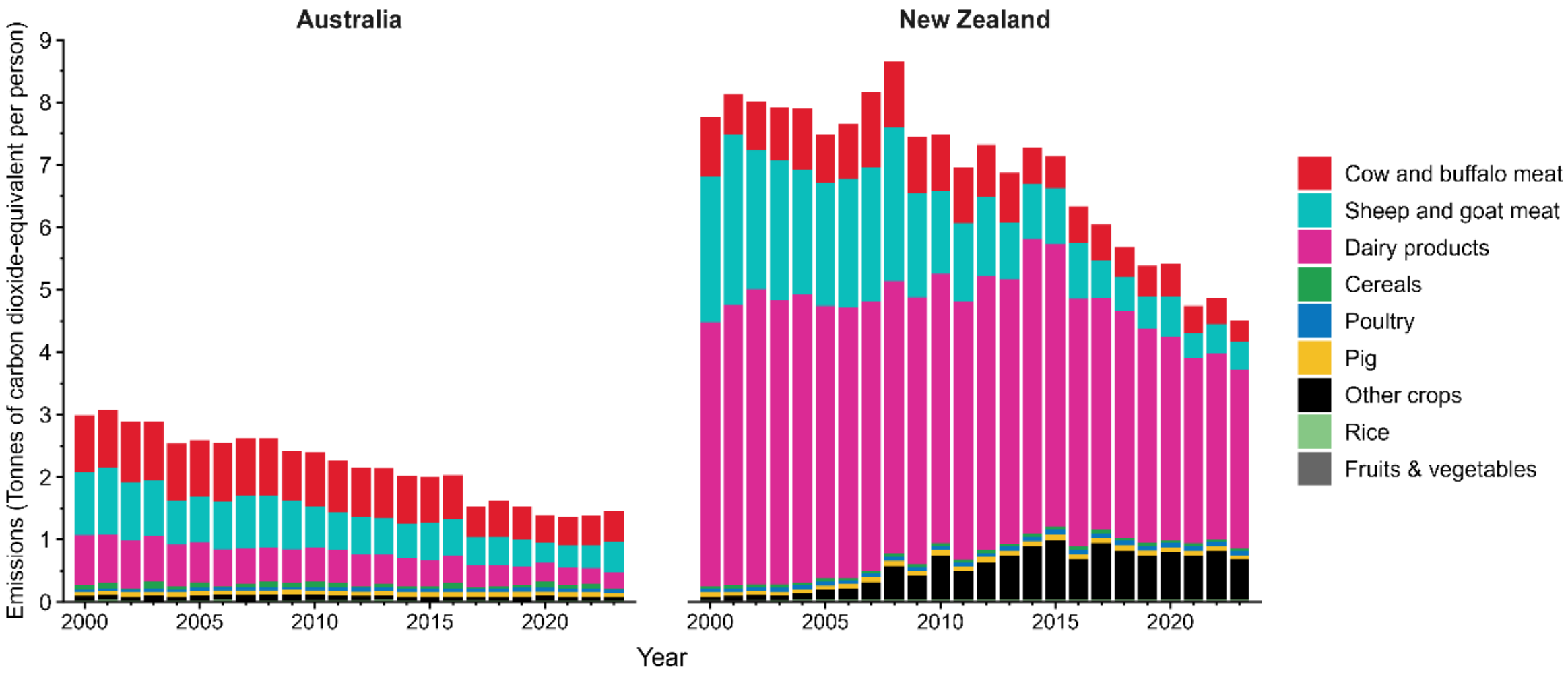

Figure S17. Annual inflation-adjusted total insured economic losses in Australia (base year 2019) from bushfires, cyclones, flooding, hailstorms, storm flooding, and tornados for the period 2000 – 2025 based on the Historical Catastrophe Data of the Insurance Council of Australia.

Data source: Insurance Council of Australia [22].

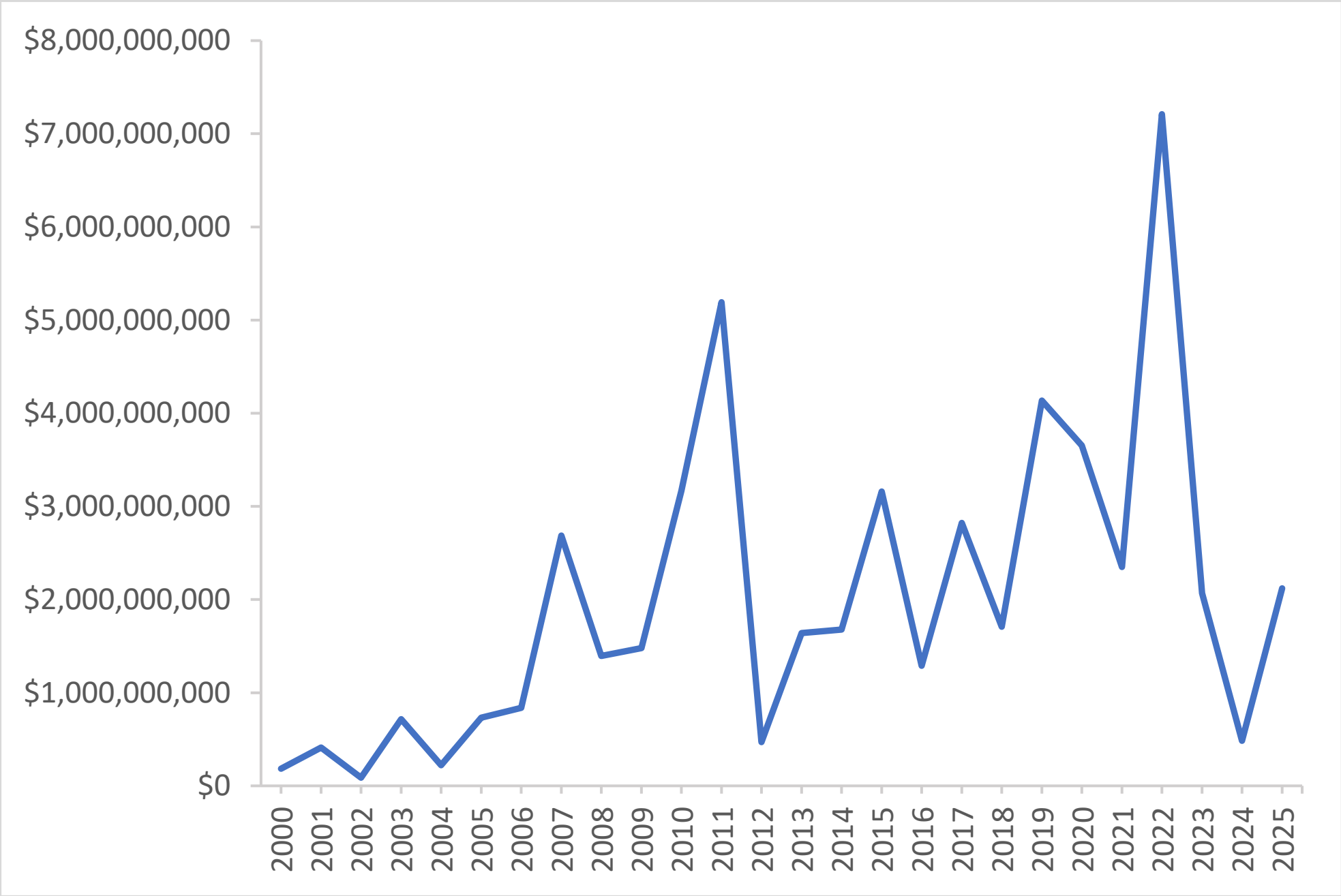

Figure S18. Australia's net carbon price under initial and revised emissions estimates, 2017 - 2024. The figure shows Australia's net carbon price, calculated as the carbon price (zero, as Australia levies no general carbon price) less fossil fuel subsidies expressed per tonne of greenhouse gas emissions. The indicator combines fossil fuel subsidy data from the Organisation for Economic Co-operation and Development (OECD) with greenhouse gas emissions data from Australia's National Greenhouse Gas Inventory. The blue line shows the net carbon price calculated using Australia's National Inventory Total greenhouse gas emissions as first reported in each year's own December Quarterly Update ("Initial publication"). The red line shows the same indicator recalculated with emissions as re-reported in the following year's update ("Revised in subsequent report"); the red series ends in 2023 because no subsequent update has yet been published for 2024.

Data sources: Organisation for Economic Co-operation and Development [25], Australian Government [26], Australian Government [29], Australian Government [30], Australian Government [31], Australian Government [32], Australian Government [33], Australian Government [34], Australian Government [35].

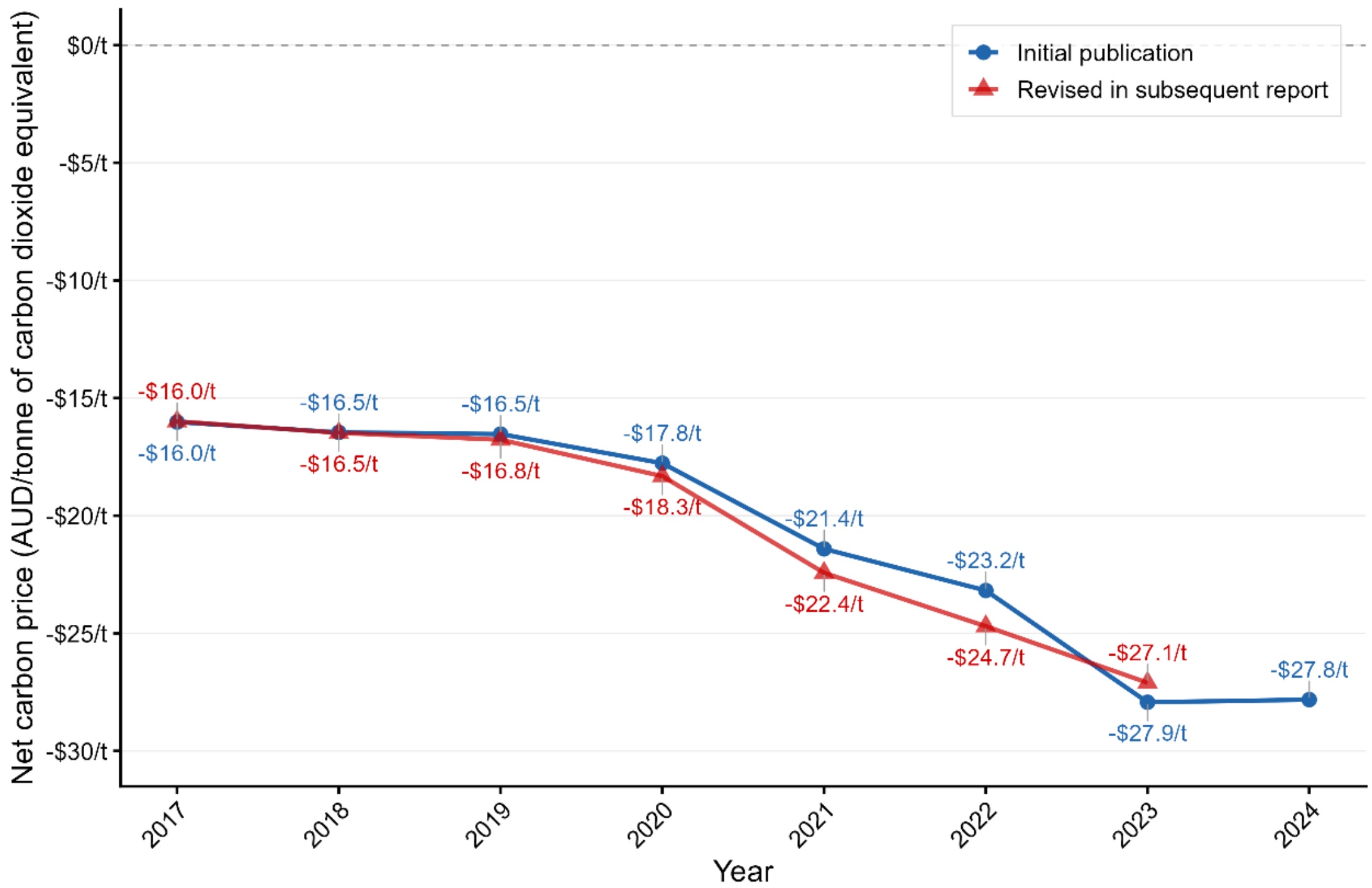

Figure S19. Media coverage of health and climate change on ABC online programs from 2008-2025, Australia.

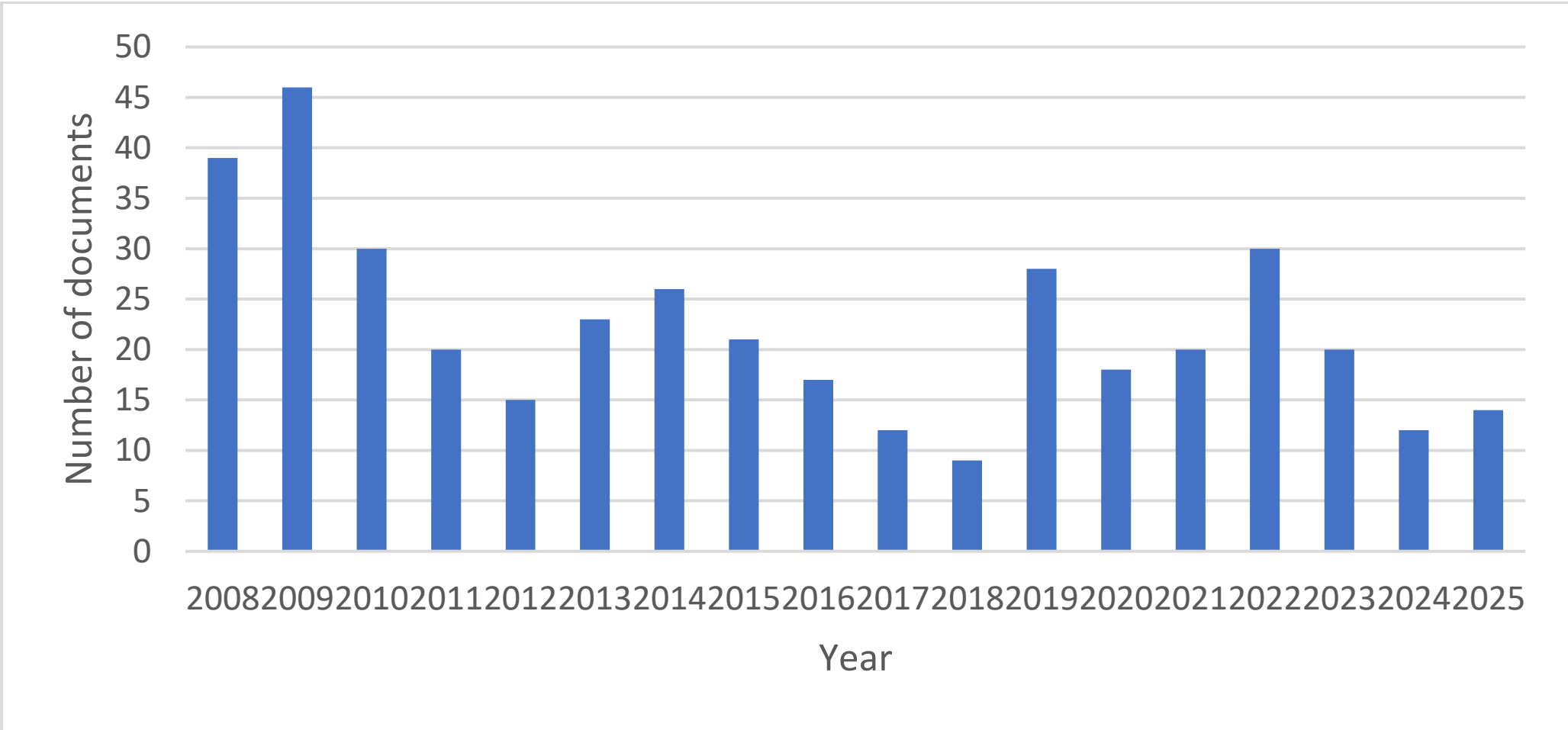

Figure S20. Number of scientific articles on health and climate change in Australia and New Zealand, 2006 to 2025.

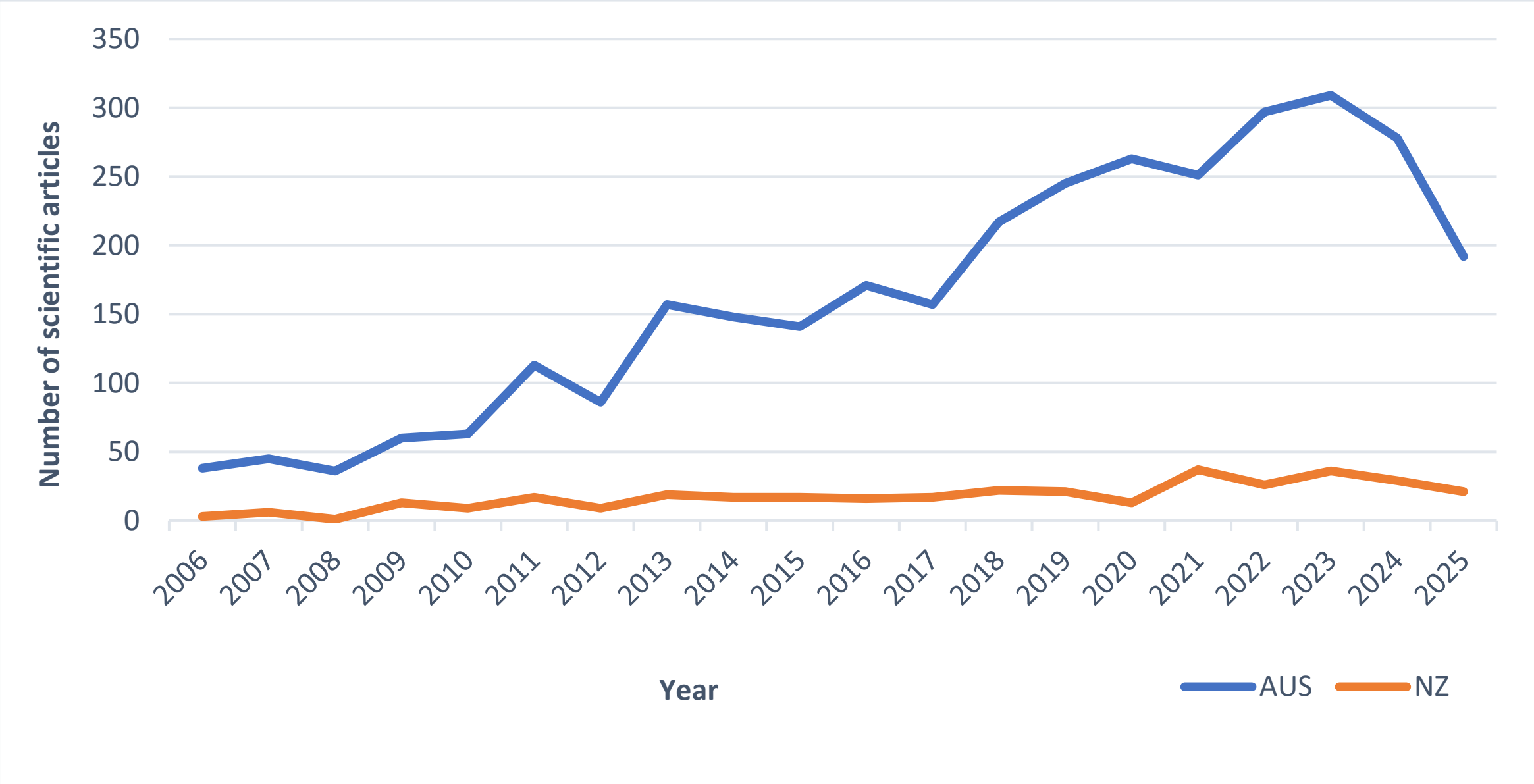

Supplement: Supplementary file 1 [file MJA2-224-0-s001.pdf]
